# Electric fields control static charge during contact-friction-separation

**DOI:** 10.1038/s41467-026-77733-4

**Published:** 2026-10-15

**Authors:** Andris Šutka, Linards Lapčinskis, Artis Linarts, Qiang Sun, Janis Lungevics, Raivis Eglītis, Māris Knite, Dumindu G. Dassanayaka, Amanda V. Ellis, Peter C. Sherrell

**Affiliations:** 1 Institute of Physics and Materials Science, Faculty of Natural Sciences and Technology, Riga Technical University, Riga, LV-1048 Latvia; 2 Faculty of Natural Sciences and Technology, Riga Technical University, Riga, LV-1048 Latvia; 3 School of Science, RMIT University, Melbourne, Victoria 3001 Australia; 4 Institute of Mechanics and Mechanical Engineering, Faculty of Civil and Mechanical Engineering, Riga Technical University, Riga, LV-1048 Latvia; 5 Department of Chemical Engineering, The University of Melbourne, Parkville, Victoria 3010 Australia

**Keywords:** Polymers, Electronic devices

## Abstract

Contact electrification occurs when two materials rub against one another. This is either a problem, such as when charge builds up during powder processing, or an exploited phenomenon to harvest energy, as is the case in triboelectric nanogenerators. However, why contact electrification occurs remains a topic of hot debate, with ions, adsorbates, electrons and combinations thereof being ascribed the charge carriers. Irrespective of the charge carrier however, there must be a driving force inducing charge to separate and move from one surface to another. Herein, we demonstrate that electric fields, either applied externally or spontaneously generated at an interface can completely alter and even invert surface charge measured after contact electrification in polymer-polymer contact. Applying an external electric field allows the direct control of both magnitude and polarity static charge on dielectric polymers. These results are coupled to simulations that show the applied electric field is on the order of the fields generated by the flexoelectric effect – providing prima facie evidence of flexoelectric driven contact electrification.

## Abstract (web summary)

Static charging has been long considered an uncontrolled material or surface phenomena. Here, Šutka et al. demonstrate precise control of the magnitude and sign of static charge via applied electric fields during friction between materials.

## Introduction

Contact electrification (CE) between soft materials is ubiquitous in our daily lives; from a woolen jumper to walking on carpet, we regularly experience the static shocks that come from this surface charging. CE arises from interfacial friction and subsequent charge separation between two materials, with one material tending to acquire a more negative charge and the opposing material tending to acquire a more positive charge^1^, with electrostatic breakdown and discharge further muddying the picture^2–4^. However, the driving force for contact electrification in polymers remains a quandary^5,6^. Recent work has highlighted the contribution of charged molecular fragments or organo-ions (due to bond scission or adsorbate ionisation) to observed contact electrification^7–9^, although electron transfer, ion transfer, adsorbate-mediated charging, and material transfer have all been proposed as possible charge-transfer pathways in contact electrification^6,10,11^. However, why these charges separate to assemble on opposing surfaces remains unclear^5,6^. Without a clear understanding of this driving force, designing materials and devices to exploit contact separation remains arbitrary. While the field of triboelectric nanogenerators (TENGs), which exploit CE, has exploded in publications, most focus on device engineering approaches to improve electromechanical conversion and rarely comment on electromechanical conversion efficiency. We contend this iterative approach is simply due to the poor understanding of the appropriate design of polymer contact electrification materials.

Several driving forces have been proposed for charge transfer in polymer triboelectrification (TE), such as electronic properties, acidity/basicity, zeta potential, and hydrophilicity/hydrophobicity^2,7,8,12–31^, although zeta-potential is considered relevant only for ion exchange between surface water adsorbate layers^28,32^. Hydrophilicity/hydrophobicity cannot answer these questions, as contact electrification is widely observed between smooth polymers with the same wettability^32^, in addition to contact occurring at temperatures greater than 100 °C, and the occurance of ‘same material’ contact electrification^33–36^. The same logic applies to surface acidity as the same chemical potential is expected on both surfaces of chemically identical materials. Thus, while several charge carriers may contribute to polymer CE, the critical unresolved question is what interfacial driving force separates these charges directionally and spatially during contact.

By contacting identical materials, the surface charge is known to increase with diverging surface roughness or inert filler concentration^10,37–41^. Moreover, it has been found that the smoother polymer in identical polymer contact electrification charges negatively, while rougher positively^40^. This is a general trend observed for many different polymers. In addition, an intriguing trend has also been observed for contacting chemically different polymers – the softer always charges negatively, while the harder positively^42^. The charging trends can be combined – stronger charging can be measured when contacting a hard, rough polymer versus a smooth, soft polymer^43–46^. Although this is an exciting observation, replicated in many polymer systems^43–45^, yet there is no fundamental explanation for why the charged species separates between these contact surfaces.

Recently, Marks and Olson have suggested flexoelectricity as a driving force for the TE charges^5,24–27,47,48^. Flexoelectricity arises due to asymmetric strain gradients in materials, creating local polarisation (or electric fields). While this body of work by Marks and Olson, along with prior work from Mizzi et al.^49^, provides excellent simulations to support the underlying claims, these simulations have not been demonstrated to be the *driving force,* with validation of charging correlation only obtained by atomic force microscopy^27^. This is complex, we must differentiate here flexoelectricity as the source of the measured charge – due to the electric field change during contact-separation, and it’s function as a potential *driving force* – inducing other charges to move. Particularly, when measured triboelectric surface charges are long lived – with reported stability of over 24 h under ambient conditions^50^.

Electric fields have previously been shown to influence CE through work-function modulation in conductive systems^51^, bias-controlled charge transfer in metal–dielectric contacts^52^, and remanent polarisation in ferroelectric polymers^53^. In contrast, this study examines non-ferroelectric insulating polymer contacts, including identical polymer pairs, where conductive charge redistribution, metal–dielectric energy alignment, and stable ferroelectric polarisation are absent.

Herein, we experimentally demonstrate that an external electric field alters the polarity and charge separation in polymer CE experiments. These simple experiments, performed using non-ferroelectric dielectric polymers, show that field-induced polarisation, which occurs during contact at high electric fields, relaxes to leave an opposing, more persistent surface charge, aligning with the observations of Marks and Olson and Mizzi et al.^5,24–27,47^. Together with control measurements and flexoelectric simulations, these results support a mechanism in which interfacial electric fields act as transient driving forces that bias the separation of charged species during polymer CE. This provides an experimental route to test whether native strain-gradient-induced fields, such as those generated at rough or compliant polymer contacts, can drive polymer contact electrification.

## Results

### Electric Field

**Fig. 1:**
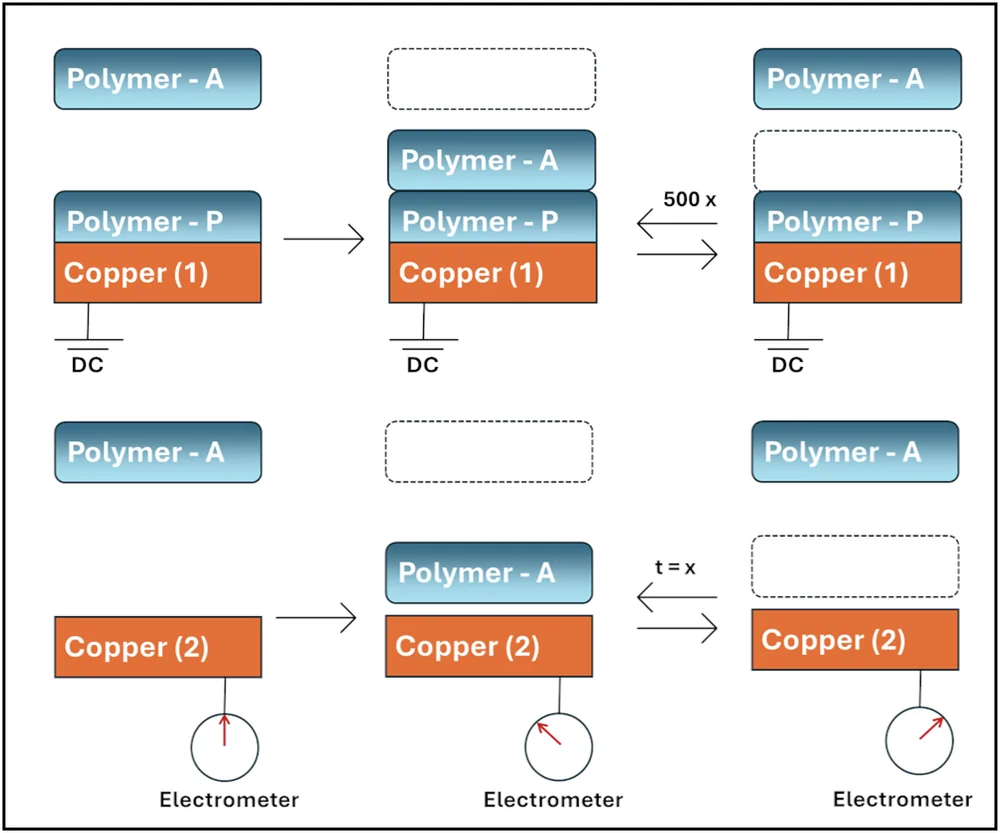
Testing electric fields effects on contact electrification. Schematic of the experimental process; top – contact-electrification is performed whilst a DC electric field is applied to an external electrode; bottom – the copper electrode is changed to a new electrode connected to an electrometer and the electric field (and surface charge) is measured via non-contact oscillation with respect to this new electrode.

To study the influence of electric field on CE interfacial polarisation, a simple experiment was designed, whereby a polymer is directly contact-separated with an identical polymer-metal surface whilst a large electric potential (in the range of +3 kV to –3 kV) is applied to the metal (Fig. 1, top – repeated 500x). For the proof of principle experiment, polystyrene (PS) was chosen as the exemplar material as it is commonly used for CE and TENG devices^44,54,55^, and can stabilise radicals which are important for CE stability^50,56,57^. The experiment consisted of: 1) a polymer sample to be probed; 2) a metal electrode to apply an external electric field; and 3) a protective polymer layer, which is chemically and physically (topographically) equivalent to 1. This experiment (schematically depicted in Fig. 1) was mounted on a mechanical testing system and connected to a power supply and electrical measurement system, enabling the correlation of electrical and mechanical signals. The applied potential is then switched off (and the metal plate grounded), as the polymer is separated from the electrode, with the current induced by motion of the ‘contact electrified’ polymer surface measured by a new electrode during ‘non-contact oscillation’, with the separation distance oscillated between 0.1 mm and 1.1 mm away from the electrode (Fig. 1, bottom).

The use of physically and chemically similar (as close to identical as practically possible) materials is critical to removing the influence of the myriad factors known to affect contact electrification. To eliminate the possibility of direct electron injection into the top polymer film (which has been discussed as a mechanism for contact electrification), a protective polymer layer was added to the copper electrode^58^. Importantly, this protective layer prevents direct metal–polymer contact and charge injection, but does not screen the applied electric field. Because the protective and measured layers are dielectric polymers, the applied potential is distributed across the polymer stack, allowing an electric field to reach the polymer–polymer interface during biased contact-separation. A representative Kapton/PS dielectric-barrier simulation confirmed this general behaviour, showing field penetration through the protective layer rather than field screening (Fig. S1).

**Fig. 2:**
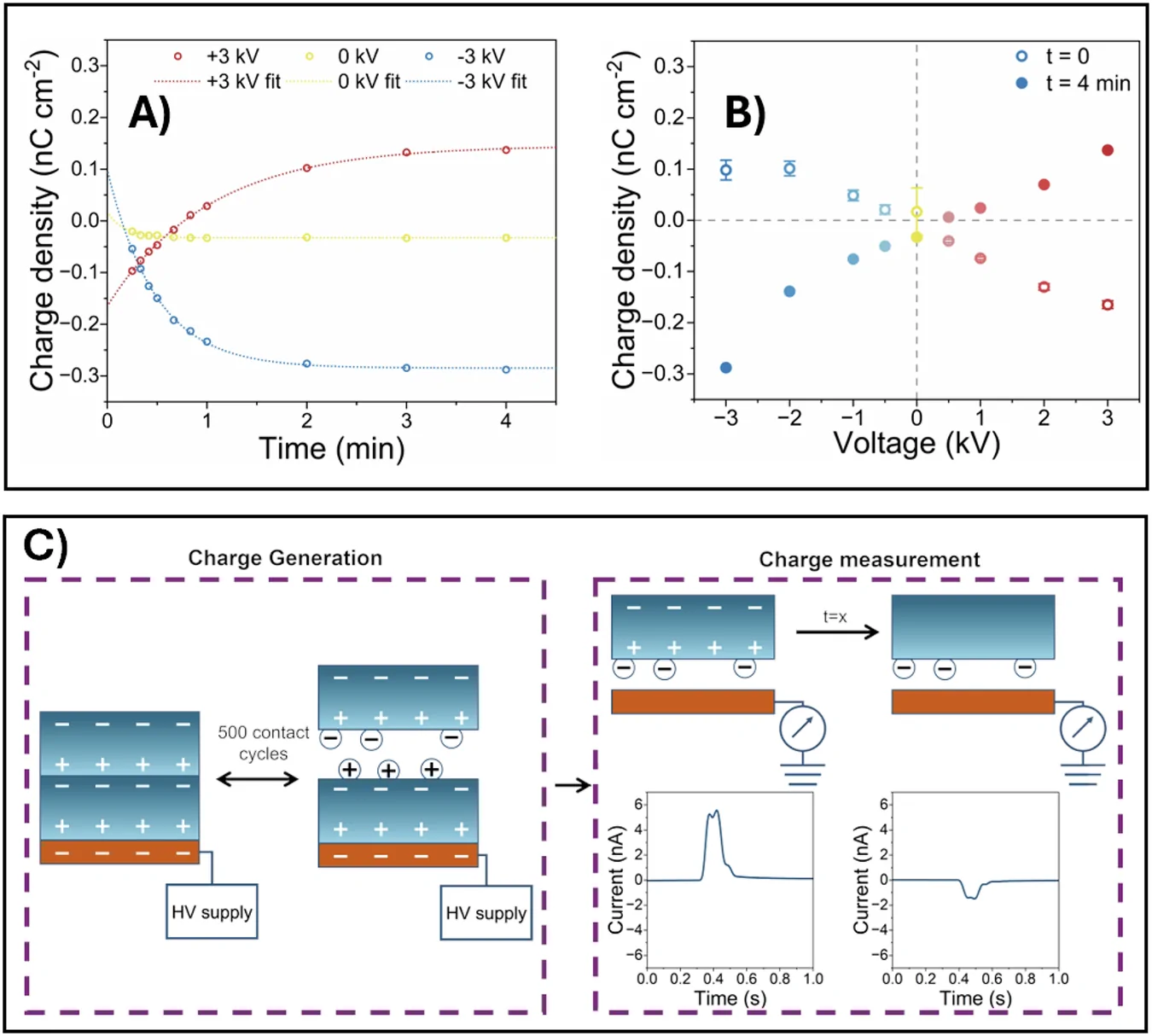
Electric fields drive contact electrification. **A** The measured charge density from the electrometer during non-contact oscillation from same-material contact electrification with polystyrene under three different applied DC electric fields; +3 kV, 0 kV, and −3kV. The charge density is measured over time after the removal of the DC field. **B** The measured charge density as a function of applied DC voltage for polystyrene at t=0 and t=4min (data at t=0 are fitted values, with error bars representing the 95% confidence intervals obtained using the ExpDec1 nonlinear decay model in OriginPro; the fitted curves and details can be seen in Fig. S2 in the supplementary information). **C** Proposed a mechanism for the observations in (**B**), during contact under an applied DC field, the polymers experience a field-induced polarisation effect, which templates transfer of the charge carriers. After the DC field is switched off, mechano-ions partially compensate polarisation; however, the polarisation relaxes, leaving behind the more persistent contact-generated charge carriers, which are measured in non-contact mode. This mechanism explains the charge inversion observed in (**A**) and (**B**).

During surface charge measurement, it is important to note that for a *positive* applied voltage a *negative* measured surface charge is observed at t ≈ 0, and similarly for a *negative* applied voltage a *positive* measured surface charge is observed (Fig. 2A). In Fig. 2A, time refers to the duration of the non-contact oscillation measurement performed after the external DC potential was switched off. Therefore, no additional contact-induced charge is generated during this measurement period; instead, the measured signal reflects the temporal evolution of the surface state produced during the preceding biased contact-separation cycles. The measured surface charge decay was fitted using an exponential function and extrapolated to estimate the initial charge response at t ≈ 0 (Fig. S2). Continued non-contact oscillation and surface charge measurements show that this measured charge decreases over time before inverting, such that at a t = ∞ a *positive* surface charge is measured for a sample which had experienced a *positive* applied DC potential, and a *negative* surface charge is measured for a sample which had experienced a *negative* applied DC potential. This indicates that the measured response contains at least two contributions with different temporal stability. During contact electrification of chemically and physically identical materials, the polarity of the produced surface charge should be arbitrary with no net difference between the samples^59^. This is observed with the 0 kV applied DC voltage yielding no measured surface charge. Expanding this across applied DC voltage ranges encompassing ±3 kV at t = 0 and t = 4 min the same phenomenon was observed for all time points (Fig. 2B). Importantly, the magnitude of the charge at t = 4 min correlates directly to the magnitude of the applied DC electric field. Surface morphology was used to assess interfacial modification and possible voltage-induced damage. RMS roughness changes on initially smooth ITO after biased contact with PS are consistent with contact-induced surface modification or transferred material (Fig. S3, A). SEM and RMS analysis of PS after no contact, 0 kV, +3 kV, and −3 kV contact showed no breakdown-related damage or systematic roughening (Fig. S3, B, C). Since surface heterogeneity can influence same-material tribocharging, the unchanged RMS roughness after 0 kV and ±3 kV contact rules out measurable bias-induced roughening of PS under these conditions^41^. Material transfer was also probed independently by ATR-FTIR in PS–PDMS contacts, where a weak PDMS-related Si–CH₃ band near 1260 cm^−1^ appeared on PS after contact but was absent in PS–PS controls, consistent with a thin/non-uniform transferred layer (Fig. S4). Similar field-dependent charging behaviour was also observed broadly across common dielectric polymer materials including polyether block amide (PEBA), polydimethysiloxane (PDMS), polytetrafluoroethylene (PTFE), and polyimide (PI) (Fig. S5).

Based on these results, we propose a phenomenological model (Fig. 2C) whereby under an applied DC electric field, the polymer and interface undergo field-induced polarisation (Fig. 2C, left). This transient polarisation biases the interfacial charge-separation process during contact-friction-separation, directing charge carriers to move and assemble on opposing interfaces (Fig. 2C, left). After the DC field is turned off, non-contact oscillation is performed with a slight time lag for equipment switching, subsequently the field-induced polarisation relaxes over a period of time. After this polarisation has decayed, the remaining signal is dominated by the more persistent surface charge generated during biased contact-separation (Fig. 2C, right). This interpretation explains why the initially measured charge has the opposite polarity to the later, more stable charge: the early response is dominated by transient polarisation, whereas the later response reflects persistent contact-generated charge. While the authors note that they cannot rule out remnant polarisation effects, such as from electrets or long-lived charged voids^28,60,61^, these effects would be expected to remain with the same measured polarity as at t ≈ 0, and thus cannot be the sole cause of the measured charge density phase inversion over time. These results demonstrate that externally applied electric fields can control and invert the surface charge generated during polymer CE, enabling polymers to be developed such that the magnitude or polarity of surface charge can be precisely tuned (Fig. 2A, B).

Several control experiments were performed to distinguish persistent contact-generated charge from purely field-induced polarisation, electret-like charging, or other transient electrostatic effects. In a gap-control experiment, the same ±3 kV potential was applied while direct contact was suppressed by maintaining an air gap (~100 µm) between the surfaces. Under these conditions, the induced charge decayed toward zero without polarity inversion, showing that electric-field exposure alone produces only a transient polarisation contribution (Fig. S6). Extended non-contact oscillation measurements further showed that, after the initial decay and polarity inversion, the remaining charge persisted for at least 60 min, although it gradually decreased under ambient conditions (Fig. S7). Finally, ethanol rinsing reduced the measured charge close to zero, supporting the surface-localised nature of the persistent charge and making a purely electret explanation less likely (Fig. S8).

### Flexoelectricity

To extend the context of these results and their relevance to typical CE experiments, the design of surfaces expected to undergo flexoelectric polarisation during contact-friction-separation events was undertaken. Crudely, this was achieved via the contact between rough and smooth polymer surfaces^25,40^. The externally applied field should not be interpreted as an exact spatial replica of the native flexoelectric field generated during contact. Flexoelectric fields are highly localised at nanoscale asperities, whereas the applied DC field is macroscopic. Here, the external field is used as a controllable way to showcase that the direction of an interfacial electric field can bias the polarity of persistent charge generated during polymer contact.

**Fig. 3:**
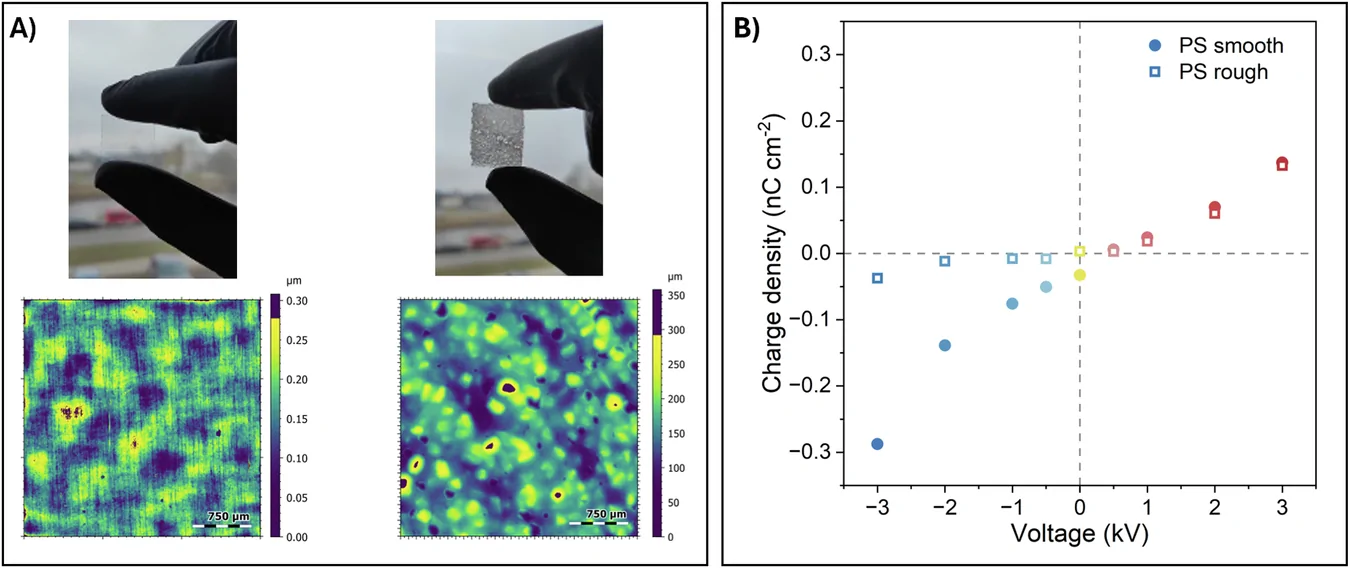
Topography alters electric field effect on contact electrification. The role of surface roughness in observed contact electrification; **A** photo and 3D profilometry scan of the left) smooth PS; and right) rough PS; and **B** the response of rough and smooth PS films due to an applied DC voltage at t = 4 min, analogously to Fig. 2.

Smooth (S_a_ = 0.035 µm, Fig. 3A, left) and rough (S_a_ = 35.42 µm, Fig. 3A, right) PS films were contact-separated under an applied electric field against a chemically equivalent smooth PS film, analogous to the experiment depicted in Fig. 1. Smooth and rough PS are known to demonstrate distinct (opposing) charging tendencies when directly contacting a solid electrode material such as copper (Cu) or indium tin oxide (ITO), with smooth PS tending to gain a negative charge, while rough PS tends to gain a positive charge^55^. Similarly, contact between chemically smooth and rough PS films yields a positive charge on the rougher and a negative charge on the smoother surface^40^. This trend was also confirmed for the present PS samples by KPFM measurements after 500 contact-separation cycles without applied external potential, where smooth PS showed an average surface potential of −2.11 V and rough PS showed an average surface potential of +0.491 V (Fig. S9). Although these post-contact measurements do not directly probe the in-contact flexoelectric field, they confirm the expected roughness-dependent polarity of the resulting surface potential. From the subsequent non-contact oscillation measurement, the effect of the externally applied electric field was observed to differ significantly for smooth and rough PS films. At negative potentials (up to −3 kV), the rough PS demonstrated negligible remaining static surface charge (<−0.03 nC cm^−2^ after 500 cycles of contact-friction-separation, Fig. 2B) while the smooth surface shows almost 10 times higher remaining charge (>0.25 nC cm^−2^ after the equivalent 500 cycles, Fig. 2B). Critically, comparing the charge density at −3 kV applied DC voltage, the smooth PS film data matched closely with that obtained for Fig. 2B, whereas there is a significant reduction in surface charge measured for the rough sample. When the applied DC voltage was swept into the positive regime, the smooth and rough PS samples showed identical results, which will be discussed further in the following section.

**Fig. 4:**
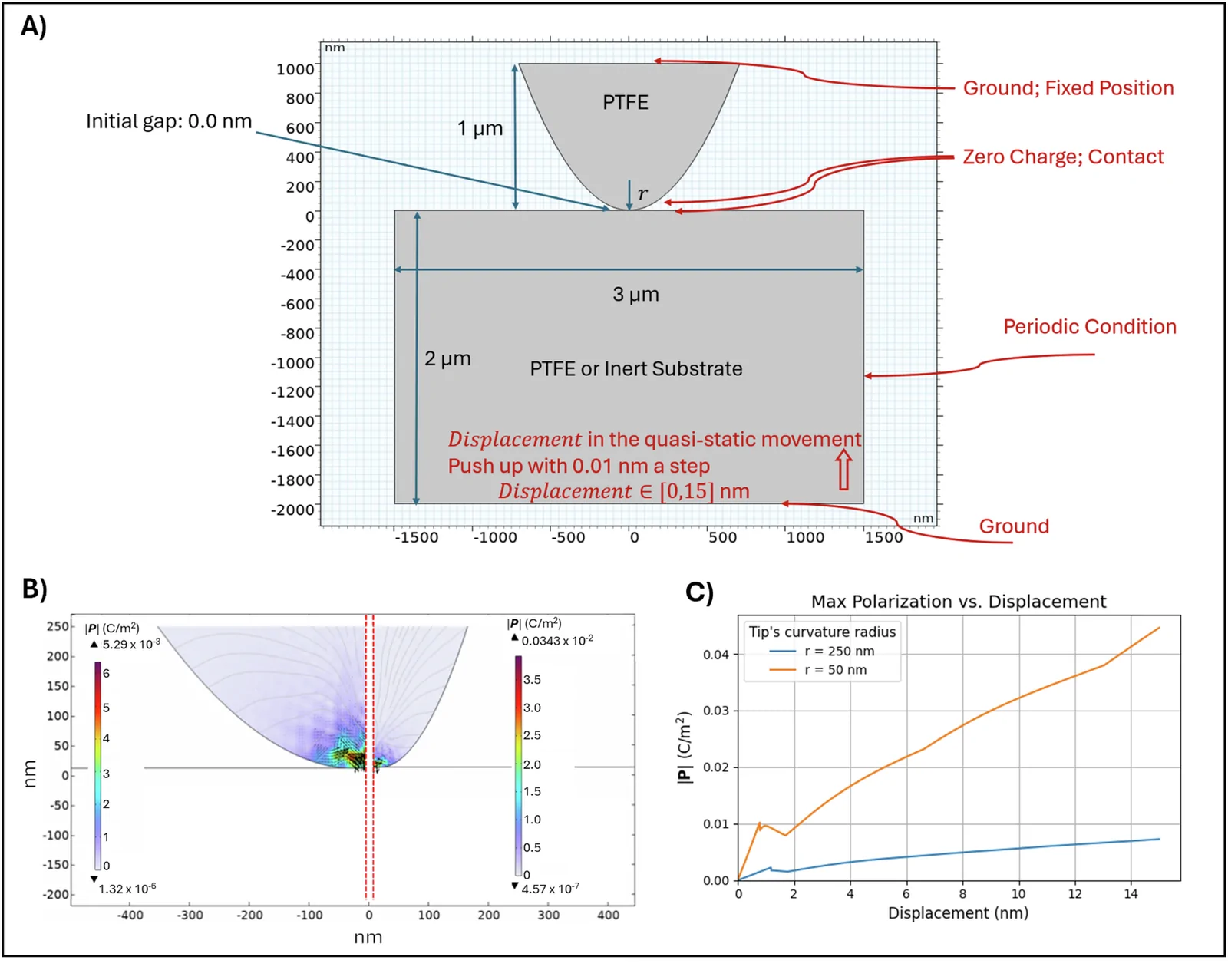
Simulated flexoelectric field effect on contact electrification. Qualitative PTFE based FEM model of roughness-induced flexoelectric fields under compression (PTFE was used because robust reported flexoelectric coefficients are available, whereas corresponding coefficients for PS are not). The x-y axis here refer to the dimensions in nm. The model is intended to indicate field polarity and qualitative trends rather than to quantitatively simulate the PS experiment in Fig. 3. **A** COMSOL simulation set-up and parameters; **B** total polarisation magnitude due to the flexoelectric effect under compression; and **C**  Maximum total polarisation magnitude due to compressive displacement for a ‘rough’ sample with a contact radius of 50 nm, and a ‘smooth’ sample with a contact radius of 250 nm.

To gain an understanding of what is driving this phenomenon, flexoelectric simulations were performed using COMSOL Multiphysics (Fig. 4). Due to the availability of reported flexoelectric coefficients^62–67^, polytetrafluoroethylene (PTFE) was chosen as a model polymer here, however the authors note that nearly all polymers show a flexoelectric coefficient of the same sign, thus while the magnitude of the flexoelectric effect may vary, the trend will remain the same^62–67^. The result in Fig. 4 is used as a qualitative model rather than a quantitative simulation of the PS experiment, since reported flexoelectric coefficients for PS are not available. The model shows that roughness-induced deformation can generate interfacial fields with the polarity required to influence charge transfer, thereby supporting a possible flexoelectric contribution.

The flexoelectric coupling between mechanical deformation and electric polarisation vector, ${{{\boldsymbol{P}}}}$, was described using an isotropic constitutive relation (Eq. 1):

1 $${{{\boldsymbol{P}}}}\equiv {P}_{i}={\epsilon }_{0}\left({\epsilon }_{r}-1\right){E}_{i}{{{\boldsymbol{+}}}}\mu \left[{w}_{1}\frac{{{{\rm{d}}}}{S}_{{kk}}}{{{{\rm{d}}}}{x}_{i}}+{2w}_{2}\frac{{{{\rm{d}}}}{S}_{{ij}}}{{{{\rm{d}}}}{x}_{j}}\right]$$

where $E\equiv {E}_{i}$ is the electric field, ${S}_{{ij}}$ is the strain, ${\epsilon }_{0}$ is the permittivity in vacuum, ${\epsilon }_{r}$ is the relative permittivity of PTFE, μ is the effective flexoelectricity coefficient of PTFE, and ${w}_{1}$ and ${w}_{2}$ are the two weight coefficients for representing volumetric and bending strain gradient contributions, respectively. To isolate the flexoelectric effects clearly, only the strain gradient-induced polarisation was considered as the explicit flexoelectric source within the Electrostatics module. Conversely, the reciprocal coupling, namely the influence of the electric field on mechanical stresses, was deliberately omitted due to its negligible impact on the mechanical response for the material and deformation scales studied here^64^. This assumption aligns with standard practices in flexoelectric modelling, ensuring computational efficiency without sacrificing essential physical accuracy. The model geometric parameters and descriptions are provided in Fig. 4A. The material properties of PTFE in our simulations are: Young’s modulus *E* = 0.4 GPa, Poisson’s ratio ν = 0.46, relative permittivity ε_r_ = 2.1, the effective flexoelectric coefficient *μ* = 5 × 10^−9^ C m^−1^ with dimensionless weights *w*_1_ = *w*_2_ = 0.5 (Young’s modulus was increased to 0.8 GPa for the stiffness comparison in Fig. S11A).

To relate the macroscopic applied field to the simulated flexoelectric fields, their magnitudes were compared using the experimental voltage and film thickness. A separate dielectric-barrier simulation (Fig. S1) shows that the electric field penetrating into the PS layer under a 3 kV applied bias is approximately $2\times {10}^{7}$ V/m. Using a 100 nm contact-adjacent averaging window, the simulated surface-averaged flexoelectric field reaches $4.3\times {10}^{7}$ V/m for the rough geometry (r = 50 nm) and $1.9\times {10}^{7}$ V/m for the smooth geometry (r = 250 nm), demonstrating that the applied field is comparable to the interfacial flexoelectric driving field. In the flexoelectric simulations at 15 nm displacement, the average surface-normal electric field was 4.3 × 10^7^ V m^−1^ for the rougher contact geometry (r = 50 nm) and 1.9 × 10^7^ V m^−1^ for the smoother geometry (r = 250 nm), while the corresponding peak local fields reached 2.3 × 10^9^ V m^−1^ and 3.7 × 10^8^ V m^−1^, respectively. Thus, the externally applied field is comparable to the surface-averaged simulated flexoelectric field, while local flexoelectric fields are strongly amplified at nanoscale asperities.

Simulations of the stress, volume contraction, bending deformation, and flexoelectric polarisation are presented in Supplementary Movies (S1–S3), against an inert (Supplementary Movies, S1, S2) or PTFE (Supplementary Movie S3) substrate, for both smooth (r ≈ 250 nm) (Supplementary Movies S1, S3) and rough (r ≈ 50 nm) (Supplementary Movie S2). A snapshot of the flexoelectric polarisation at 12 nm of displacement in contact with a smooth (Fig. 4B, left) and rough (Fig. 4B, right) sample demonstrates that the rough sample generates an order of magnitude larger flexoelectric polarisation during the friction component of the contact-friction-separation experiments. Further, the flexoelectric polarisation is localised significantly closer to the polymer surface for the rough sample (<20 nm) compared to the smooth sample (<60 nm). This trend holds at all studied deformations (Fig. 4C). The presence of this highly localised field for a rough sample may enable charge transfer of opposingly charged species to occur analogous to the applied DC voltage experiments shown. Similarly, the large stress concentration effects from higher aspect ratio (rougher) features may facilitate interfacial bond scission and charge transfer involving adsorbates, material fragments, or other charged species. Additional biased simulations further showed that the applied field superimposes with the mechanically generated flexoelectric field at the asperity, either reinforcing or opposing the local interfacial field depending on polarity (Fig. S10).

To demonstrate this effect, the compressive modulus of PTFE was artificially varied from 0.4 to 0.8 GPa, and flexoelectric simulations performed (Fig. S11, A). These simulations show that under identical *displacements* (noting that equivalent displacements would require different forces), there was nearly no change in flexoelectric polarisation. In contrast, same-material contact electrification of materials with different compressive moduli showed vastly different measured residual surface charge at t = ∞ (Fig. S11, B), with softer polymers showing an order of magnitude higher residual surface charge. However, this trend should not be interpreted as arising exclusively from Young’s modulus, because comparisons between chemically different polymers inevitably include changes in surface energy, cohesive energy, crystallinity, viscoelasticity, and interfacial adhesion. This limitation was further assessed using chemically similar Ecoflex 00–30 and Ecoflex 00–50 PDMS elastomers. After adding measurements from three independently prepared samples, Ecoflex 00–50 showed lower mean charge densities than Ecoflex 00–30 when ±3 kV voltage was used; however, the values overlapped within experimental uncertainty (Fig. S12). Thus, this comparison suggests the expected direction of the stiffness-related trend but does not clearly resolve a statistically reliable stiffness influence, likely because the stiffness difference between the two grades is small. However, the polarity inversion observed in both materials supports the reproducibility of the effect in chemically similar silicone elastomers. More generally, under the same compressive force (10 N), softer polymers are expected to deform more strongly, increasing strain gradients and the associated flexoelectric polarisation. This was observed via optical absorbance measurements of transparent polymer films during compression (Fig. S13)^68^, which showed significantly greater homogenous surface stress for smooth-smooth contact cases, limiting bond breakage and charge transfer under these conditions. This couples to the known phenomenon of lower cohesive energy density in lower compressive modulus polymers, enabling a greater degree of bond breakage and charge transfer^33,34,42,69,70^.

When considering the lower residual surface charge obtained for rough PS under a negative applied DC voltage (Fig. 3B), it can be considered that the flexoelectric polarisation created by the rough surface is in opposition to the field-induced polarisation from the applied DC voltage in the negative regime, with the surface nature of the flexoelectric field (Fig. 4) limiting charge transfer. However, in the positive regime, the field-induced polarisation from the applied DC voltage (>flexoelectric effect) is aligned with the flexoelectric field but is not greater in magnitude, so no enhancement is observed.

## Discussion

We have demonstrated that an external electric field controls charge transfer polarity and magnitude in polymers. Dielectric polarisation occurs when an external electric field is applied and is responsible for templating the transfer of opposing polarity charged species. This dielectric field-induced polarisation relaxes within minutes (Table S1), while the remaining contact-generated surface charge persists on longer timescales. The schematic representation of both effects occurring during contact-separation under the applied field is demonstrated in Fig. 2C. Importantly, the present results do not require flexoelectric polarisation to be the persistent measured surface charge itself. The strain gradients that generate flexoelectric fields are expected to relax after separation. Instead, we propose that flexoelectricity acts during contact as a transient interfacial driving field that biases the separation and transfer of charged species, such as organo-ions, adsorbate-derived charges, or charged molecular fragments.

These experiments provide an explanation for the long-term dilemma – the formation mechanism of static charge on polymer insulator surfaces. It has been shown convincingly that the charge species on the polymer CE are organo-ions or adsorbates, knowing the dominant charge carrier does not explain *why* negative charge accumulation on soft and smooth polymers, and positive charge accumulation on rough and hard polymers occurs.

Here, the simulation supports the plausibility of a flexoelectric contribution by showing that strain-gradient-induced interfacial fields can reach experimentally relevant magnitudes. Different orientations of strain gradients can be expected on surfaces where the smooth polymers are subjected to tensile deformations, while rough surfaces are subjected to compression deformation^40^. This can be adapted to explain piezoelectric-triboelectric enhancements in hybrid energy harvesters whose performance goes well beyond additive effects.

However, this observation opens another question. Do all polymers have the same sign of flexoelectric coefficients? So far, there are many combinations of polymer pairs studied under contact separation, and in all cases, the smoother film (with less flexoelectric-prone asperities) has charged negatively in identical polymer contact electrification. Similarly, the softer polymer within a contact electrification pair will tend to charge negatively. Flexoelectric coefficients are poorly reported for the polymers. Only a few studies report flexoelectric coefficients of polymers^71,72^. Chu and Salem reported flexoelectric coefficients for chemically different polymers – PVDF, polyethylene terephthalate (PET), polyethylene (PE), and epoxy^71^, and the measured sign of the flexoelectric coefficient was the same.

Understanding the role of interfacial electric fields will enable new designs of systems for both energy harvesting (TENGs), and anti-electrostatic coatings, surfaces, and materials, and opens pathways to establish in-built electric fields to modulate the surface charge of polymers during friction.

## Methods

### Materials

A polyether-block-amide (PEBA) film was first fabricated. For this, a PEBA solution was made by dissolving PEBA (PEBAX 2533, Arkema) in chloroform solution at 50 °C to reach 5 wt%. The solution was cooled down to room temperature and then cast into a PS petri dish and left to dry for 24 h. It resulted in a film with a thickness of ≈ 110 µm. Polyimide (PI) film (25 µm thickness) was procured from Merrem Industriplasts SIA. PS (Sigma Aldrich) was heat-pressed at 285 °C using a 100-μm-thick spacer to obtain smooth PS films. Degassing was performed 2 times during the heat-pressing, and a pressure of 30 atm was applied for 1 min. Rough PS films were obtained by casting a PS solution (20 wt%) in chloroform onto a PE mould designed with a randomly rough surface (Sa surface roughness = 29 µm) as a template. After 24 h of drying, the rough PS film (thickness ≈ 100 µm) was removed from the template. For PTFE films, commercially available Teflon film (30 µm thickness, Merrem Industriplasts SIA) was used. In the case of PDMS (PDMS 30, Ecoflex 00–30) films, the base prepolymer was mixed with the curing agent in a weight ratio of 1:1, respectively, and then cast into a PS petri dish and cured at room temperature for 24 h. Afterwards, the resulting film (thickness ≈ 100 µm) was additionally cured at 120 °C in a lab oven for an additional 2 h to achieve complete crosslinking. Ecoflex 00–50 films were prepared using the same procedure as Ecoflex 00–30, with the base prepolymer and curing agent mixed in a 1:1 weight ratio, cast into films, cured at room temperature for 24 h, and post-cured at 120 °C for 2 h. The physical properties (thickness, roughness, Young’s modulus, dielectric constant) of all films used in this study are provided in Supplementary Information Table S2.

In all cases, the prepared films were cut to the desired size and adhered to the mechanical stage used for contact-separation tests using double-sided adhesive tape.

### Triboelectric measurements

To ensure repeatability and control over various parameters, contact–separation and contactless oscillation were performed using an Instron E1000 material testing machine. Polymer films were adhered to a plastic holder designed for contact-separation tests. A polished copper foil electrode was used as the contact electrode; between the tests, the electrode was thoroughly rinsed with ethanol and dried. The total area under each test was approximately 5 cm². Contact separation with applied voltage was conducted under controlled conditions with a separation distance of 5 mm, a pressing force of 10 N, contact separation frequency of 1 Hz and separation speed of 0.1 m/s. After the last separation step, a 15 s pause was implemented to give time to connect the electrometer for current measurements in contactless oscillation mode. The generated current signals were measured using a Keithley 6514 electrometer connected to a Picoscope 5444B PC oscilloscope system. Contactless oscillations were performed in a range of 0.1−1.1 mm from the sample surface. Oscillation frequency was 0.4 Hz with a separation speed of 0.01 m/s.

Additionally, to exclude the charge injection from the electrodes into the polymer films, the testing was performed with Cu electrodes covered with the same polymer film. The polymer film to be tested was then adhered to the electrode during the contact-separation tests and removed when contactless oscillations started. Other measurement details were left unchanged.

For the gap-control experiment, instead of a contact/compression step, an air gap of around 100 µm was sustained, all while maintaining the same ±3kV applied potential. 500 oscillations were made without direct interfacial contact or repeated friction. After the potential was switched off and the electrode was grounded, the samples were measured using the same contactless oscillation protocol as in the contact-separation experiments.

For long-term stability measurements, contactless oscillation was continued for up to 60 min after biased contact-separation. The oscillation current measurement configuration was kept identical to the standard contactless oscillation protocol.

For ethanol-rinse control experiments, the treated PS surface was rinsed with ethanol and dried (around 3 min) before contactless oscillation measurement. The same measurement geometry and electrical configuration were then used to determine whether the residual surface charge remained after rinsing.

### Optical measurements

The PS films were placed between two glass slides with a piece of glass-ceramic (Sital) used as the background. The diffuse reflectance (DR) spectra were measured using an Ossila spectrometer (Ossila Inc.) and a 50 mm integrating sphere (Thorlabs), with a broadband LED light source (Ocean Optics) and an integration time of 10 s. To measure the DR spectra of the PS films under strain, the two glass slides were compressed using 41 mm binder clips.

### 3D profilometry

Structure of the samples (Rough PS and Smooth PS) was analysed using the AVANT 3D contour and surface roughness measuring system (Mitutoyo, Japan). This system was equipped with an S−3000 roughness detector module, a high-accuracy Y-axis table, and an auto-levelling table. The S−3000 roughness detector module included 0.75 mN detector and a standard stylus 12AAC731 with a cone angle of 60° and a tip radius of 2 μm. The measurement results were postprocessed using MCube Map Ultimate 10.3 software.

### Atomic force microscopy

The surface roughness of polymers (PI, PTFE, PEBA and PDMS) was determined using atomic force microscopy (AFM, Veeco CPII scanning probe microscope). Surface roughness was calculated as the average of topography measurements at three different locations on the sample with a 20 × 20 µm scan area. RMS roughness of PS surfaces was also measured after no contact, 0 kV contact, +3 kV contact, and −3 kV contact to assess whether biased contact caused systematic roughening. RMS roughness of initially smooth ITO was measured before and after biased contact with PS to evaluate contact-induced interfacial modification.

### Kelvin probe force microscopy

KPFM measurements were performed on smooth and rough PS films after 500 contact-separation cycles without externally applied potential (Veeco CPII scanning probe microscope). The average surface potential was determined from the measured regions (5×5 µm) and used to compare the post-contact surface-potential polarity of smooth and rough PS.

### Scanning electron microscopy

SEM imaging was used to compare PS surfaces after no contact, 0 kV contact, +3 kV contact, and −3 kV contact. SEM images were obtained using a Nova NanoSEM 650 microscope at X5000 and X50,000 magnifications.

### ATR-FTIR spectroscopy

Measurements were done using an IRPrestige 21 FTIR spectrometer (Shimadzu), equipped with a PIKE Technologies ATR adapter with a diamond prism. The spectra were obtained in the measurement range of 650–3250 cm^−1^ with a resolution of 1 cm^−1^ and an average of 60 scans. ATR-FTIR spectra were collected from PS surfaces after contact with PDMS under −3 kV, 0 kV, and +3 kV conditions. PS–PS contacted samples were measured as controls. Spectra were analysed in the 1300–1200 cm^−1^ region, focusing on the PDMS Si–CH_3_ deformation band near 1260 cm^−1^. For each condition, five independent spots were measured to assess spatial variability.

### COMSOL simulations

The Solid Mechanics and Electrostatics modules in COMSOL were coupled explicitly via flexoelectric constitutive equations, with polarisation incorporated into the Electrostatics module based on the computed strain gradients from the Solid Mechanics module.

### Model geometry and boundary conditions

Numerical simulations were conducted in a two-dimensional (2D) framework to effectively capture the fundamental physics of flexoelectricity at the interface between contacting materials. The COMSOL Multiphysics software (version 6.2) was utilised, employing coupled multiphysics modules, Solid Mechanics and Electrostatics, to simulate the flexoelectric interactions. The geometric model consisted of a parabolic tip made of PTFE and a flat inert/PTFE substrate. The tip radius of curvature was set to 50 nm or 250 nm, and the substrate had lateral and vertical dimensions of 3 µm and 2 µm, respectively. Initial positioning was such that the tip and substrate were in contact with zero separation. Incremental vertical displacements of 0.01 nm per step were applied quasi-statically to the substrate, spanning a total displacement range of 0–15 nm for the unbiased simulations used in Fig. 4 and Fig. S11A. For the applied-bias comparison in Fig. S10, the displacement ranged from 0 to 15 nm in increments of 0.02 nm. The boundary conditions included grounding and fixed-position constraints on the tip, grounding of the substrate bottom, periodic boundary conditions along the lateral sides, and open-circuit (zero-charge) boundary conditions at the tip-substrate interface. To investigate the superposition of macroscopic and local flexoelectric fields, additional biased simulations were performed by applying an external potential difference of ±90 V across the 3 µm vertical domain, corresponding to a nominal field of 30 MV/m macroscopic field, estimated from the experimental 3 kV bias and the 0.1 mm PS film thickness. Furthermore, a separate 2D electrostatic model was developed to quantify the penetration of the macroscopic electric field through the experimental dielectric barrier. This macroscopic model consisted of a 0.05 mm protective layer (Kapton, ε ≈ 3.4) stacked on a 0.1 mm PS film (ε ≈ 2.6) subjected to a 3 kV external bias.

### Numerical mesh and solver

A hybrid mesh strategy was employed to accurately resolve the local flexoelectric fields. The parabolic tip was discretised using a finely tuned free triangular mesh, ensuring adequate spatial resolution near the critical contact interface region. The rectangular substrate used a mapped mesh with similarly fine discretisation. Multiphysics coupling was implemented, and the numerical solution employed COMSOL’s segregated solver strategy, with the Augmented Lagrangian method chosen to robustly handle the mechanical contact interactions. Simulation sequences began from initial contact at zero displacement, with careful incremental loading to capture detailed variations of polarisation as displacement increased. For the biased nanoscale simulations, the electric field distribution was subsequently solved at each incremental deformation step to extract the superimposed normal electric field and total normal polarisation at the exact contact interface.

### Reporting summary

Further information on research design is available in the Nature Portfolio Reporting Summary linked to this article.

## Supplementary information

Supplementary Information

Description of Additional Supplementary File

Supplementary Movie 1 - COMSOL Simulations of Flexoelectric Fields in 250nm curvature PTFE against an inert substrate.

Supplementary Movie 2 - COMSOL Simulations of Flexoelectirc Fields in 50nm curvature PTFE against an inert substrate.

Supplementary Movie 3 - COMSOL Simulations of Flexoelectric Fields in PTFE-PTFE Contact

Reporting Summary

Transparent Peer Review file

## Source data

Source Data

## Data Availability

The raw data and OriginPro files generated in this study have been deposited in the Figshare database under accession code: https://doi.org/10.6084/m9.figshare.32724315. Source data are provided with this paper.
