# Supplementary material for: Electric fields control static charge during contact-friction-separation
Source: Nat Commun. 2026 Oct 15;17:9971. doi: 10.1038/s41467-026-77733-4 (PMC13672301; doi:10.1038/s41467-026-77733-4)
Supplement: Supplementary file 1 [file 41467_2026_77733_MOESM1_ESM.pdf]

Supplementary Information:

Electric fields control static charge during contact-friction-separation

Andris Šutka[1‡], Linards Lapčinskis[1‡], Artis Linarts[2], Qiang Sun[3], Janis Lungevics[4], Raivis Eglītis[1], Māris Knite[1], Dumindu G. Dassanayaka[3], Amanda V. Ellis[5], Peter C. Sherrell[3,5*]*

[1] Institute of Physics and Materials Science, Faculty of Natural Sciences and Technology, Riga Technical University, LV-1048, Latvia

[2] Faculty of Natural Sciences and Technology, Riga Technical University, LV-1048, Latvia

[3] School of Science, RMIT University, Melbourne, Victoria, 3001, Australia

[4] Institute of Mechanics and Mechanical Engineering, Faculty of Civil and Mechanical Engineering, Riga Technical University, LV-1048, Latvia

[5] Department of Chemical Engineering, The University of Melbourne, Parkville, Victoria, 3010, Australia

[‡] denotes equal contribution

Corresponding author email: andris.sutka@rtu.lv ; peter.sherrell@rmit.edu.au

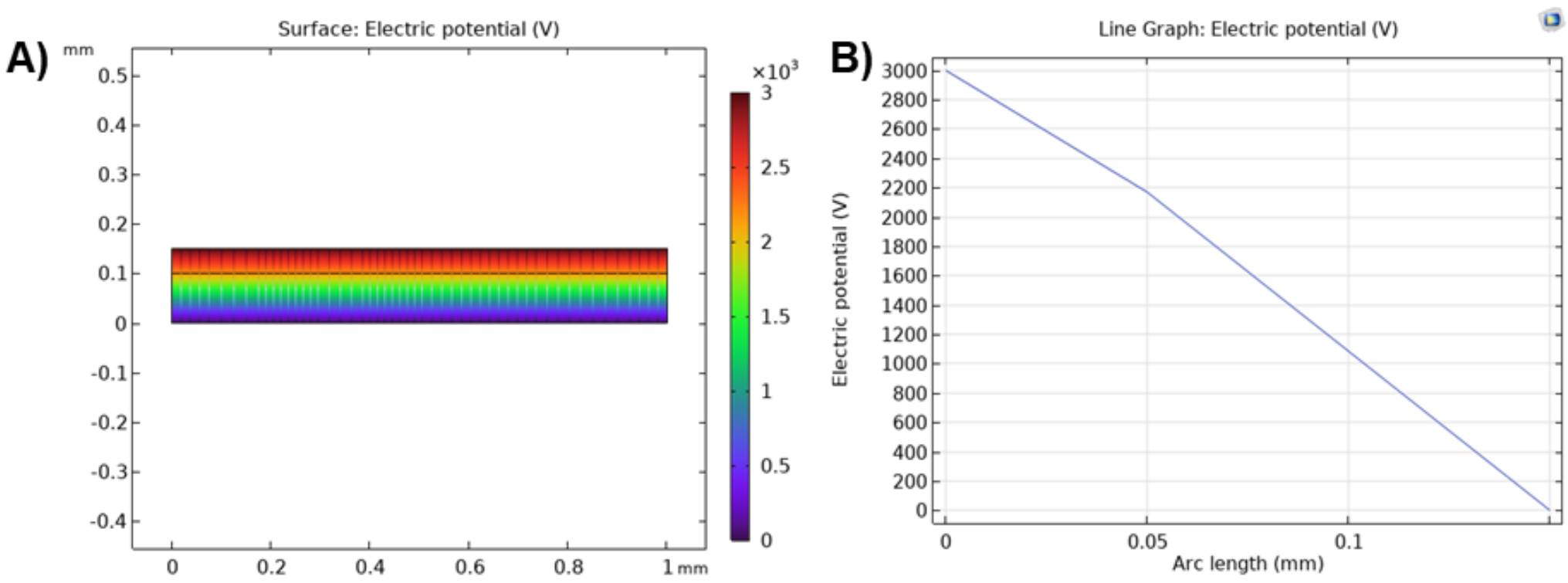

Figure S 1. Electric-potential distribution across the dielectric barrier. A) Dielectric-barrier simulation showing electric potential distribution, B) Electric potential as a function of arc length. Source data are provided as a Source Data file.

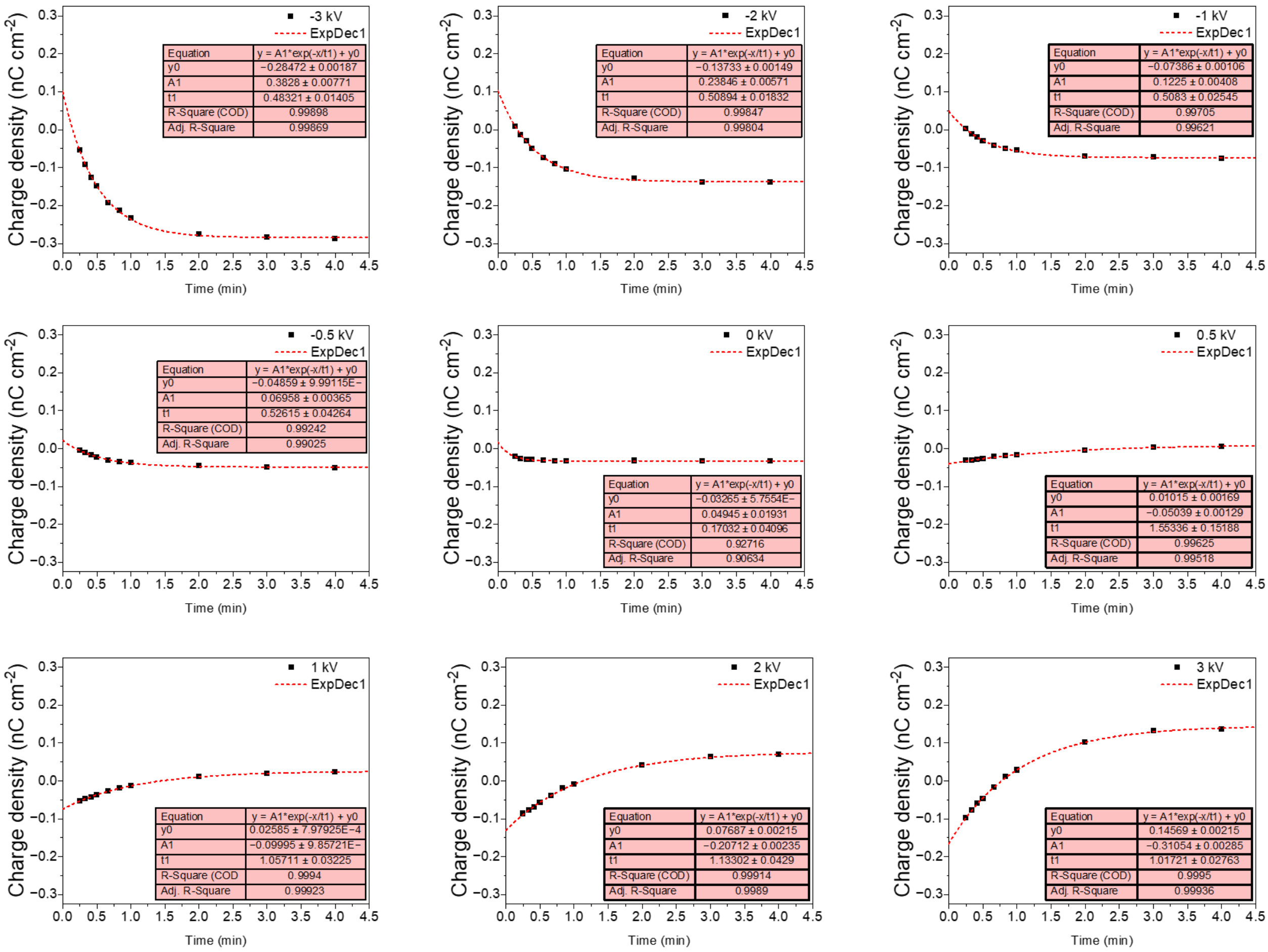

Figure S 2. Exponential relaxation of PS surface charge after biased contact electrification. Exponential decay fittings for PS surface charge change over time after contact electrification experiments under an applied DC electric field, ranging from -3 kV to +3kV. Source data are provided as a Source Data file.

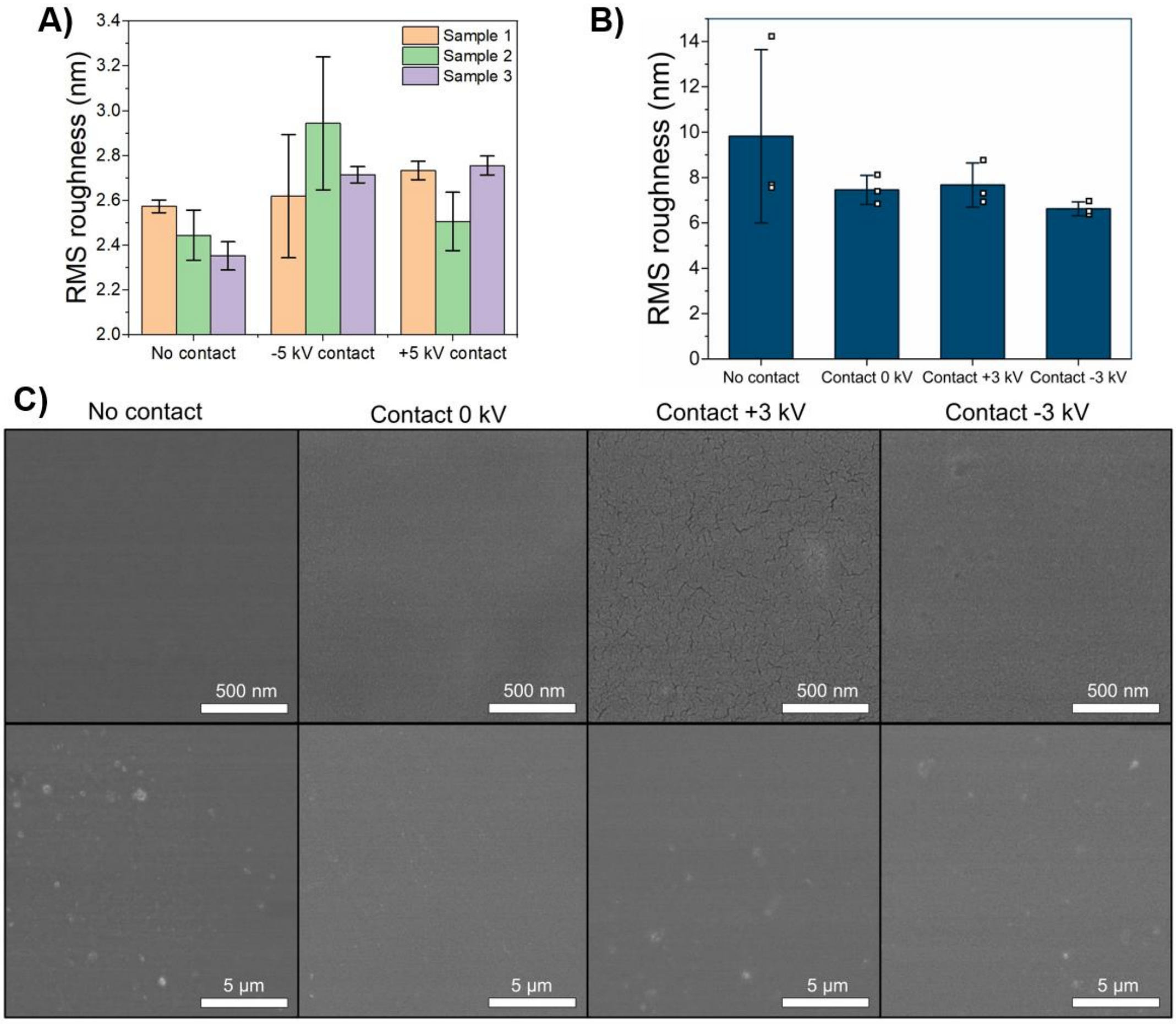

Figure S 3. Surface morphology of ITO and PS after biased contact electrification. A) Root mean square (RMS) roughness of pristine ITO and ITO samples after contact electrification experiments at different applied DC voltages, showing a net increase in surface roughness. Error is calculated by measuring roughnes for the same sample at different places. B) RMS roughness on PS determined by AFM at three spots for each sample. Measurement area – 20×20 µm. C) SEM images of sample that hasn't been contacted, sample that was contacted against another PS without electric potential, and samples that were contacted against another PS with electric potentials of ±3 kV at two magnifications. Source data are provided as a Source Data file.

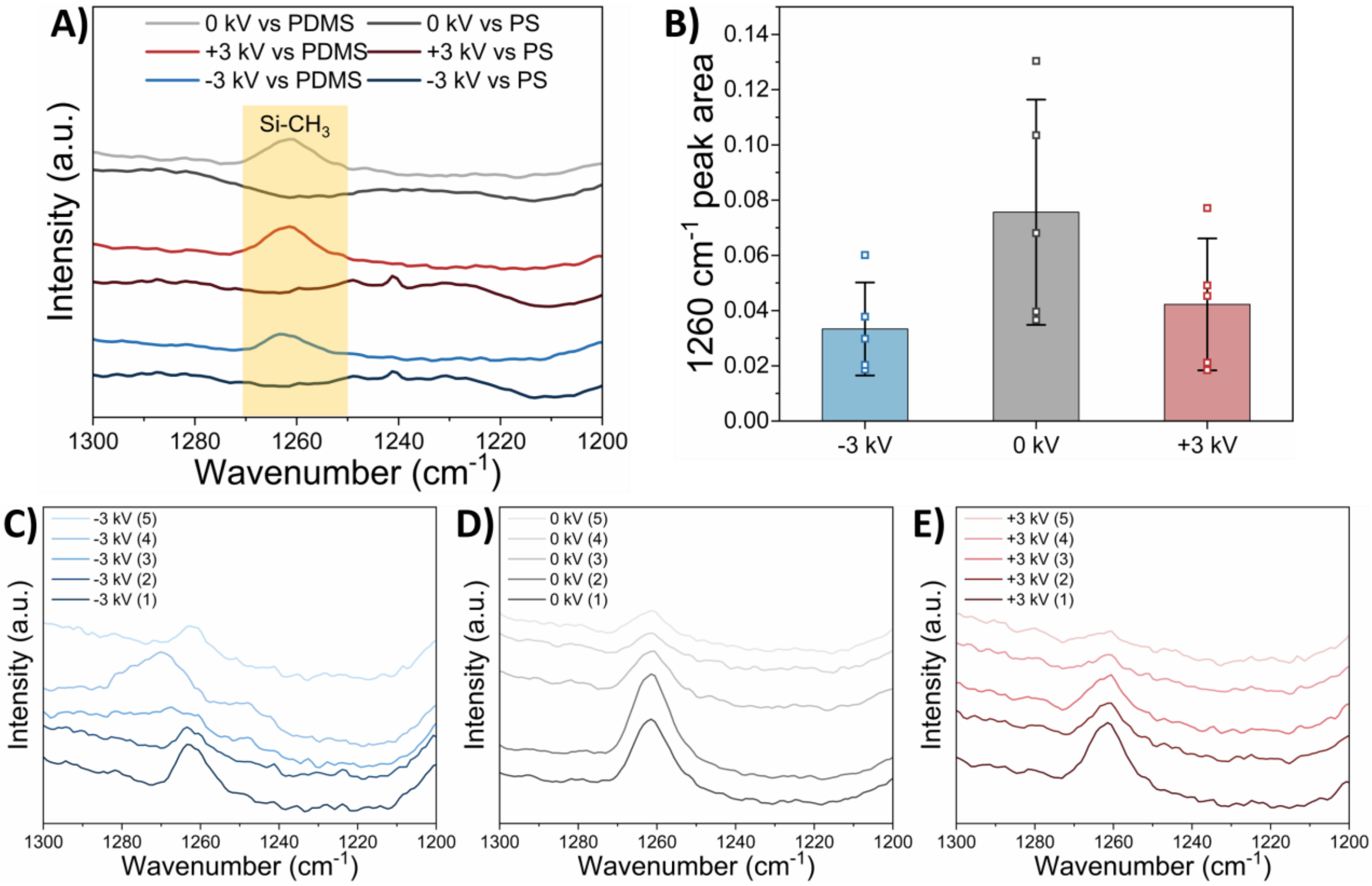

Figure S4. ATR-FTIR analysis of material transfer from PDMS to PS. A) ATR-FTIR spectra (1300-1200 cm^{-1}) comparison for PS contacted either against another PS or PDMS with (±3 kV) and without (0 kV) electric potential applied; b) calculated peak area for the characteristic PDMS peak at 1260 cm^{-1}; c) ATR-FTIR measurements at five different spots for PS contacted against PDMS using -3 kV potential; d) ATR-FTIR measurements at five different spots for PS contacted against PDMS without potential e) ATR-FTIR measurements at five different spots for PS contacted against PDMS using +3 kV potential. Source data are provided as a Source Data file.

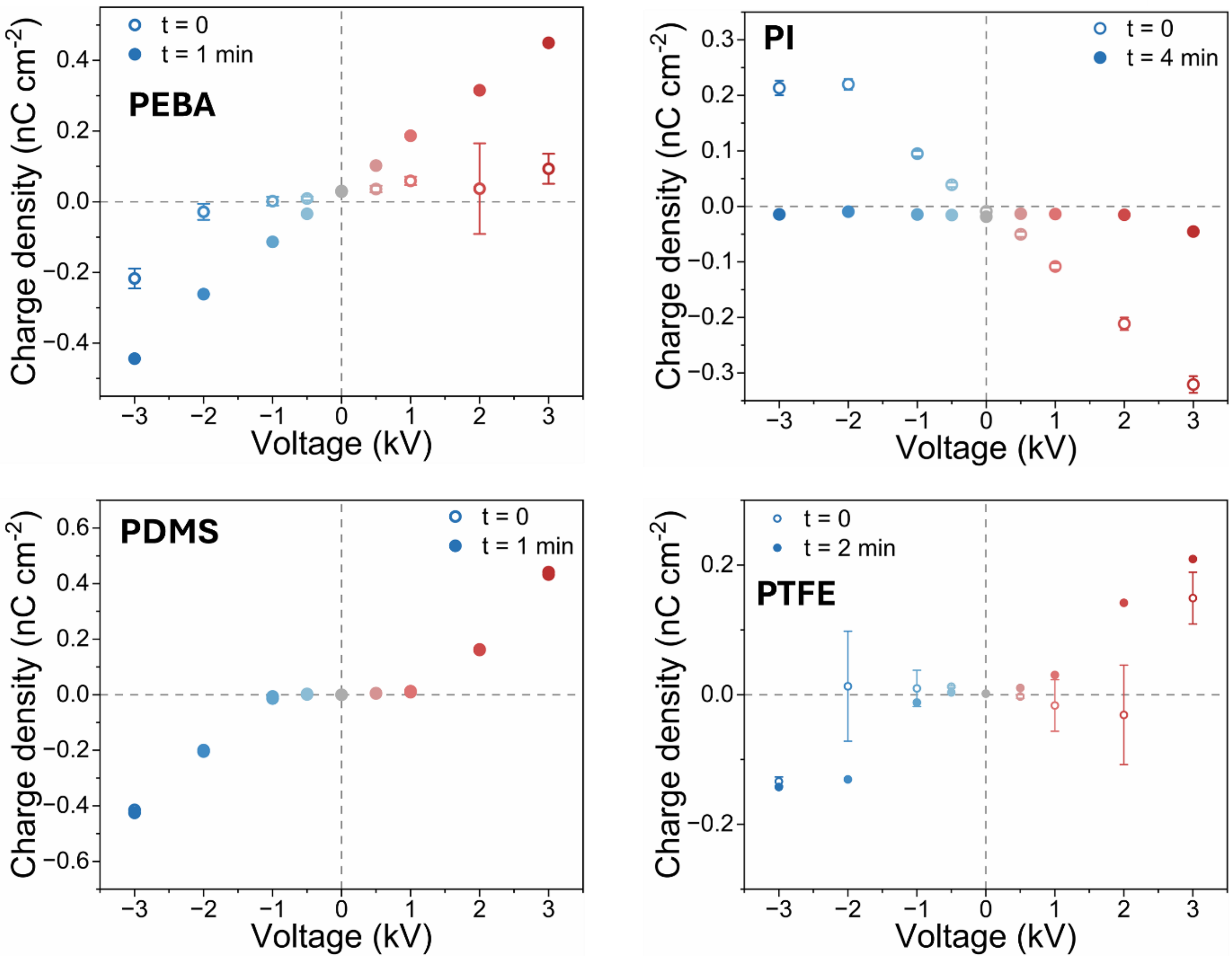

Figure S5. Bias-dependent charging of different polymers. Applied DC voltage experiments with a range of polymers, PEBA, PI, PDMS, and PTFE. Data at t = 0 are fitted values, with error bars representing the 95% confidence intervals obtained using the ExpDec1 nonlinear decay model in OriginPro. Source data are provided as a Source Data file.

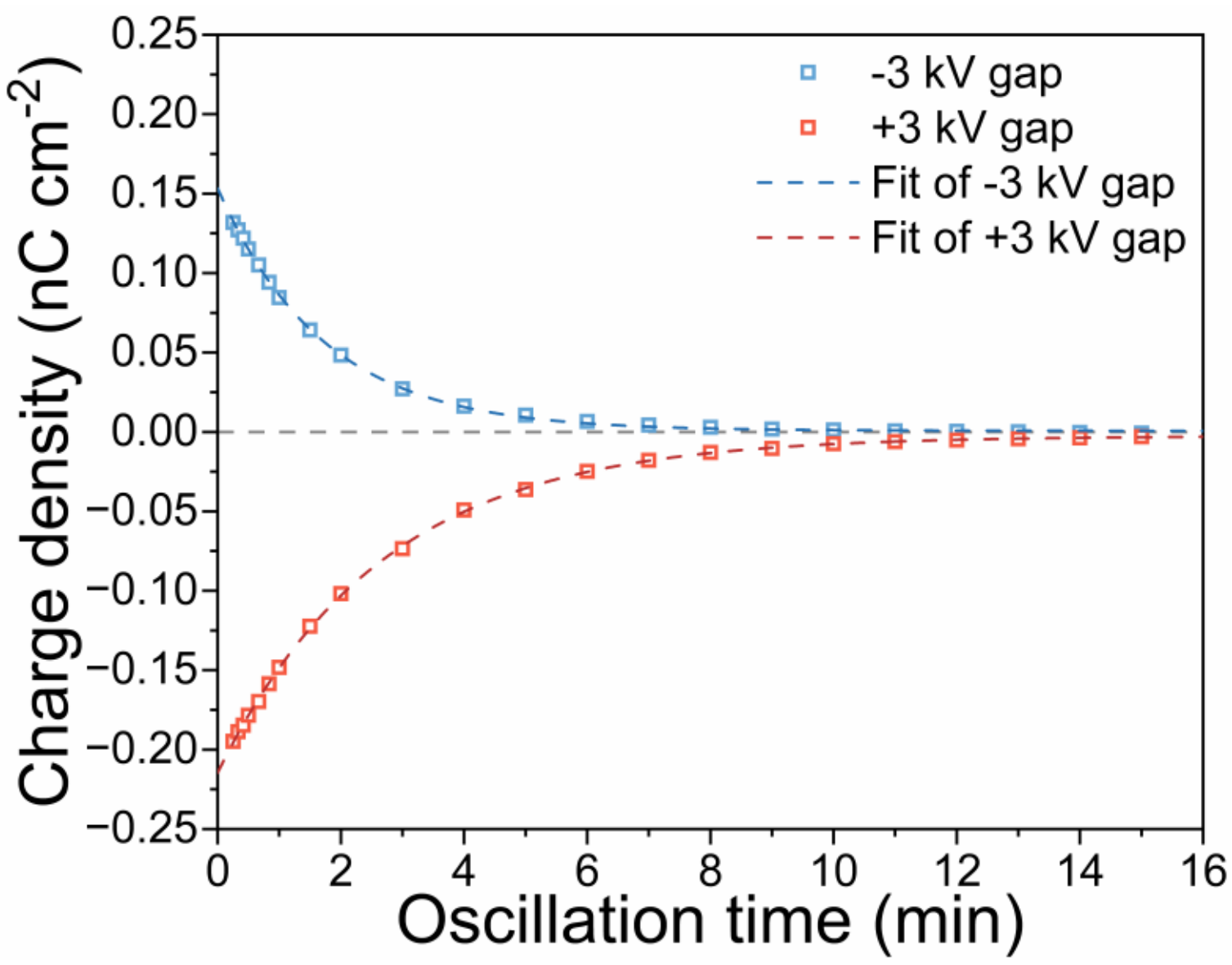

Figure S6. Charge relaxation following the non-contact gap control experiment. Charge density as a function of oscillation time after "gap" control test. Source data are provided as a Source Data file.

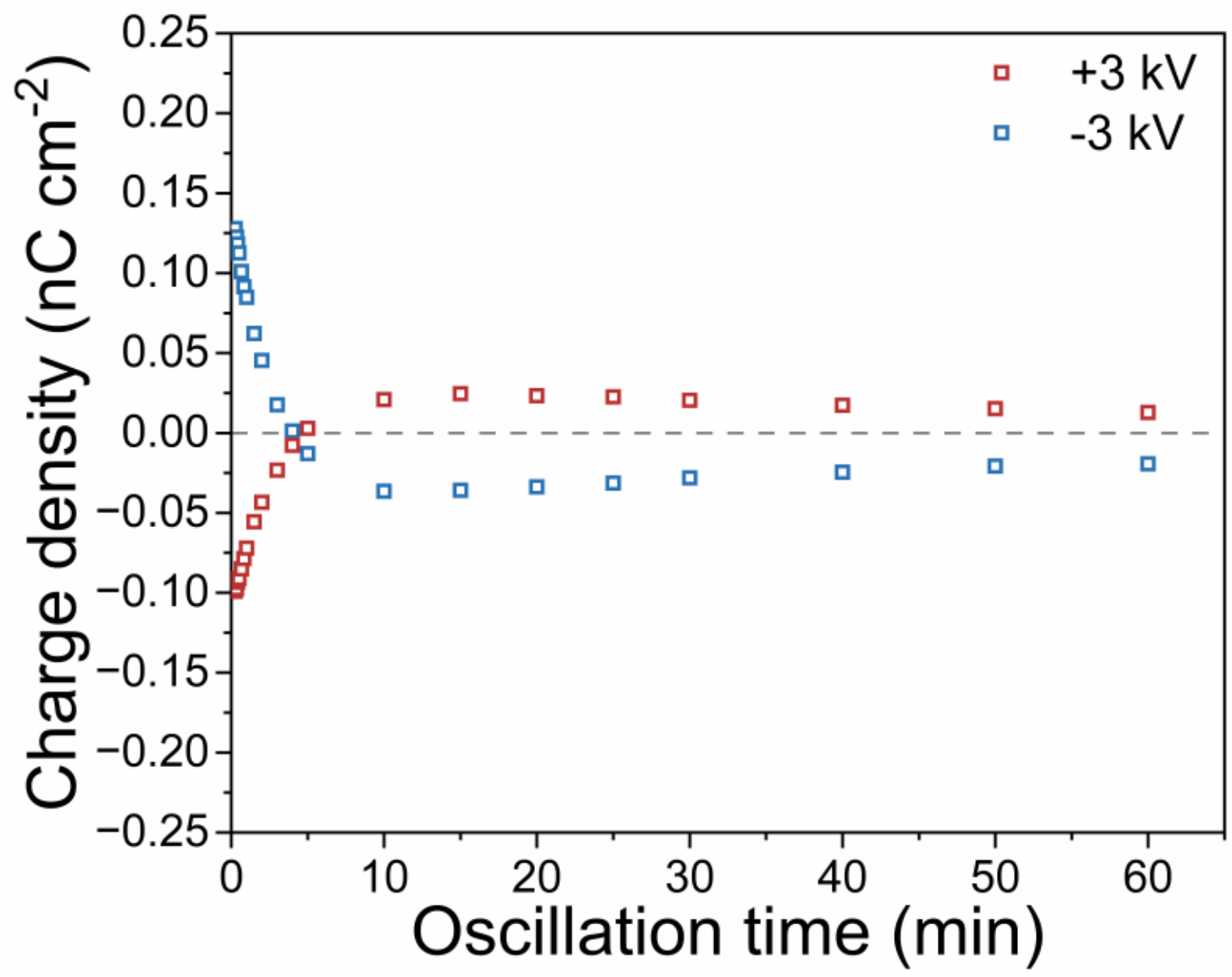

Figure S7. Long-term charge stability during non-contact oscillation. Charge density as a function of non-contact oscillation time in the long-term stability test. Source data are provided as a Source Data file.

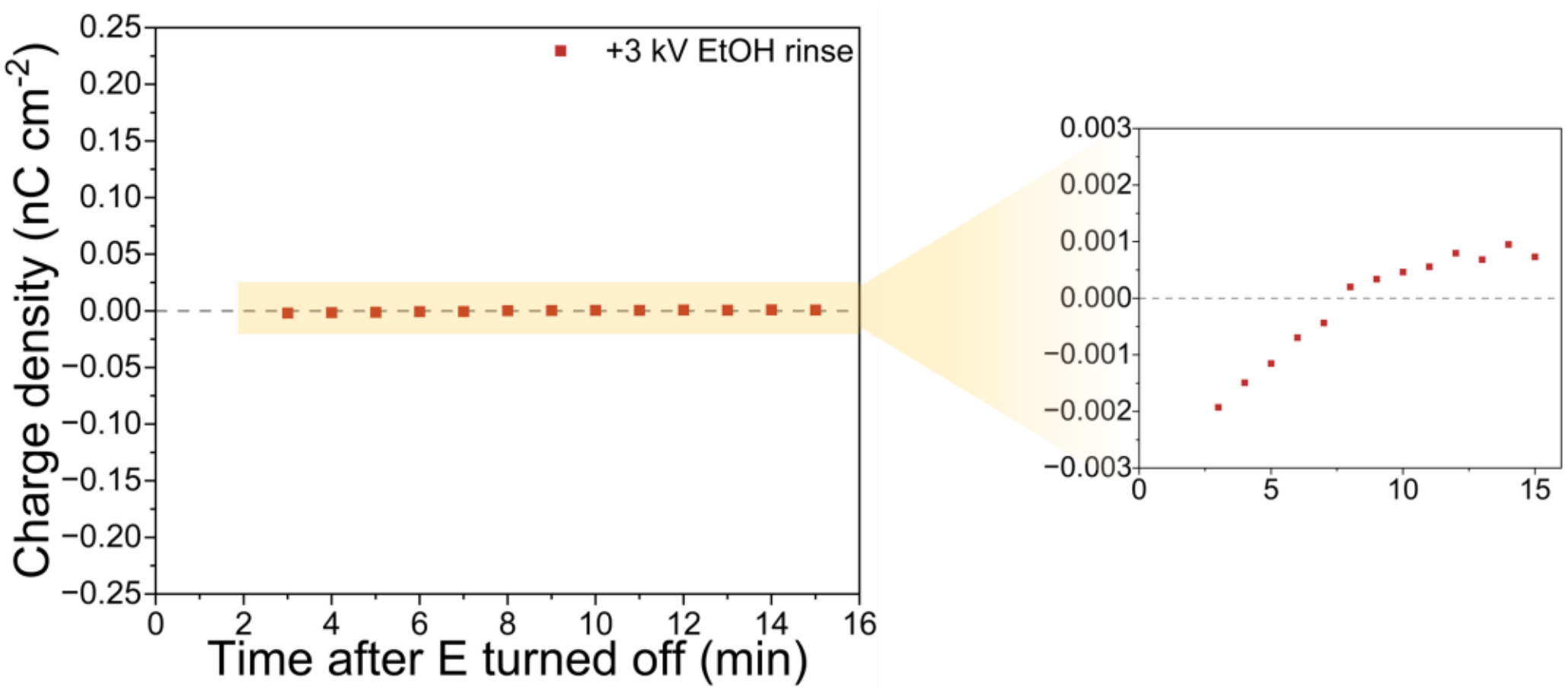

Figure S8. Charge evolution after ethanol rinsing of PS. Charge density as a function of time after PS was rinsed with EtOH before measurement. Source data are provided as a Source Data file.

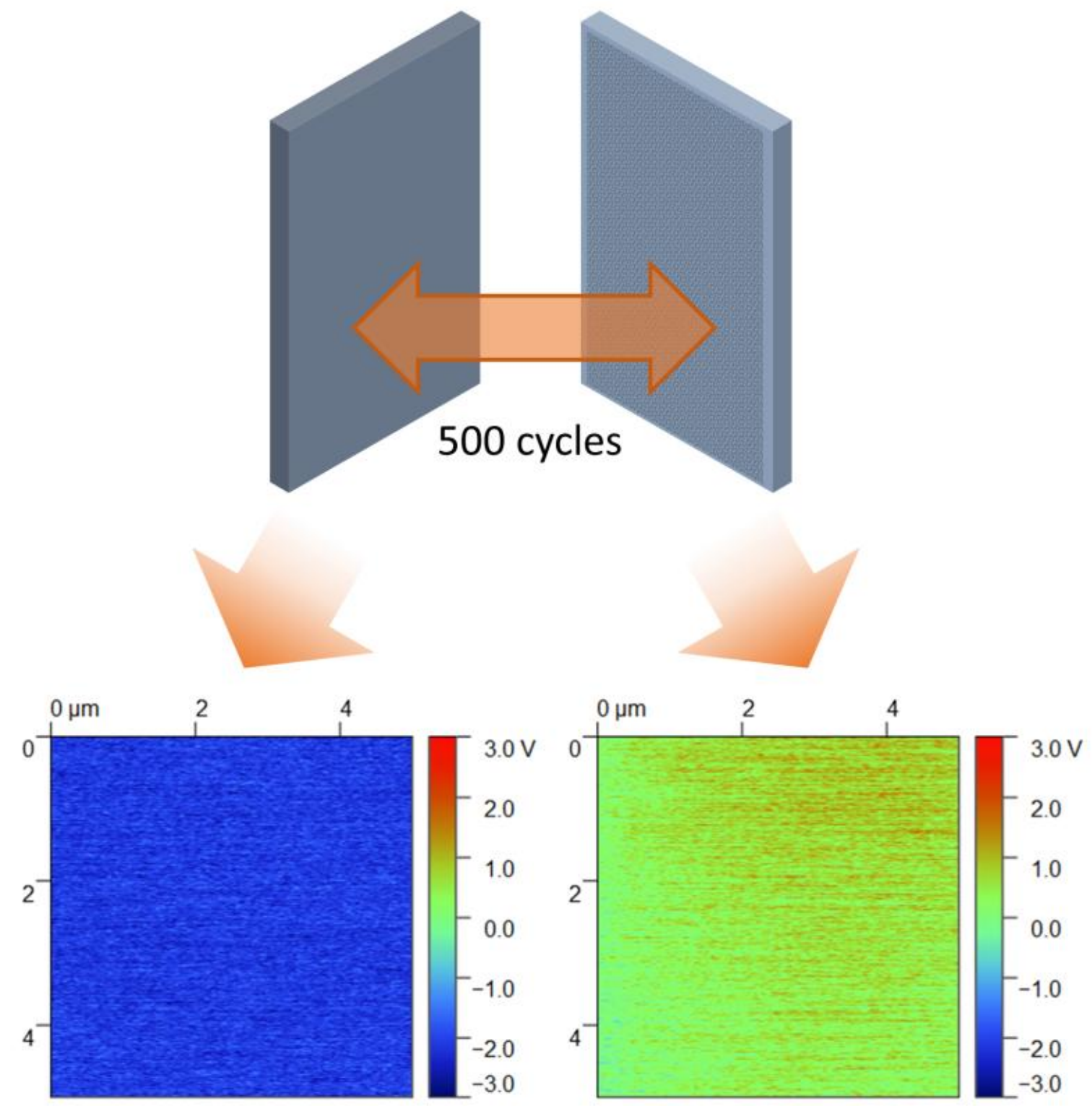

Figure S9. Surface potential of smooth and rough PS after repeated contact–separation. KPFM measurements of smooth (left) and rough (right) PS after 500 contact separation cycles without applied electric potential.

Biased flexoelectric-field simulations

Additional biased simulations were performed to examine whether an externally applied macroscopic field can reinforce or oppose the local flexoelectric field generated at a compressed asperity. The same coupled solid mechanics/electrostatics model was used as in the main flexoelectric simulations, with a 15 nm maximum compressive displacement. To approximate the experimental macroscopic field strength of approximately 30 MV m^{-1}, external biases of ±90 V were applied across the computational domain. The normal electric field and normal polarisation were then extracted at the contact interface as a function of displacement.

In the unbiased case (0 V), mechanical compression alone generated a localized flexoelectric field at the asperity. The averaged normal electric field, En, increased with displacement and reached -3.73×10^{8} V m^{-1} at 15 nm compression (Figure S10 A). When the applied field was aligned with the flexoelectric field (−90 V), the local interfacial field was reinforced, reaching -5.21×10^{8} V m^{-1} at the same displacement. Conversely, when the applied field opposed the flexoelectric field (+90 V), the local field

was partially suppressed, reaching -2.26×10^8 V m^{-1}. These results show that the externally applied field does not replace the mechanically generated flexoelectric field, but shifts its magnitude depending on field polarity.

The corresponding normal polarisation, P_n, provides the mechanistic explanation for this behaviour (Figure S10 B). The applied bias produces an immediate dielectric-polarisation offset even at zero displacement, visible as a vertical shift between the curves. Upon compression, however, all three cases show the same displacement-dependent increase associated with the strain-gradient-induced flexoelectric contribution. Thus, the total interfacial polarisation consists of two superimposed terms: a field-induced dielectric component and a mechanically induced flexoelectric component. This supports the interpretation that an external macroscopic field can penetrate the dielectric contact geometry and either reinforce or oppose the localized flexoelectric field at the asperity, depending on polarity.

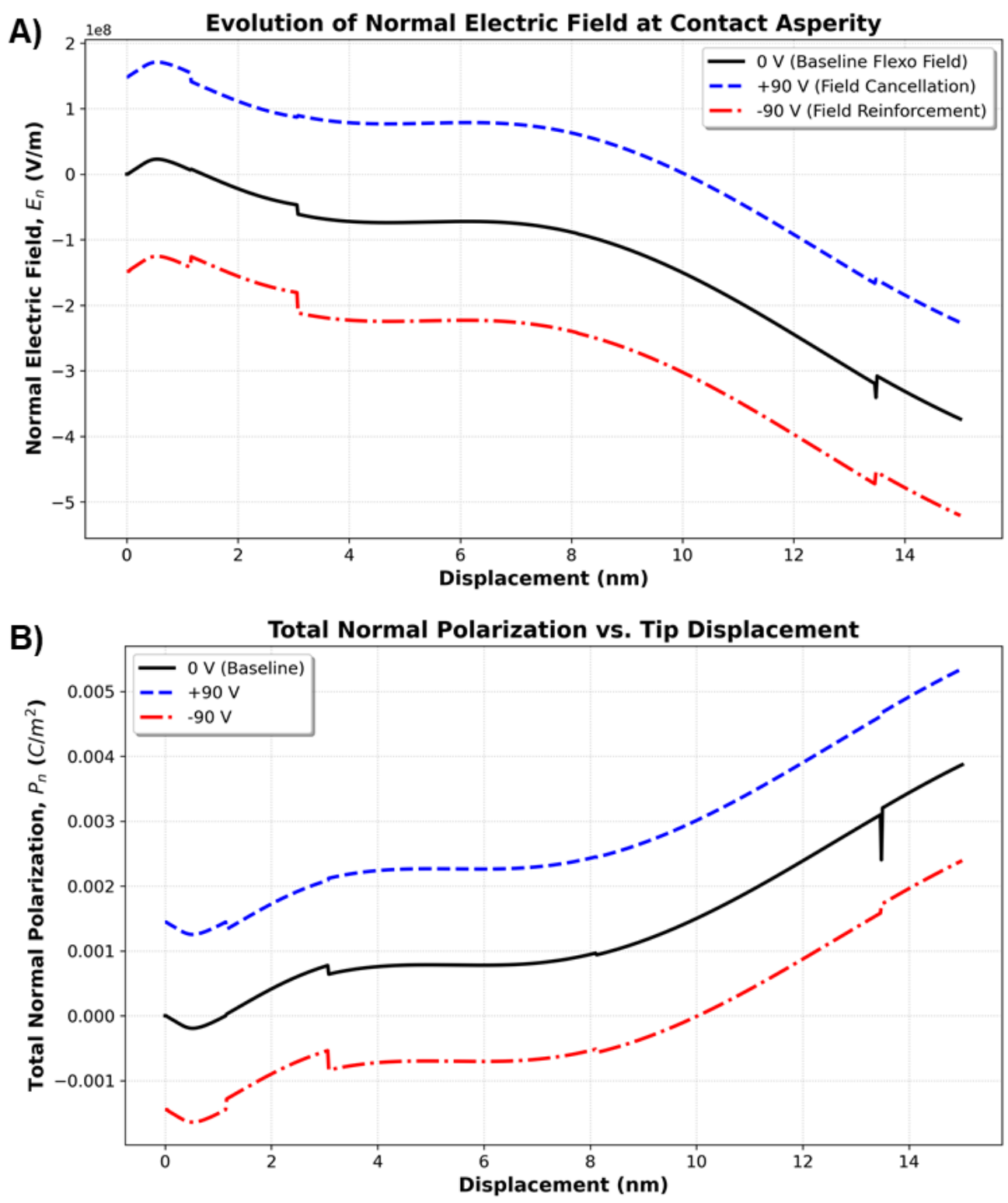

Figure S10. Effect of applied bias on the flexoelectric field at a contact asperity. Biased flexoelectric-field simulations at the contact asperity. A) Averaged normal electric field, E_n, as a function of compressive displacement under 0 V, +90 V, and −90 V bias. B) Corresponding averaged normal polarisation, P_n, showing a bias-induced offset superimposed on the displacement-dependent flexoelectric response. Source data are provided as a Source Data file.

Effect of Mechanical Properties:

Previous studies have shown that the mechanical properties directly influence the observed charge polarity and magnitude. It is shown that the softer polymers tend to gain larger surface charge density from contact separation than the hard ones.[66] This has been related to more firm contact formation (larger specific surface contacting area) or enhanced covalent bond break and material (organo-ion) transfer due to smaller cohesion energy. Moreover, by contacting chemically different polymer pairs, it has been found that the pairs with more distinct mechanical properties tend to obtain up to an order of magnitude higher surface charge density[66] as well as softer ones tend to gain negative charges, while harder – positive charges.[40] While these are exciting observations, there is no solid explanation. It is suggested that the soft polymers undergo more severe lateral deformation than the hard polymers, thus providing a different scale of friction and strain. This, in turn, should affect the flexoelectric field magnitude acting to attract specific static charge carriers.

As an additional control within a chemically similar polymer family, two silicone elastomers, Ecoflex 00-30 and Ecoflex 00-50, were compared under identical ±3 kV biased contact-separation conditions. Ecoflex 00-50 is slightly stiffer (Youngs modulus ~87 kPa) than Ecoflex 00-30 (Youngs modulus ~70 kPa) and produced slightly lower charge densities under both bias polarities. This trend is consistent with reduced deformation under the same applied force. However, even within one polymer family, changes in modulus may still be accompanied by differences in crosslink density, viscoelasticity, adhesion, and surface energy; therefore, this comparison should be treated as a supporting control rather than a strict modulus-only experiment.

Here we tested three different polymers on the effect of the electric field on surface charge density – hard polyimide (PI), moderate PS, and soft polyether block amide (PEBA). The elastic modulus and the surface roughness of the tested polymers are provided in SI Table S2. The mechanically hard PI (tensile modulus of PI is around 2.5 GPa [https://www.mit.edu/~6.777/matprops/polyimide.htm]) shows almost absent resulting static charge after relaxation (3 min), although it shows relatively high charge from polarization from 0.18 nC cm^{-2} to 0.29 nC cm^{-2}. This can be related to its mechanical

hardness. As discussed above, the harder polymers are gaining smaller charges due to a lower probability of macromolecule bond scission. This also indicates that the applied electric field is not enough to induce charge transfer. The polymer has to be prone to charge formation, but the electric field is a driving force for charge transfer to happen.

The soft elastomer PEBA, which has up to 500 times lower elastic modulus than PI, gained the largest resulting static charge density (-0.48 nC cm^{-2} and +0.42 nC cm^{-2}). This is as expected. Soft materials are easy to rupture due to their low cohesion energy, thus providing a vast amount of organo-ions that can be transferred in the presence of the electric field.

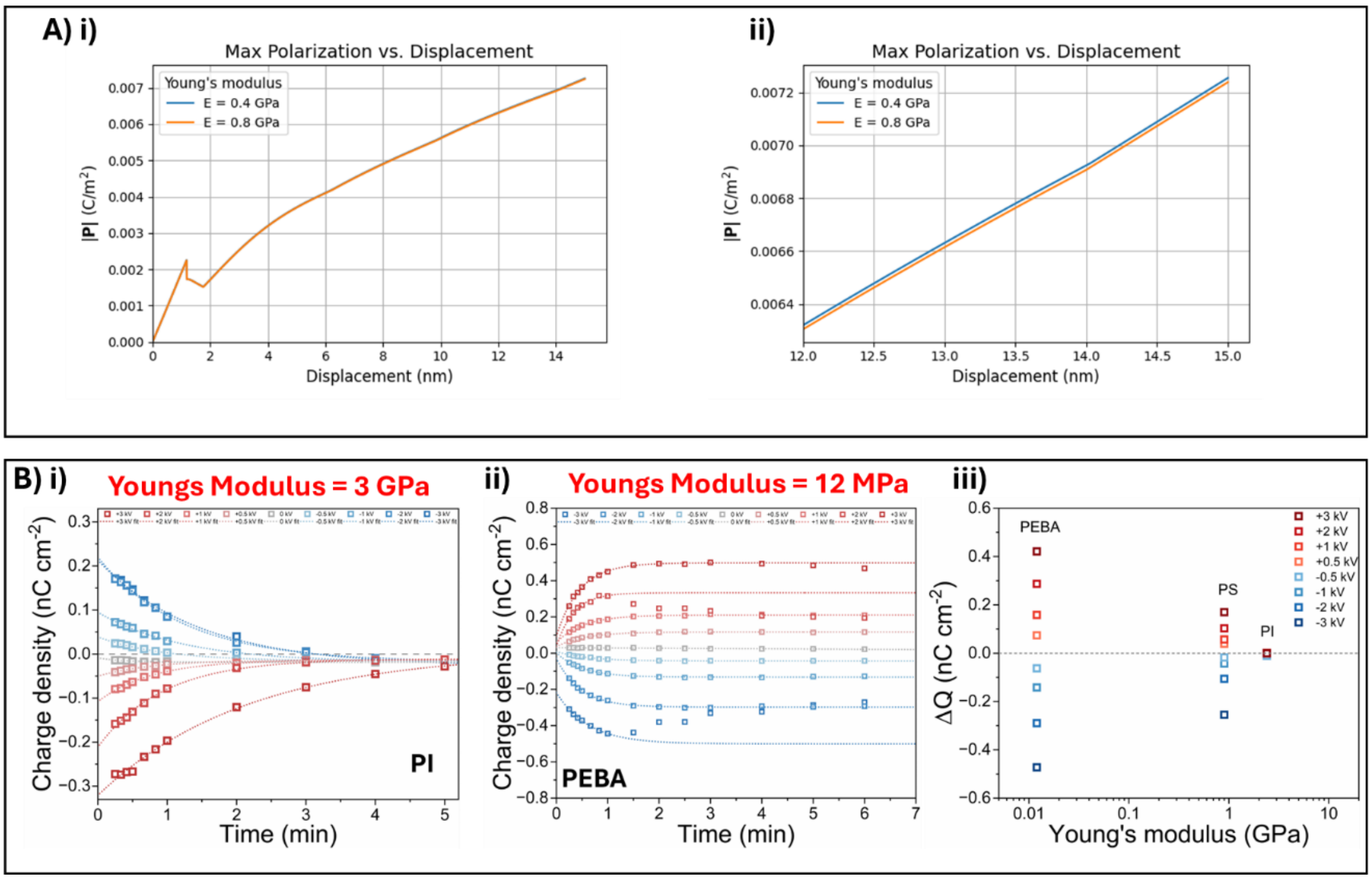

Figure S 11. Influence of Young's modulus on flexoelectric polarization and contact electrification. A) Flexoelectric simulation for PTFE with different Young's modulus; i) full range of study; and ii) zoomed in at maximum displacement. B) Effect of mechanical properties for polymers with different Young's modulus. Source data are provided as a Source Data file.

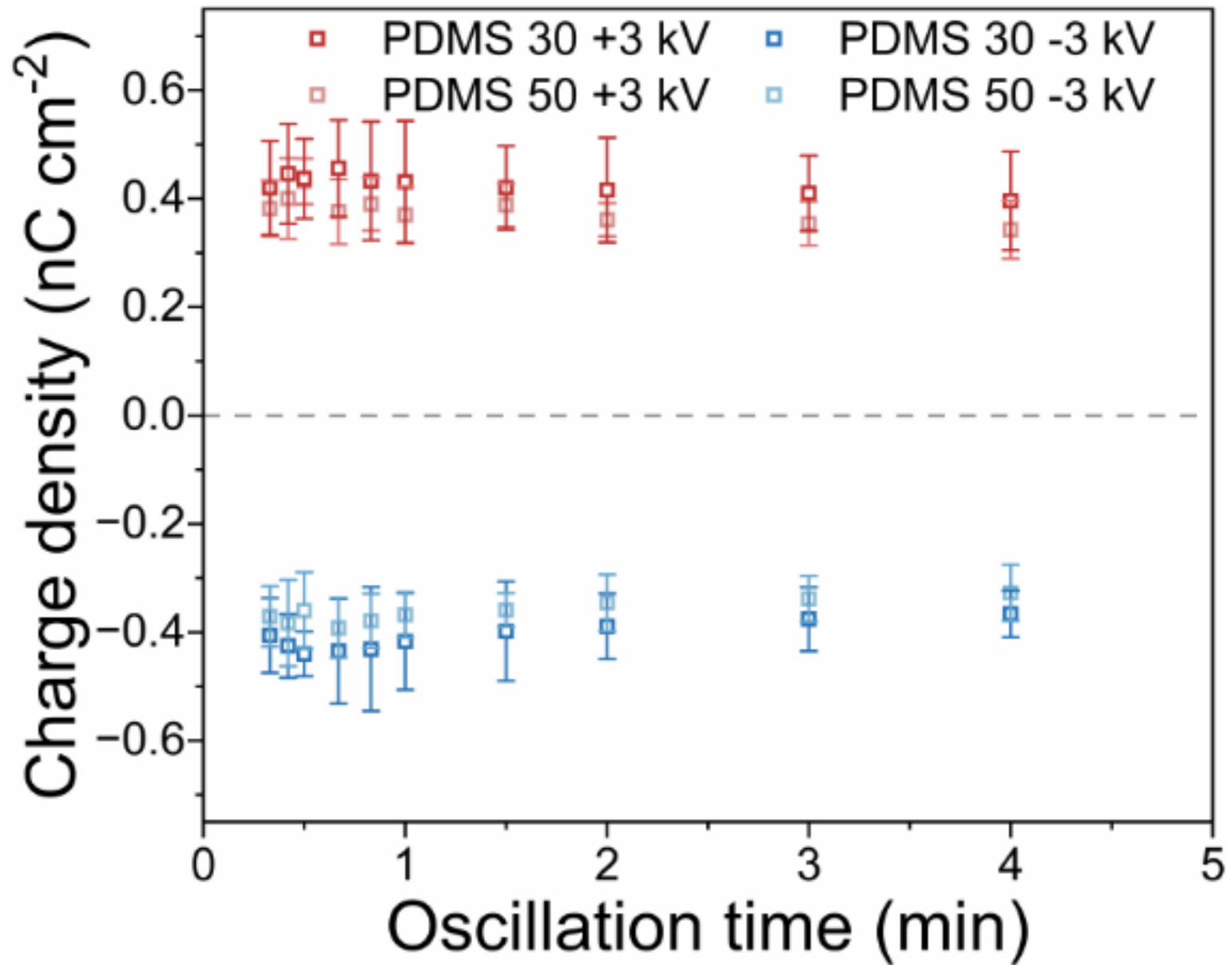

Figure S 12. Charge density evolution of Ecoflex 00-30 and Ecoflex 00-50 after biased contact–separation. Charge density comparison as a function of oscillation time for PDMS 30 and PDMS 50 elastomers after biased contact separation using ±3 kV. Data were collected from n=3 measurements, graph shows mean charge density and error bars represent SD. Source data are provided as a Source Data file.

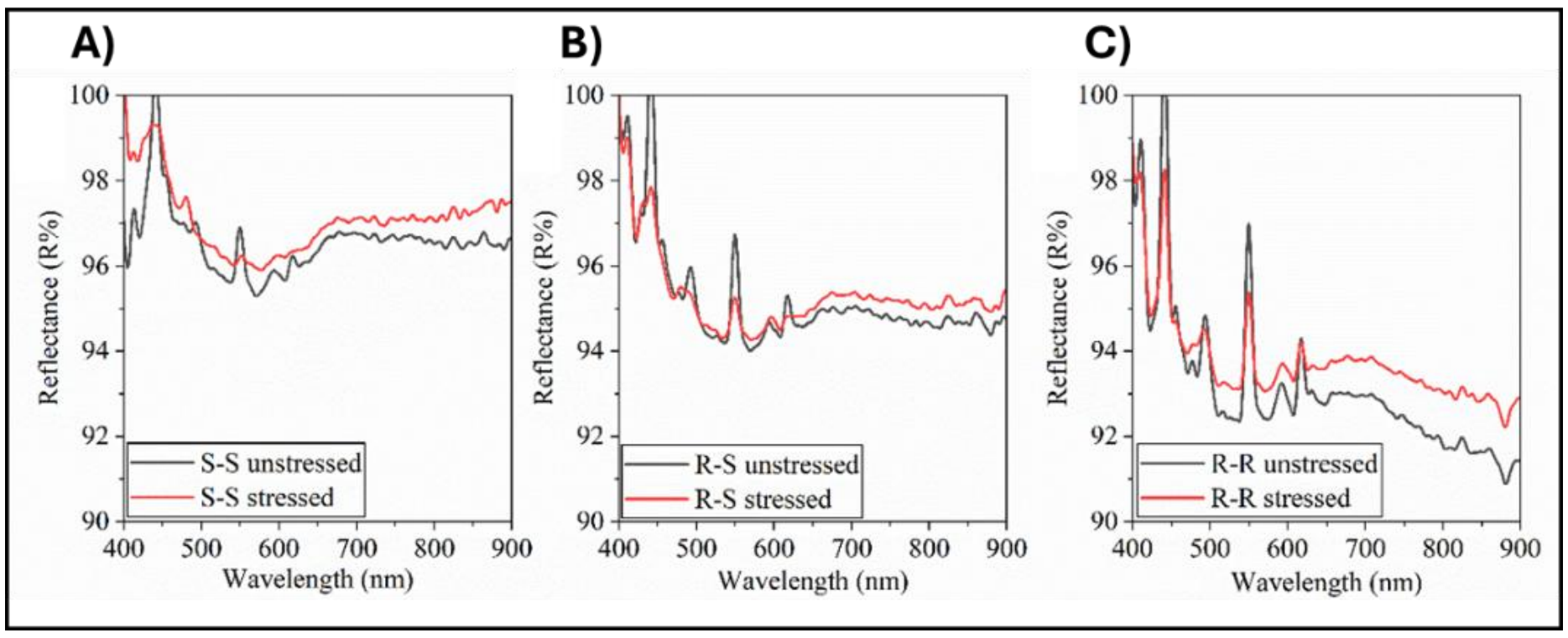

Figure S 13. Effect of surface roughness and compression on the optical absorbance of PS contacts. Optical absorbance measurement of PS films with no applied force and under compression, S denotes a smooth polymer, R denotes a rough polymer. A) smooth-smooth contact; B) rough-smooth contact; C) rough-rough contact. Source data are provided as a Source Data file.

Depolarisation

Changes in charge density over time were fitted using the ExpDec function in Origin. This gave Equation (S1):

$$Q = A_1 e^{-\frac{t}{\tau_1}} + Q_0 \qquad \text{Eq (S1)}$$

Where *Q* is charge density (nC cm^{-2}), *t* is time (min), parameter Q_0 is equilibrium charge density in the given time frame, parameter A_1 is initial deviation from equilibrium, and τ_1 is the time constant of depolarisation – measure for the speed of relaxation.

Table S 1. Depolarisation time constants for spontaneous polarisation from applied DC voltages.

Polymer	τ_1 (min)							
	-3 kv	-2 kV	-1 kV	-0.5 kV	+0.5 kV	+1 kV	+2 kV	+3 kV
Smooth PS	0.48 ± 0.01	0.51 ± 0.02	0.51 ± 0.03	0.53 ± 0.04	1.55 ± 0.15	1.06 ± 0.03	1.13 ± 0.04	1.02 ± 0.03
Rough PS	0.98 ± 0.01	1.06 ± 0.03	1.02 ± 0.04	1.24 ± 0.09	0.69 ± 0.03	0.57 ± 0.02	0.52 ± 0.01	0.45 ± 0.01
PI	1.44 ± 0.12	1.23 ± 0.06	1.22 ± 0.07	0.94 ± 0.07	0.80 ± 0.09	0.77 ± 0.04	0.93 ± 0.05	2.15 ± 0.23
PEBA	0.62 ± 0.08	0.46 ± 0.02	0.50 ± 0.03	0.59 ± 0.04	0.55 ± 0.04	0.52 ± 0.03	0.34 ± 0.08	0.45 ± 0.03
PDMS	0.18 ± 0.05	0.45 ± 0.22	0.58 ± 0.09	1.29 ± 0.27	1.22 ± 0.43	0.44 ± 0.14	0.28 ± 0.20	0.14 ± 0.08
PTFE	0.27 ± 0.06	0.21 ± 0.02	0.31 ± 0.13	5.46 ± 1.43	0.44 ± 0.05	0.22 ± 0.02	0.21 ± 0.02	0.17 ± 0.04

Material characterisation

Table S 2. Physical properties of polymer films.

<table>
<tr><th>Polymer</th><th>Thickness, µm</th><th>Surface roughness Sa</th><th>Modulus</th><th>Dielectric constant</th></tr>
<tr><td>PEBA</td><td>110</td><td>38.8 nm</td><td>12 MPa</td><td>6</td></tr>
<tr><td>PI</td><td>25</td><td>67 nm</td><td>2.41-6.10 Gpa</td><td>3.4</td></tr>
<tr><td>PS</td><td>100</td><td>32.0 nm</td><td rowspan="2">0.89-3.55 Gpa</td><td rowspan="2">2-2.7</td></tr>
<tr><td>Rough PS</td><td>100</td><td>29.0 µm</td></tr>
<tr><td>PDMS 30</td><td>100</td><td rowspan="2">1.0 nm</td><td>70 kPa</td><td rowspan="2">2.728</td></tr>
<tr><td>PDMS 50</td><td>100</td><td>87 kPa</td></tr>
<tr><td>PTFE</td><td>30</td><td>26.0 nm</td><td>0.45 GPa</td><td>2.1</td></tr>
</table>

*references; PEBA from matweb.com; PI from matweb.com and www.mit.edu/~6.777/matprops/polyimide.htm
