# Supplementary material for: Electric fields control static charge during contact-friction-separation
Source: Nat Commun. 2026 Oct 15;17:9971. doi: 10.1038/s41467-026-77733-4 (PMC13672301; doi:10.1038/s41467-026-77733-4)
Supplement: Supplementary file 2 [file 41467_2026_77733_MOESM2_ESM.pdf]

The Description of Additional Supplementary Files

Title: Supplementary Movie 1

Description: COMSOL simulation of Stress vs Displacement, Volume Contraction, Bending Deformation, Flexoelectric Polarisation “Smooth PTFE sample” (radius of curvature = 250 nm) vs Inert Substrate

Title: Supplementary Movie 2

Description: COMSOL simulation of Stress vs Displacement, Volume Contraction, Bending Deformation, Flexoelectric Polarisation “Rough PTFE sample” (radius of curvature = 50 nm) vs Inert Substrate

Title: Supplementary Movie 3

Description: COMSOL simulation of Stress vs Displacement, Volume Contraction, Bending Deformation, Flexoelectric Polarisation “Smooth PTFE sample” (radius of curvature = 250 nm) vs PTFE Substrate
