# Supplementary material for: Electric fields control static charge during contact-friction-separation
Source: Nat Commun. 2026 Oct 15;17:9971. doi: 10.1038/s41467-026-77733-4 (PMC13672301; doi:10.1038/s41467-026-77733-4)
Supplement: Supplementary file 4 [file 41467_2026_77733_MOESM7_ESM.pdf]

nature portfolio

Peer Review File

Electric Fields Control Static Charge During Contact-Friction-Separation

Corresponding Author: Dr Peter Sherrell

This file contains all reviewer reports in order by version, followed by all author rebuttals in order by version.

Version 0:

Reviewer comments:

Reviewer #1

(Remarks to the Author)
This manuscript explored the intriguing possibility of modulating static charge accumulation during contact electrification via external electric fields. While the fundamental mechanisms governing contact electrification remain a topic of significant interest to the materials science community, the current submission falls short of clarity regarding the current related foundational research context, the mechanisms explanation of this work, and the experimental description. Consequently, the manuscript is not suitable for publication in its present form and requires a comprehensive overhaul.

1. There are some inexplicit statements, such as “These results show conclusively for the first time that electric fields can be used to control and invert the surface charge obtained on a surface via CE, ”. However, there are previous work seems already reported such perspective:
The authors’ assertion that these results 'show conclusively for the first time that electric fields can be used to control and invert the surface charge obtained via CE' is empirically overreached and seems to ignore the following established literature.
(1) Dai et. al found that the external electric field essentially changes the local work function of surfaces and consequently modulates contact electrification. (DOI: 10.1021/acs.jpcc.2c00497).
(2) Zhou et al. (DOI: 10.1021/nl404819w) used an AFM-based CE setup (Pt-coated tip contacting/rubbing a dielectric thin film) while applying an external electric field (bias). They showed the applied field shifts the effective barrier/energetics for electron transfer (and back-tunneling) at the interface, so the net transferred charge can be pushed through zero to the opposite sign.
(3) Lee, Kim, and co-workers (DOI:10.1002/adfm.201505088) showed that by poling a ferroelectric polymer (e.g., P(VDF-TrFE)) with positive vs. negative bias and then performing triboelectric contact, both the amount and direction of triboelectric charge transfer can be controlled, i.e., the triboelectric response depends on polarization state, enabling sign control/inversion in practice.
The manuscript fails to acknowledge these precedents or, more importantly, to differentiate the current findings from existing models. To claim such a 'first' in a high-impact journal, the authors must provide a rigorous comparative analysis that highlights a fundamentally new physical insight. Without this, the novelty of the work is significantly overstated.

2. In the second paragraph of the main text, the authors offer an overly reductive summary of triboelectrification mechanisms, summarily dismissing electron transfer as a governing factor in polymers. This conclusion is supported solely by a single self-citation, an approach that ignores a vast body of literature discussing the nuanced interplay between electron, ion, and mass transfer. This insular citation strategy undermines the credibility of the research background within the broader scientific discourse.

3. The data presented in Figure 1B is critically underspecified, it is unclear whether the reported "time" represents the contact duration between Polymer A and Polymer P, or the suspension time of the charged Polymer A on the electrometer. Furthermore, it is unclear why the surface charge density changes over time, is this serve as evidence of material transfer?

4. In Figure S2, it is helpful to provide the corresponding SEM images to support the comparison of material surfaces after different high-voltage and contact treatments. In addition, the possibility that the applied high voltage induces surface roughening or damage due to electrical breakdown should be carefully considered.

5. The manuscript states that the increase in RMS surface roughness after contact is consistent with material or adsorbate transfer associated with contact electrification (Figures S2 and S3), and this observation is extended across multiple dielectric polymers. However, it is not fully clear whether surface roughness alone can be taken as direct evidence of material transfer responsible for the observed changes in charge density. In particular, the experiments are conducted under the application of a relatively high external voltage (on the order of several kilovolts), whereas negligible charge density is reported in the absence of external bias. This raises an important question as to whether the observed surface roughening arises intrinsically from contact electrification–induced material transfer, or instead from voltage-induced effects such as dielectric breakdown, local discharge, or field-driven surface damage. The authors are encouraged to clarify how material transfer due specifically to contact electrification can be distinguished from surface modification induced by the applied high electric field, and to discuss whether additional control experiments or morphological evidence (e.g., SEM) are available to rule out voltage-induced artifacts.

6. Figure 1d presents a highly simplified schematic that does not sufficiently convey the underlying physical processes. In particular, it is difficult to understand how charge generation, distribution, or transfer occurs under positive and negative external DC bias, as well as in the absence of an applied voltage. A more detailed schematic explicitly illustrating the charge configurations and transfer pathways in each case would greatly improve clarity. In addition, the manuscript describes the process as “spontaneous,” yet the experiments are performed under an applied DC electric field. The authors are encouraged to clarify the appropriateness of this terminology and to distinguish clearly between truly spontaneous charge transfer and field-assisted or field-driven processes.

7. In the simulation section, PTFE is selected as the model polymer on the basis of the availability of its flexoelectric coefficient. However, the manuscript lacks a substantive discussion linking the theoretical simulations to the experimental observations. The authors are encouraged to more explicitly compare and contextualize the simulation results with the corresponding experimental findings, and to discuss the extent to which the PTFE-based model captures the behavior of the materials investigated experimentally. Strengthening the correlation between theory and experiment would significantly improve the rigor and interpretability of the work.

Reviewer #2

(Remarks to the Author)
In this work, the authors investigate the driving forces behind contact electrification between dielectric polymers. The manuscript claims that electric fields generated by the flexoelectric effect drive charge separation. The authors demonstrate that applying an external direct current electric field during the contact and separation of identical polymers can dictate the magnitude and polarity of the resulting static charge. The authors couple these macroscopic external field experiments with computational simulations of flexoelectric fields induced by surface asperities. The manuscript reports a clever experimental setup using identical materials under external fields to isolate polarization effects. The main concern around the manuscript is the logical leap required to equate macroscopic external direct current fields with highly localized flexoelectric fields. The experimental data do not conclusively isolate flexoelectricity from other contact induced charging mechanisms. Therefore, I recommend this paper to be accepted after major revision.
- The authors claim to provide conclusive evidence of flexoelectricity as the driving force for contact electrification. This builds heavily on recent proposals by Marks and Olson. Marks and Olson correlated flexoelectricity with triboelectricity using atomic force microscopy in Nano Letters in 2022. The authors must better distinguish their macroscopic external field approach from this prior research paper. The authors should discuss and add exactly what experimental novelty is introduced beyond the theoretical framework already established.
- In the manuscript, an external field applies a uniform macroscopic polarization across the entire bulk polymer. Flexoelectric fields are generated by highly localized strain gradients at nanoscale asperities. Showing that a massive external field directs charge transfer does not explicitly prove that native flexoelectric fields do the same under normal conditions.
- The manuscript dismisses electron transfer by stating band structure alignment is only relevant for electron transfer. The authors assume organo ions or adsorbates are the primary charge carriers. They do not explain how a macroscopic electric field selectively templates the transfer of massive organo ions rather than electrons.The authors should explain mechanistically to support this claim.
- The main contact electrification experiments use polystyrene. The simulations in Figure 2B use polytetrafluoroethylene. The authors justify this by stating nearly all polymers show a flexoelectric coefficient of the same sign. Comparing experimental data of one material with simulations of another introduces unnecessary quantitative inconsistencies. The authors should simulate polystyrene or provide corresponding rough and smooth experimental data for polytetrafluoroethylene.
- The procedure to extract the stable surface charge is not entirely convincing. The authors fit an exponential decay to separate the spontaneous polarization from the stable static charge in Figure S1. It is difficult to rule out the formation of long lived electrets or deep trapped charges caused by the high external voltage. It would clarify this point by showing control experiment to prove that the residual charge is genuinely identical in nature to standard contact electrification.
- The discussion of compressive modulus in Figure S4 needs stronger controls. Comparing polymers with widely different Youngs moduli inherently adds multiple confounding factors. These polymers also differ in surface energy and cohesive energy. Yet the authors attribute the change in charge exclusively to increased flexoelectric polarization from larger deformation. To support this conclusion, they should experimentally vary modulus while keeping the other chemical properties effectively constant.
- The manuscript contains several grammatical errors and typos that hinder clarity. The title uses incorrect subject verb agreement with the phrase Electric Fields Controls. The word performed is misspelled in the caption of Figure 1.

Reviewer #3

(Remarks to the Author)
The paper by Sutka et al about the influence of applied electric fields on the charge separation process is interesting but not very surprising. The topic is of general interest and the study add a useful contribution to this topic but I am not convinced if it should be published in Nature. I note that there is an earlier theory study [see 2020 EPL (Europhysics Letters) 129(1):10006] about the influence of surface roughness on the flexoelectric effect so that part of the paper is not very novel. But I liked the video presentations of the flexoelectric effect.

Reviewer #4

(Remarks to the Author)
The manuscript reports that externally applied electric fields can control the polarity and magnitude of surface charge generated during contact electrification between polymers, and proposes that flexoelectric fields generated during mechanical contact are the underlying driving force for this phenomenon. While the topic is interesting and potentially relevant to the fundamental understanding of triboelectrification, the current manuscript does not provide sufficiently rigorous experimental evidence to support the proposed mechanism. In particular, the relationship between externally applied electric fields and flexoelectric polarization is not convincingly demonstrated, and several alternative explanations remain plausible. Therefore, this reviewer does not recommend publication of the manuscript in its current form. The following points should be carefully addressed.
Comment 1
The authors claim that an externally applied electric field can control the polarity of surface charge generated during contact electrification. However, the physical mechanism underlying this phenomenon is not clearly established. Although the authors introduce a protective polymer layer to prevent direct charge injection from the metal electrode, it is well known that strong electric fields can induce dipole alignment or partial poling in dielectric polymers. Such electric-field-induced polarization can produce transient surface charge distributions even without triboelectric processes. Therefore, it remains unclear whether the observed charge inversion truly originates from contact electrification or simply from electric-field-induced polarization effects.
In particular, several key questions remain unresolved:
- Electric field penetration through the protective polymer layer
The manuscript assumes that the protective polymer layer eliminates direct electrical influence from the metal electrode. However, depending on the dielectric constant and thickness of the protective layer, a substantial electric field may still penetrate into the upper polymer layer. Under sufficiently high voltages (±3 kV in this work), this field could induce polarization or charge trapping in the polymer. Quantitative analysis of the electric field distribution across the multilayer structure is therefore necessary.
- Stability of the measured surface charge
If the observed surface charge originates from electric-field-induced polarization or electret-like charge trapping, the temporal stability of the charge becomes an important indicator. The manuscript reports charge decay over several minutes, but it is not clear whether the remaining charge persists over longer timescales (e.g. hours or days), which is commonly reported for triboelectric charges.
- Role of repeated friction cycles
The authors perform 500 contact–separation cycles prior to measuring the charge using non-contact oscillation. The rationale for this procedure is not sufficiently justified. If the effect is primarily driven by the applied electric field, it would be important to show control experiments in which the polymer is simply exposed to the electric field without repeated friction. Such experiments would help distinguish between triboelectric charging and electric-field-induced charge accumulation.

Comment 2
The manuscript attempts to correlate the externally applied electric field effect with flexoelectricity induced by surface roughness. While this idea is intriguing, the experimental evidence presented here is insufficient to establish such a relationship. Flexoelectric polarization arises from strain gradients at the nanoscale, and the direction and magnitude of the polarization strongly depend on the local mechanical deformation field. In contrast, the externally applied electric field in this work is a macroscopic uniform field. Directly equating these two effects appears to be an oversimplification. The authors suggest that flexoelectric fields generated at rough surfaces may either enhance or cancel the polarization induced by the external DC electric field. However, this hypothesis is not supported by rigorous experimental verification. To substantiate this claim, more direct measurements of flexoelectric responses are required. For example:
- Atomic force microscopy-based measurements (e.g., piezoresponse or Kelvin probe force microscopy) could be used to detect local polarization or surface potential changes associated with flexoelectric deformation.
- Direct measurements of surface potential distributions could help verify whether strain-induced polarization fields are indeed present during contact.
- Quantitative comparison between the estimated flexoelectric field and the externally applied electric field would also be necessary.
Moreover, although previous studies have suggested that flexoelectricity may contribute to triboelectric charge generation, demonstrating that the external electric field and flexoelectric fields can cancel or reinforce each other requires much stronger theoretical modeling and experimental validation than currently provided. Therefore, the proposed interpretation linking the experimental observations to flexoelectricity remains speculative.

Version 1:

Reviewer comments:

Reviewer #1

(Remarks to the Author)

Reviewer #2

(Remarks to the Author)
The authors have provided detailed comment from last revision, but there are some concerns left.
- The abstract still mentions "for the first time" of field-induced inversion of CE charge. Field-driven sign inversion has been shown before in other configurations. The "first" should be scoped explicitly in the abstract to insulating polymer-polymer contacts, otherwise it can be read as overstated.
- I have concerns that FEM result might be biased. A linear coupled electrostatics-mechanics model will superimpose an applied bias on a strain-gradient field by construction. The simulation therefore confirms an assumption rather than testing the mechanism. The authors should remove any claim that this simulation proves flexoelectric drive, and state plainly that the flexoelectric link is a supported hypothesis.
- The PS-versus-PTFE mismatch was justified rather than resolved. PS apparently has no reported flexoelectric coefficient, which is a real constraint, and preparing PTFE with controlled, reproducible roughness is genuinely difficult, so neither requested route was straightforward. Even so, Fig. 2B still uses PTFE simulations to rationalize a PS measurement in Fig. 2A. The smooth-only PTFE data in Fig. S5 confirm field-dependent charging but do not provide the rough/smooth comparison that was asked for. As a result, the simulation supports the sign and the qualitative trend, not a quantitative match to the PS system. The authors should relabel and rephrase Fig. 2B and its discussion
- The field-magnitude comparison is convenient and underspecified. The external field is quoted as about 3 x 10^7 V m-1, the un-divided value, whereas Fig. S1 shows only about 2.1 x 10^7 V m-1 reaching the PS after the potential divides across the stack. The flexoelectric "surface-averaged" fields of 1.9 to 4.3 x 10^7 V m-1 are averaged over an undefined region, while peak local fields are 50 to 100 times larger. Whether the fields are "comparable" depends entirely on the averaging domain, which must be specified, and the penetrated field should be used.
- The Ecoflex 00-30 versus 00-50 comparison keeps the chemistry roughly constant, but difference in stiffness seems to be small. The authors should report the effect size and its uncertainty rather than a qualitative "slightly lower"

Reviewer #3

(Remarks to the Author)
I have read the authors response to all the reviewers reports!
It is OK with me if the present version of the paper is published in Nature
although I still do not find the paper more important than most other papers published on this topic in different journals!

Reviewer #4

(Remarks to the Author)
The authors have made substantial improvements during the revision process, particularly in clarifying the mechanistic aspects underlying the reviewer’s previous concerns. While it is already well established that internal polarization in ferroelectric polymers can modulate contact electrification, this work extends the concept beyond conventional ferroelectric polymers to more general polymer systems. The authors’ discussion linking this behavior to flexoelectricity is scientifically interesting and provides a meaningful perspective on the observed phenomena. This reviewer therefore recommends publication in its current form.

Version 2:

Reviewer comments:

Reviewer #2

(Remarks to the Author)
The authors have answered every comment in detail from last revision. I recommend the manuscript to be published as is.

Open Access This Peer Review File is licensed under a Creative Commons Attribution 4.0 International License, which permits use, sharing, adaptation, distribution and reproduction in any medium or format, as long as you give appropriate credit to the original author(s) and the source, provide a link to the Creative Commons license, and indicate if changes were made.
In cases where reviewers are anonymous, credit should be given to 'Anonymous Referee' and the source.
The images or other third party material in this Peer Review File are included in the article's Creative Commons license, unless indicated otherwise in a credit line to the material. If material is not included in the article's Creative Commons license and your intended use is not permitted by statutory regulation or exceeds the permitted use, you will need to obtain permission directly from the copyright holder.
To view a copy of this license, visit https://creativecommons.org/licenses/by/4.0/

REVIEWER COMMENTS

Reviewer #1 (Remarks to the Author):

This manuscript explored the intriguing possibility of modulating static charge accumulation during contact electrification via external electric fields. While the fundamental mechanisms governing contact electrification remain a topic of significant interest to the materials science community, the current submission falls short of clarity regarding the current related foundational research context, the mechanisms explanation of this work, and the experimental description. Consequently, the manuscript is not suitable for publication in its present form and requires a comprehensive overhaul.

Answer: We thank the reviewer for their time and diligence, and appreciate the positive evaluation of the premise of the manuscript and work. In our revision comments below we have worked to address the clarity, experimental description, and mechanistic discussion.

1. There are some inexplicit statements, such as “These results show conclusively for the first time that electric fields can be used to control and invert the surface charge obtained on a surface via CE, ”. However, there are previous work seems already reported such perspective:
The authors’ assertion that these results 'show conclusively for the first time that electric fields can be used to control and invert the surface charge obtained via CE' is empirically overreached and seems to ignore the following established literature.
(1) Dai et. al found that the external electric field essentially changes the local work function of surfaces and consequently modulates contact electrification. (DOI: 10.1021/acs.jpcc.2c00497).

Answer: We thank the reviewer for pointing out prior work showing that external electric fields can influence contact electrification through changes in local work function (e.g., Dai et al.).

We agree that such effects are relevant. However, it should be noted that the work referenced above is based on conductive carbon nanotube systems, where charge redistribution and work function shifts can be directly coupled to charge transport. In contrast, our work in the present study focuses on dielectric (highly resistive) polymeric materials, which are electrically insulating, and where additional mechanisms such as interfacial bond scission and material transfer are expected to play a more significant role.

Here, we direct readers to a seminal perspective from Lacks and Shinbrot (10.1038/s41570-019-0115-1) to differentiate mechanisms between conductive, semi-conductive, and insulating materials.

To further confirm and demonstrate material transfer, new experiments were perfoemd with ATR-FTIR on PS surfaces after contact with PDMS under −3 kV, 0 kV, and +3 kV conditions (Fig. S4). As shown in Fig. S4 A, a feature near 1260 cm^{-1}, corresponding to the Si–CH_3 deformation mode of PDMS, appears after contact with PDMS but is absent in PS–PS control experiments. This indicates that PDMS-derived material is transferred to the PS surface during contact.

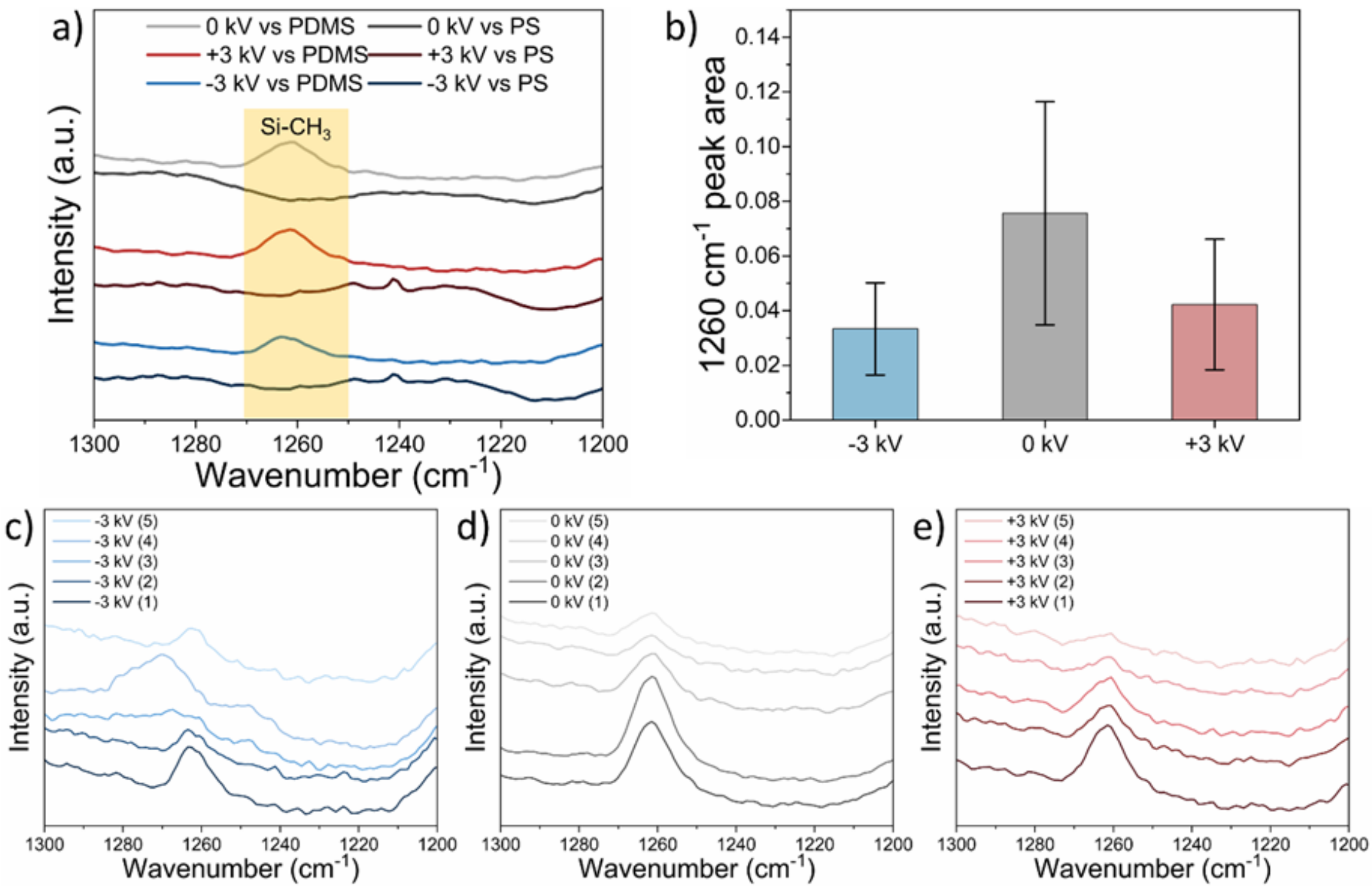

Figure S4. A) ATR-FTIR spectra (1300-1200 cm^{-1}) comparison for PS contacted either against another PS or PDMS with (±3 kV) and without (0 kV) electric potential applied; B) calculated peak area for the characteristic PDMS peak at 1260 cm^{-1}; C) ATR-FTIR measurements at five different spots for PS contacted against PDMS using -3 kV potential; D) ATR-FTIR measurements at five different spots for PS contacted against PDMS without potential E) ATR-FTIR measurements at five different spots for PS contacted against PDMS using +3 kV potential.

We then quantified this band using five independent measurements for each condition (Fig. S4 B). The average values for −3 kV, 0 kV, and +3 kV are similar and the standard deviations overlap, so within the sensitivity of ATR-FTIR no clear dependence on the applied electric potential can be resolved. It is also important that the 1260 cm^{-1} signal is relatively weak compared to the main PS bands, and much lower than what would be

expected for a continuous PDMS layer. This suggests that the transferred material forms a very thin and likely non-uniform layer. The variability between individual spectra (Fig. S4 C-E) supports this, however this makes precise quantification of small differences in transferred amount difficult.

To address these points, we have also modified the main text to read:

"Material transfer was also probed independently by ATR-FTIR in PS-PDMS contacts, where a weak PDMS-related Si-CH_3 band near 1260 cm^{-1} appeared on PS after contact but was absent in PS-PS controls, consistent with a thin/non-uniform transferred layer (Figure S4)."

(2) Zhou et al. (DOI: 10.1021/nl404819w) used an AFM-based CE setup (Pt-coated tip contacting/rubbing a dielectric thin film) while applying an external electric field (bias). They showed the applied field shifts the effective barrier/energetics for electron transfer (and back-tunneling) at the interface, so the net transferred charge can be pushed through zero to the opposite sign.

Answer: We thank the reviewer for pointing out the work by Zhou et al. While that study demonstrates field-controlled charge transfer, it is based on a metal–dielectric configuration. In contrast, the present work focuses on contact between polymeric materials, primarily identical pairs (e.g., PS–PS, PDMS–PDMS), specifically to avoid interfaces where predefined electronic energy alignment dominates the outcome. Here, we appreciate the reviewers comments on clarity in methodology, and have ensured this clarity is improved in the revised text.

Under these conditions, the mechanism cannot be directly described by metal–dielectric electron transfer models. Instead, our results indicate that interfacial processes intrinsic to polymer–polymer contact must be considered. This is supported by the PS–PDMS experiments (Fig. S4, shown above), which were included to explicitly demonstrate that material transfer occurs during contact. Therefore, while the applied electric field can influence the final charge state, its role in polymer systems should be interpreted in the context of these interfacial processes rather than as a direct analogue to the mechanism proposed for metal–dielectric contacts.

We have updated the text to read:

"Electric fields have previously been shown to influence CE through work-function modulation in conductive systems, bias-controlled charge transfer in metal-dielectric contacts, and remanent polarisation in ferroelectric polymers. In contrast, this study examines non-ferroelectric insulating polymer contacts, including identical polymer pairs, where conductive charge redistribution, metal-dielectric energy alignment, and stable ferroelectric polarisation are absent."

(3) Lee, Kim, and co-workers (DOI:10.1002/adfm.201505088) showed that by poling a ferroelectric polymer (e.g., P(VDF-TrFE)) with positive vs. negative bias and then performing triboelectric contact, both the amount and direction of triboelectric charge transfer can be controlled, i.e., the triboelectric response depends on polarization state, enabling sign control/inversion in practice.

The manuscript fails to acknowledge these precedents or, more importantly, to differentiate the current findings from existing models. To claim such a 'first' in a high-impact journal, the authors must provide a rigorous comparative analysis that highlights a fundamentally new physical insight. Without this, the novelty of the work is significantly overstated.

Answer: We thank the reviewer for pointing out the work by Lee, Kim and co-workers. In that study, control over the magnitude and polarity of triboelectric charge transfer is achieved through ferroelectric polarization of P(VDF-TrFE), where the surface is pre-poled and retains a stable polarization charge that directly influences subsequent contact electrification. In contrast, the present work does not involve ferroelectric materials or pre-polarized surfaces. The systems studied here are non-ferroelectric polymers, where such built-in polarization states are absent. Therefore, the mechanism reported by Lee et al., which relies on ferroelectric polarization as a persistent internal field, is not directly applicable to the present case. We note that ferroelectric polarization effects in polymer systems are well established, including in our own prior work (10.1021/acsaem.3c01196; 10.1021/acsaem.9b00836; 10.1039/c8ee00550h), where poling-induced polarization was shown to directly influence electrical output. Electric output measured from ferroelectric polymer-based triboelectric generator devices is governed by aligned ferroelectric dipoles, which build up the permanent electric fields that can be measured even in non-contact mode when electrodes are oscillated.

We have adapted the introduction to mention the work of Lee *et al*,

“Electric fields have previously been shown to influence CE through work-function modulation in conductive systems, bias-controlled charge transfer in metal-dielectric contacts, and remanent polarisation in ferroelectric polymers. In contrast, this study examines non-ferroelectric insulating polymer contacts, including identical polymer pairs, where conductive charge redistribution, metal-dielectric energy alignment, and stable ferroelectric polarisation are absent.”

2. In the second paragraph of the main text, the authors offer an overly reductive summary of triboelectrification mechanisms, summarily dismissing electron transfer as a governing factor in polymers. This conclusion is supported solely by a single self-citation, an approach that ignores a vast body of literature discussing the nuanced

interplay between electron, ion, and mass transfer. This insular citation strategy undermines the credibility of the research background within the broader scientific discourse.

Answer: Electron transfer is not the governor mechanism in polymers as considered in vast number of papers related to triboelectric nanogenerators. Indeed, electron transfer, ion transfer and material (organoion) transfer are the mechanisms proposed in polymer contact electrification [D.J. Lacks, T. Shinbrot, Nat. Rev. Chem. 2019, 3, 465]. While electron transfer is widely accepted in compartments of the TENG community, the community investigating the contact electrification mechanisms of polymer insulators opposes electron transfer between polymers with no free electrons, acceptor, or donor states. Even defects, considered as sites for electron transfer, are insufficient, with a -5 orders of magnitude difference between the defect density and the charge density [Waitukaitis et al., Phys. Rev. Lett. 2014, 112, 218001].

We apologise for the appearance of self-citation – and have expanded the introduction & reference pool for this section:
"Recent work has highlighted the contribution of charged molecular fragments or organo-ions (due to bond scission or adsorbate ionisation) to observed contact electrification,[7-9] although electron transfer, ion transfer, adsorbate-mediated charging, and material transfer have all been proposed as possible charge-transfer pathways in contact electrification.[6,10,11]"

and

"Band structure alignment can be relevant for conductive or metal-dielectric interfaces, where charge redistribution and electronic energy alignment are directly coupled to charge transport.[19,27] However, for polymer-polymer contacts between electrical insulators, the absence of free carriers and well-defined donor/acceptor states makes purely electronic descriptions less directly applicable.[6,7,10] Accordingly, band structure alignment can be neglected as driving force for polymer insulator-based systems."

3. The data presented in Figure 1B is critically underspecified, it is unclear whether the reported "time" represents the contact duration between Polymer A and Polymer P, or the suspension time of the charged Polymer A on the electrometer. Furthermore, it is unclear why the surface charge density changes over time, is this serve as evidence of material transfer?

Answer: Thank you for this important comment. We agree that the original description of Figure 1B was not sufficiently clear. In this figure, "time" refers to the non-contact oscillation time after the external electric potential has been switched off. During this period, the sample undergoes repeated non-contact oscillation cycles, and the surface

charge density is monitored as a function of time after removal of the bias. Accordingly, no “new” charge is generated after electrical potential is turned off. We have now specified in graphs that this is oscillation time.

The change in surface charge density over time arises from the competition between two processes (Schematic image in Figure 1 D) with different characteristic timescales and resulting polarity. When the identical polymer films are contact-separated under an applied electric field, we can expect not only the triboelectric charge transfer but also the material polarisation. In polarisation the opposite charge to electric field polarity is accumulated nearby surface, while the same sign of triboelectric charge is transferred to other contacting surface, leaving charge on the surface which is with opposite polarity to accumulated charge from polarisation. The faster process is relaxation of the field-induced polarization state. This contribution initially dominates the signal and gives a charge polarity opposite to the previously applied bias, i.e., a negative charge density after +3 kV treatment and a positive charge density after −3 kV treatment. At the same time, a slower but more persistent contribution is associated with electric-field-assisted chain scission and subsequent material transfer at the polymer–polymer interface. This transferred material carries its own characteristic polarity, which in our case corresponds to a positive charge density (+3 kV potential) and vice versa. As a result, the short-time response is initially governed by the transient polarization effect, but as this component relaxes, the contribution from transferred material progressively becomes dominant. Therefore, the time dependence reflects a temporal crossover between a fast reversible electrostatic process and a slower interfacial material transfer process.

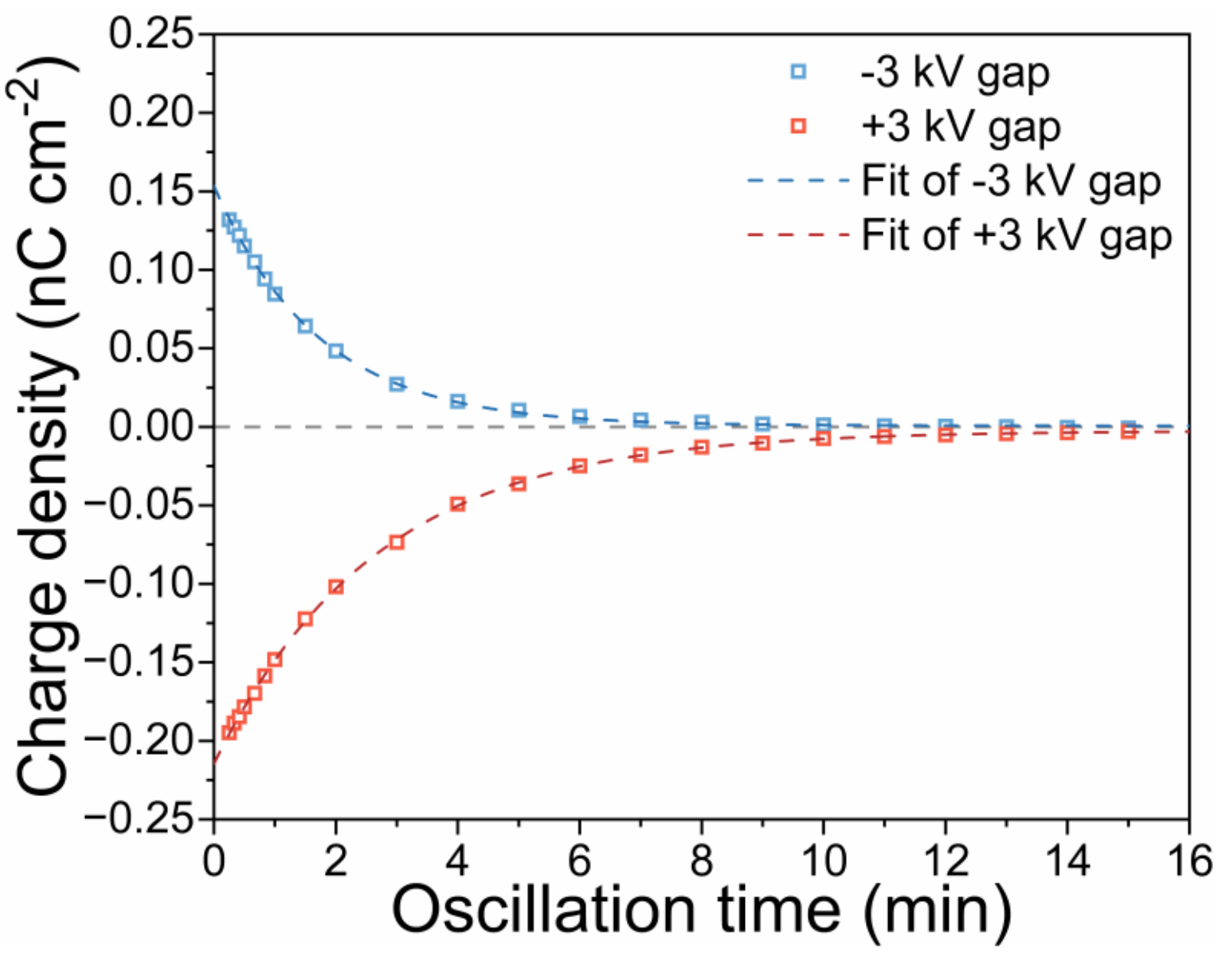

Figure S6. Charge density as a function of oscillation time after “gap” control test.

To isolate this contribution, we performed a gap control experiment, where electrical potential of ±3 kV was present. It was done by stopping program from performing contact step and sustaining an air gap instead, thereby preserving nearly the same electric field exposure but suppressing direct interfacial contact and material transfer. After the potential was switched off, the sample was subjected to the same oscillation protocol, and the induced charge density was recorded as a function of time (Fig. S6). In both polarity cases, the signal decayed exponentially toward zero without polarity change, confirming that this component originates from transient polarization rather than stable interfacial changes.

These results have now been included as part of control test paragraph in main text:

"In a gap-control experiment, the same ±3 kV potential was applied while direct contact was suppressed by maintaining an air gap (~100 μm) between the surfaces. Under these conditions, the induced charge decayed toward zero without polarity inversion, showing that electric-field exposure alone produces only a transient polarization contribution (Figure S6)."

4. In Figure S2, it is helpful to provide the corresponding SEM images to support the comparison of material surfaces after different high-voltage and contact treatments. In addition, the possibility that the applied high voltage induces surface roughening or damage due to electrical breakdown should be carefully considered.

Answer: Thank you for this valuable suggestion. In response, we prepared and characterized four PS samples: no contact, contact at 0 kV, contact at +3 kV, and contact at -3 kV. To provide a more comprehensive comparison of the surface state we added both SEM images and RMS roughness measurements to the revised Supporting Information.

The SEM images show that all samples retain largely similar morphology at the micrometer scale, with no evidence of large-scale surface damage, ablation, or crater-like features that would be expected from electrical breakdown (Fig. S3 C). At higher magnification, crack-like nanoscale contrast was observed on all Au-coated samples to some extent, indicating that this feature is influenced at least partly by the sputtered Au layer and imaging conditions. While the +3 kV sample exhibited somewhat more pronounced network-like contrast, this difference is subtle and does not indicate clear structural damage.

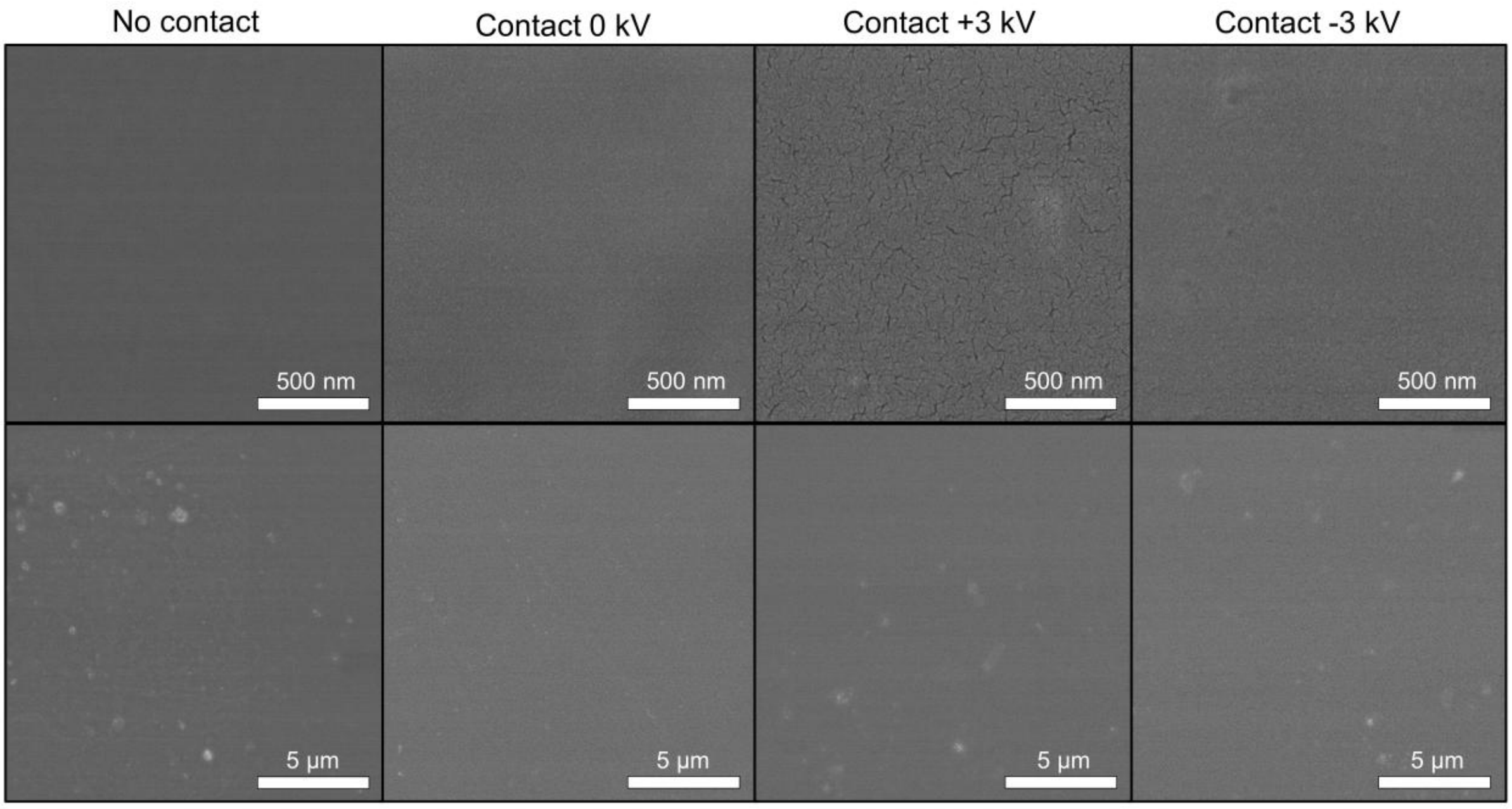

Figure S3. C) SEM images of sample that hasn't been contacted, sample that was contacted against another PS without electric potential, and samples that were contacted against another PS with electric potentials of ±3 kV at two magnifications.

To further assess whether the applied bias induced surface roughening, AFM measurements were performed on all four samples. The RMS roughness values remained comparable within experimental scatter (~7–10 nm), with overlapping deviations and no systematic trend as a function of contact or electric potential (Fig. S3 B). Since surface heterogeneity can influence same-material tribocharging, as recently shown by Sobarzo et al. for spontaneous ordering of identical materials into a triboelectric series, this control is important for excluding voltage-induced roughening as the origin of the measured charge response. Together with the SEM images, these results show no detectable surface damage or breakdown-induced roughening under the applied conditions.

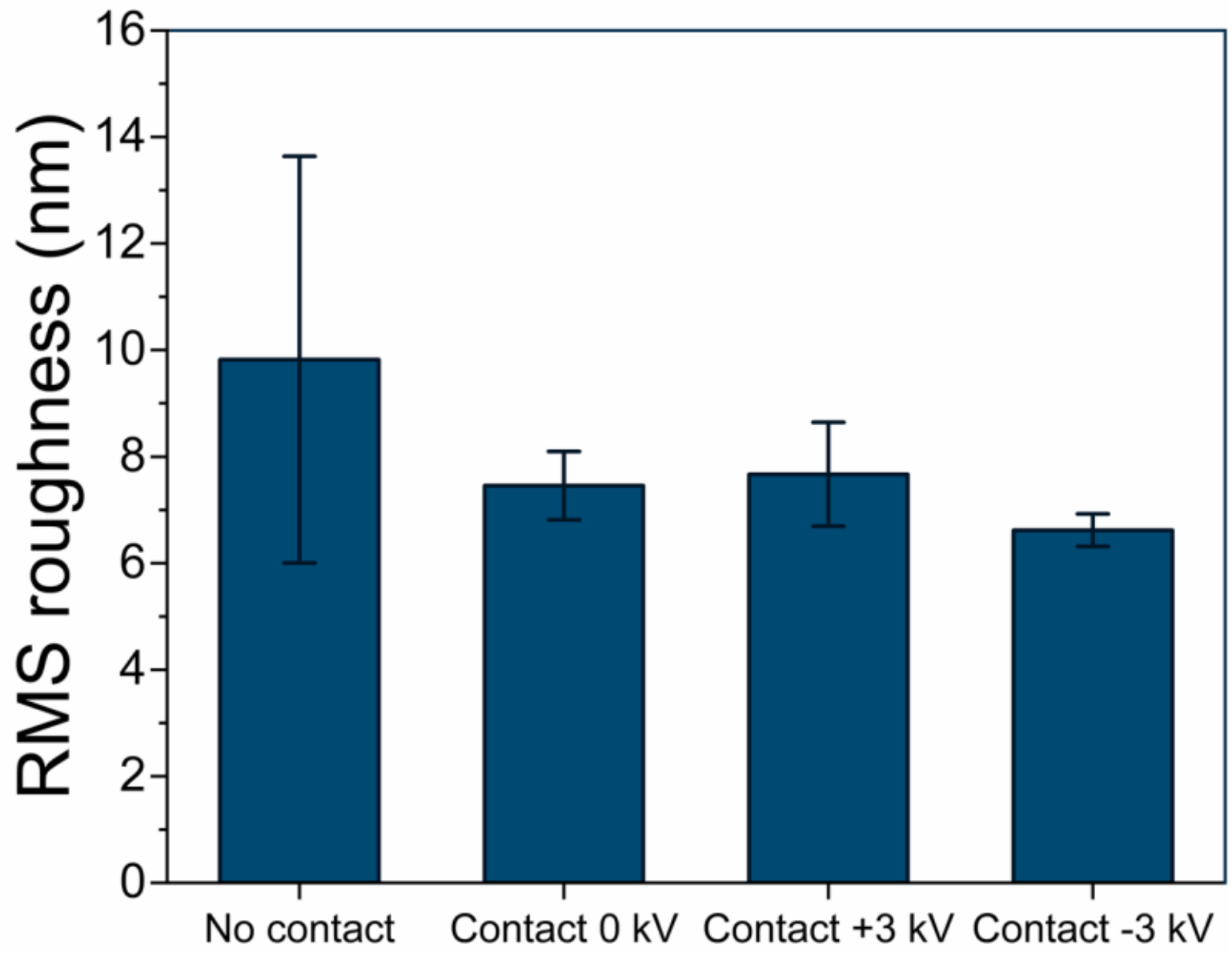

Figure S3. B) RMS roughness determined by AFM at three spots for each sample. Measurement area – 20×20 µm.

We have made changes to the text to reflect these results:

"Surface morphology was used to assess interfacial modification and possible voltage-induced damage. RMS roughness changes on initially smooth ITO after biased contact with PS are consistent with contact-induced surface modification or transferred material (Figure S3, A). SEM and RMS analysis of PS after no contact, 0 kV, +3 kV, and −3 kV contact showed no breakdown-related damage or systematic roughening (Figure S3, B, C). Since surface heterogeneity can influence same-material tribocharging, the unchanged RMS roughness after 0 kV and ±3 kV contact rules out measurable bias-induced roughening of PS under these conditions.[41]"

5. The manuscript states that the increase in RMS surface roughness after contact is consistent with material or adsorbate transfer associated with contact electrification (Figures S2 and S3), and this observation is extended across multiple dielectric polymers. However, it is not fully clear whether surface roughness alone can be taken as direct evidence of material transfer responsible for the observed changes in charge density. In particular, the experiments are conducted under the application of a relatively high external voltage (on the order of several kilovolts), whereas negligible charge density is reported in the absence of external bias. This raises an important question as to whether the observed surface roughening arises intrinsically from contact electrification–induced material transfer, or instead from voltage-induced effects such as dielectric breakdown, local discharge, or field-driven surface damage. The authors are encouraged to clarify how material transfer due specifically to contact electrification can be distinguished from surface modification induced by the applied

high electric field, and to discuss whether additional control experiments or morphological evidence (e.g., SEM) are available to rule out voltage-induced artifacts.

Answer: Regarding Figure S2 (Fig. S3 A in the revised manuscript), we acknowledge that the original submission contained an error in the sample description. The figure corresponds to ITO surfaces before and after biased contact against PS, included specifically to evaluate changes in the ITO surface, which is generally assumed to be smooth. This has been corrected in the revised version. In our new data from polymer-polymer contact electrification (discussed above) – a slight reduction in RMS is observed.
We have amended the text to read:
"Surface morphology was used to assess interfacial modification and possible voltage-induced damage. RMS roughness changes on initially smooth ITO after biased contact with PS are consistent with contact-induced surface modification or transferred material (Figure S3, A)."

6. Figure 1d presents a highly simplified schematic that does not sufficiently convey the underlying physical processes. In particular, it is difficult to understand how charge generation, distribution, or transfer occurs under positive and negative external DC bias, as well as in the absence of an applied voltage. A more detailed schematic explicitly illustrating the charge configurations and transfer pathways in each case would greatly improve clarity. In addition, the manuscript describes the process as "spontaneous," yet the experiments are performed under an applied DC electric field. The authors are encouraged to clarify the appropriateness of this terminology and to distinguish clearly between truly spontaneous charge transfer and field-assisted or field-driven processes.

Answer: We agree that the term spontaneous may introduce some uncertainty, as the electric field forms at an interface when the mechanical force is applied. As we contact-separate two identical PS films, the contact electrification is not expected when the external voltage is absent.

In the manuscript we have now changed "spontaneous" to "field-induced".

7. In the simulation section, PTFE is selected as the model polymer on the basis of the availability of its flexoelectric coefficient. However, the manuscript lacks a substantive discussion linking the theoretical simulations to the experimental observations. The authors are encouraged to more explicitly compare and contextualize the simulation results with the corresponding experimental findings, and to discuss the extent to which the PTFE-based model captures the behavior of the materials investigated experimentally. Strengthening the correlation between theory and experiment would significantly improve the rigor and interpretability of the work.

Answer: In our finite element simulations, PTFE was selected as the model polymer because its flexoelectric coefficient is well-characterized in the literature, allowing for exact quantitative calculations. Because the sign of the flexoelectric coefficient is largely universal across this class of dielectric polymers, the physical mechanism of field superposition demonstrated in our PTFE model applies universally to Polystyrene as well.

PTFE is nevertheless experimentally represented in this study: as shown in Figure S5, smooth PTFE follows the same field-dependent charge modulation observed for the other dielectric polymers. Thus, the PTFE simulations provide a quantitative model for the local field-generation mechanism, while the experimental PTFE data support the broader applicability of field-controlled charging across dielectric polymers.

Reviewer #2 (Remarks to the Author):

In this work, the authors investigate the driving forces behind contact electrification between dielectric polymers. The manuscript claims that electric fields generated by the flexoelectric effect drive charge separation. The authors demonstrate that applying an external direct current electric field during the contact and separation of identical polymers can dictate the magnitude and polarity of the resulting static charge. The authors couple these macroscopic external field experiments with computational simulations of flexoelectric fields induced by surface asperities. The manuscript reports a clever experimental setup using identical materials under external fields to isolate polarization effects. The main concern around the manuscript is the logical leap required to equate macroscopic external direct current fields with highly localized flexoelectric fields. The experimental data do not conclusively isolate flexoelectricity from other contact induced charging mechanisms. Therefore, I recommend this paper to be accepted after major revision.

Answer: We thank the reviewer for the comment and for acknowledging the experimental approach. In our experiments, chemically identical polymer pairs with the same surface roughness are used to minimize conventional contact electrification asymmetries and isolate the effect of the applied field. In this way, the external field is used as a controllable means to bias the interfacial charge separation, rather than to replicate the exact spatial distribution of flexoelectric fields.

We agree that flexoelectric fields are highly localized at nanoscale asperities, whereas our experimental setup applies a uniform macroscopic field. To bridge this gap and validate our approach, we have now performed a quantitative comparison using the experimental voltage and film thickness values together with the numerically derived surface-normal electric field from our FEM models.

For a representative 100 μm polymer film under a 3 kV bias, the external field is approximately 3×10^7 V/m. In the flexoelectric simulations at 15 nm displacement, the average surface-normal field is 4.3×10^7 V/m for the rougher contact geometry (r = 50 nm) and 1.9×10^7 V/m V/m for the smoother geometry (r = 250 nm), while the corresponding peak local values are 2.3×10^9 V/m and 3.7×10^8 V/m.

This shows that the externally applied field is comparable to the surface-averaged flexoelectric field and therefore can plausibly bias the interfacial charge-separation process, while the local flexoelectric field is strongly amplified at nanoscale asperities. We have added this quantitative comparison to the revised manuscript to eliminate any perceived logical leap.

In the revised manuscript we have included a new paragraph:

"To relate the macroscopic applied field to the simulated flexoelectric fields, their magnitudes were compared using the experimental voltage and film thickness. For a representative 100 μm polymer film under a 3 kV bias, the external field is approximately 3×10^7 V m^{-1}. In the flexoelectric simulations at 15 nm displacement, the average surface-normal electric field was 4.3×10^7 V m^{-1} for the rougher contact geometry (r = 50 nm) and 1.9×10^7 V m^{-1} for the smoother geometry (r = 250 nm), while the corresponding peak local fields reached 2.3×10^9 V m^{-1} and 3.7×10^8 V m^{-1}, respectively. Thus, the externally applied field is comparable to the surface-averaged simulated flexoelectric field, while local flexoelectric fields are strongly amplified at nanoscale asperities."

- The authors claim to provide conclusive evidence of flexoelectricity as the driving force for contact electrification. This builds heavily on recent proposals by Marks and Olson. Marks and Olson correlated flexoelectricity with triboelectricity using atomic force microscopy in Nano Letters in 2022. The authors must better distinguish their macroscopic external field approach from this prior research paper. The authors should discuss and add exactly what experimental novelty is introduced beyond the theoretical framework already established.

Answer: We thank the reviewer for this important point. The work by Marks and Olson indeed demonstrates a correlation between flexoelectricity and triboelectricity using AFM-based measurements on $SrTiO_3$, i.e., an inorganic ceramic system. In contrast, our study focuses on polymer materials, where such effects are significantly more challenging to probe directly.

The experimental novelty of our work lies in the use of a macroscopic external field during contact between identical polymers to systematically control both the magnitude and polarity of the generated charge. This approach allows us to probe the

role of flexoelectric fields in a way that is not accessible with AFM-based methods and extends the concept to soft, dielectric polymer systems.

We have updated the last paragraph in the introduction:

"Herein, we demonstrate experimentally that an external electric field alters the polarity and charge separation during polymer CE experiments. These simple experiments, performed using non-ferroelectric dielectric polymers, show that field-induced polarisation, which occurs during contact at high electric fields, relaxes to leave an opposing, more persistent surface charge, aligning with the observations of Marks and Olson and Mizzi et al. [5,24-27,47] Together with control measurements and flexoelectric simulations, these results support a mechanism in which interfacial electric fields act as transient driving forces that bias the separation of charged species during polymer CE. This provides an experimental route to test whether native strain-gradient-induced fields, such as those generated at rough or compliant polymer contacts, can drive polymer contact electrification."

- In the manuscript, an external field applies a uniform macroscopic polarization across the entire bulk polymer. Flexoelectric fields are generated by highly localized strain gradients at nanoscale asperities. Showing that a massive external field directs charge transfer does not explicitly prove that native flexoelectric fields do the same under normal conditions.

Answer: To explicitly prove that the macroscopic field selectively targets and superimposes with the native flexoelectric mechanism at the nanoscale, we developed FEM simulations of coupled solid mechanics and electrostatics contact model.

We simulated the polymer asperity undergoing a 15 nm mechanical compression. To replicate our macroscopic experimental conditions (30 MV/m), we applied an external bias of ±90 V across the vertical domain. We have included two new figures in this rebuttal to demonstrate the superposition:

1. Figure 1 (Normal Electric Field, En, vs. Displacement): This plot tracks the total normal electric field precisely at the contact asperity. As the tip compresses, the native flexoelectric field (0 V baseline, black curve) naturally scales with the mechanical deformation. When the macroscopic fields are applied (+90 V and -90 V), they do not overwrite the flexoelectric behavior; rather, they perfectly and dynamically superimpose on top of it as the asperity deforms.
2. Figure 2 (Normal Polarization, Pn, vs. Displacement): This plot reveals the fundamental mechanism at play. Total polarization consists of the mechanically-induced flexoelectric polarization plus the field-induced dielectric polarization. As seen in the figure, the external bias creates a constant, immediate dielectric shift (the vertical spacing between the curves at 0 nm displacement). However,

the curved evolution of the polarization, which represents the physical driving force for charge transfer, is mathematically identical across all three states.

These quantitative models prove that the fundamental mechanical driving force (the flexoelectric strain gradient) remains untouched, while the macroscopic external field successfully funnels into the nanoscale contact point to selectively cancel or reinforce the native field. Because they physically superimpose at the exact interface, they act on the exact same physical mechanism.

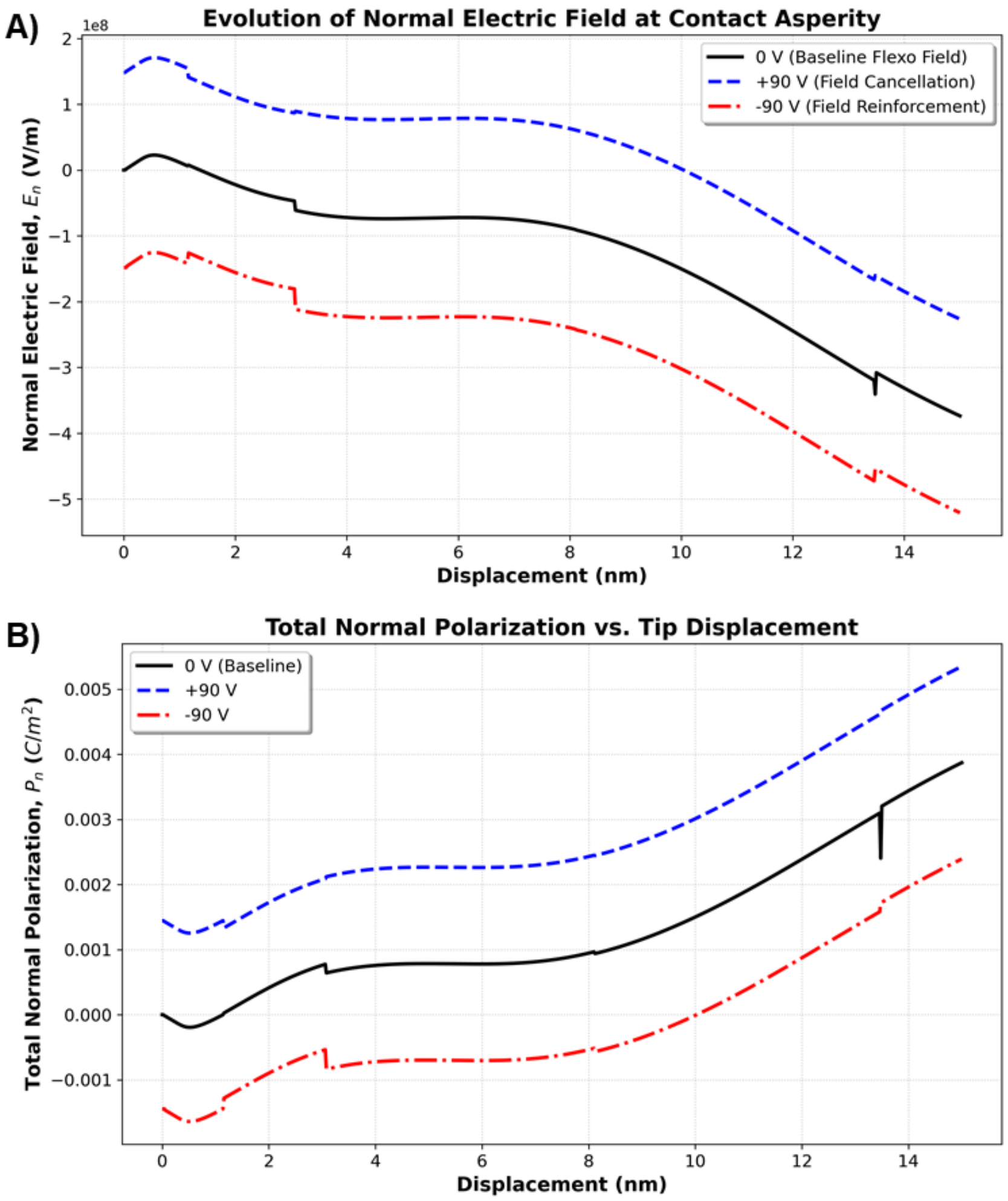

Figure S10. Biased flexoelectric-field simulations at the contact asperity. A) Averaged normal electric field, E_n, as a function of compressive displacement under 0 V, +90 V, and −90 V bias. B) Corresponding averaged normal polarisation, P_n, showing a bias-induced offset superimposed on the displacement-dependent flexoelectric response.

This figure together with a separate discussion have now been included in the Supplementary information of revised manuscript:

*"**Biased flexoelectric-field simulations***

Additional biased simulations were performed to examine whether an externally applied macroscopic field can reinforce or oppose the local flexoelectric field generated at a compressed asperity. The same coupled solid mechanics/electrostatics model was used as in the main flexoelectric simulations, with a 15 nm maximum compressive displacement. To approximate the experimental macroscopic field strength of approximately 30 MV m^{-1}, external biases of $\pm$90 V were applied across the computational domain. The normal electric field and normal polarisation were then extracted at the contact interface as a function of displacement.

In the unbiased case (0 V), mechanical compression alone generated a localized flexoelectric field at the asperity. The averaged normal electric field, E_n, increased with displacement and reached −3.73 × 10^8 V m−1 at 15 nm compression (Figure S10 A). When the applied field was aligned with the flexoelectric field (−90 V), the local interfacial field was reinforced, reaching −5.21 × 10^8 V m^{-1} at the same displacement. Conversely, when the applied field opposed the flexoelectric field (+90 V), the local field was partially suppressed, reaching −2.26 × 10^8 V m^{-1}. These results show that the externally applied field does not replace the mechanically generated flexoelectric field, but shifts its magnitude depending on field polarity.

The corresponding normal polarisation, P_n, provides the mechanistic explanation for this behaviour (Figure S10 B). The applied bias produces an immediate dielectric-polarisation offset even at zero displacement, visible as a vertical shift between the curves. Upon compression, however, all three cases show the same displacement-dependent increase associated with the strain-gradient-induced flexoelectric contribution. Thus, the total interfacial polarisation consists of two superimposed terms: a field-induced dielectric component and a mechanically induced flexoelectric component. This supports the interpretation that an external macroscopic field can penetrate the dielectric contact geometry and either reinforce or oppose the localized flexoelectric field at the asperity, depending on polarity."

- The manuscript dismisses electron transfer by stating band structure alignment is only relevant for electron transfer. The authors assume organo ions or adsorbates are the primary charge carriers. They do not explain how a macroscopic electric field selectively templates the transfer of massive organo ions rather than electrons.The authors should explain mechanistically to support this claim.

Answer: Electron transfer is not the governor mechanism in polymers. This has been discussed in many works [Lacks, D. J. & Shinbrot, T. Long-standing and unresolved issues in triboelectric charging. Nature Reviews Chemistry 3, 465-476 (2019).; Fang, Y. et al. Static charge is an ionic molecular fragment. Nature Communications 15, 1986 (2024)]. While widely accepted in TENG community which is mostly focusing on developing generator devices, the community working on investigation of contact

electrification mechanisms of polymer insulators well before 2012 (more than 100 years), is strongly against the electron transfer in between polymers with no free electrons, acceptor or donor states. Even defects - considered as sites for electron transfer, are insufficient - 5 orders of magnitude difference in density of defects vs charge density [Waitukaitis, S. R., Lee, V., Pierson, J. M., Forman, S. L. & Jaeger, H. M. Size-Dependent Same-Material Tribocharging in Insulating Grains. Physical Review Letters 112, 218001 (2014)]. Organoions formed during polymer contact separation will be attracted by opposite electric field on the opposite electrode.

We have now updated the schematic in Figure 1 D to more clearly reflect the proposed mechanism.

- The main contact electrification experiments use polystyrene. The simulations in Figure 2B use polytetrafluoroethylene. The authors justify this by stating nearly all polymers show a flexoelectric coefficient of the same sign. Comparing experimental data of one material with simulations of another introduces unnecessary quantitative inconsistencies. The authors should simulate polystyrene or provide corresponding rough and smooth experimental data for polytetrafluoroethylene.

Answer: In our finite element simulations, PTFE was selected as the model polymer because its flexoelectric coefficient is well-characterized in the literature, allowing for exact quantitative calculations. Because the sign of the flexoelectric coefficient is largely universal across this class of dielectric polymers, the physical mechanism of field superposition demonstrated in our PTFE model applies universally to Polystyrene as well.

We also considered whether an equivalent rough/smooth PTFE experimental comparison could be performed. However, preparing PTFE surfaces with controlled, reproducible roughness comparable to the PS rough/smooth series is experimentally challenging because PTFE is chemically inert, has very low surface energy, and cannot be readily solution-cast or templated in the same way as PS. Mechanical abrasion, plasma treatment, or etching could introduce uncontrolled changes in surface chemistry, crystallinity, damage, or trapped charge, which would add confounding factors rather than provide a clean roughness-only comparison. For this reason, the direct rough/smooth experimental comparison is performed using PS, where the surface structure can be controlled reproducibly while maintaining the same polymer chemistry.

Importantly, PTFE is still experimentally represented in the broader polymer series shown in Figure S5. Smooth PTFE follows the same field-dependent charge modulation observed for the other dielectric polymers, supporting the generality of electric-field-controlled charging across insulating polymers. Thus, the PTFE simulations provide a quantitative model for the local flexoelectric-field generation mechanism, the smooth

PTFE data support the broader applicability of the field-control effect, and the rough/smooth PS experiments provide the direct experimental validation for the role of surface geometry.

- The procedure to extract the stable surface charge is not entirely convincing. The authors fit an exponential decay to separate the spontaneous polarization from the stable static charge in Figure S1. It is difficult to rule out the formation of long lived electrets or deep trapped charges caused by the high external voltage. It would clarify this point by showing control experiment to prove that the residual charge is genuinely identical in nature to standard contact electrification.

Answer: We agree that distinguishing between polarization, electret effects, and contact electrification is important. In our case, polarization-related contributions exhibit opposite polarity to that expected from material transfer and decay rapidly. This is also consistent with the observation that a simple ethanol rinse reduces the measured charge close to zero, which would not be expected for deeply trapped or long-lived electret charges (Fig. S8). These results have been included in the revised manuscript as part of paragraph about control tests:

"Finally, ethanol rinsing reduced the measured charge close to zero, supporting the surface-localized nature of the persistent charge and making a purely electret explanation less likely (Figure S8)."

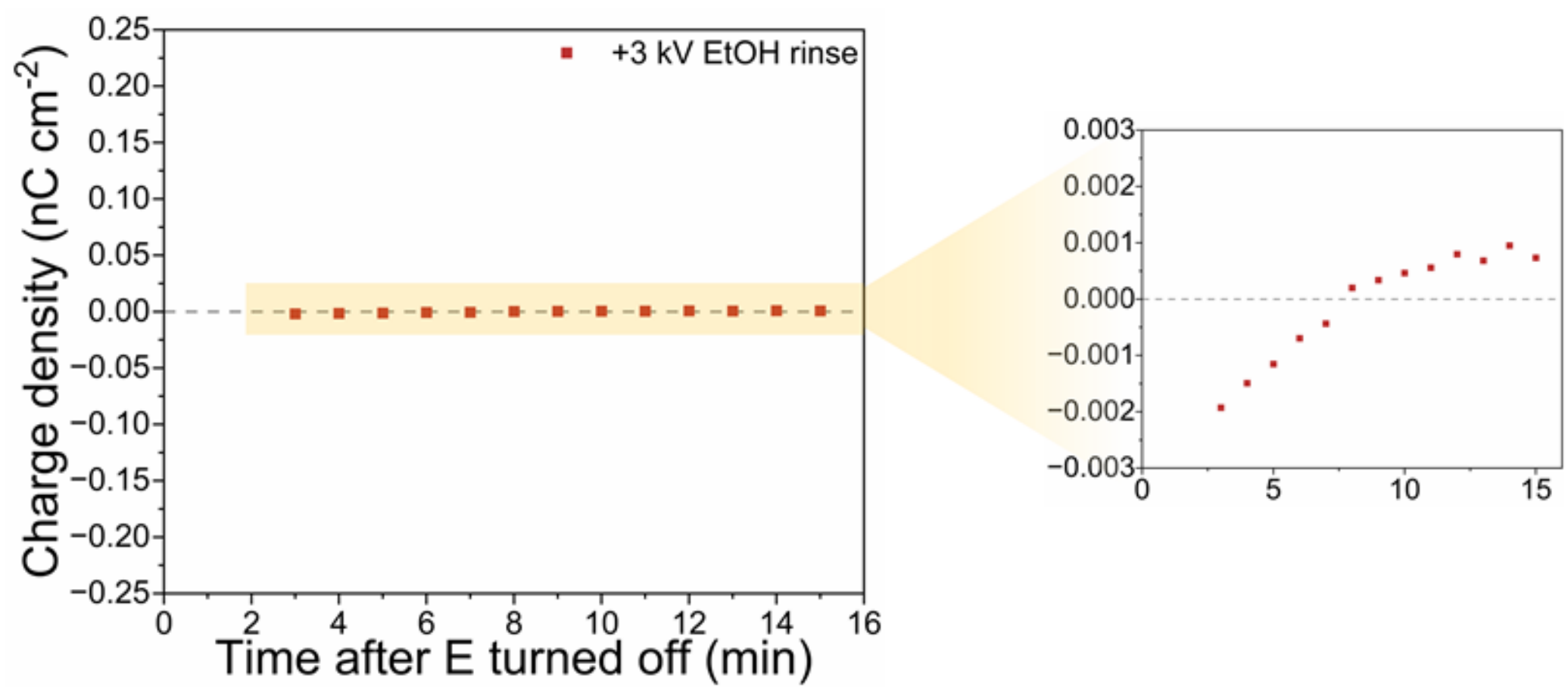

Figure S8. Charge density as a function of time after PS was rinsed with EtOH before measurement.

The exponential decay fitting was applied only to describe the transient signal immediately after the external field is switched off, as the first measurement point is obtained ~15 s later. Therefore, this analysis does not represent a fully stabilized charge state, but rather separates the short-lived polarization contribution from the charge generated during field-assisted contact.

Clarification has now been made in the revised manuscript:

"The measured surface charge decay was fitted using an exponential function and extrapolated to estimate the initial charge response at $t \approx 0$"

Regarding the comparison to standard contact electrification, we propose that the driving force for charge transfer, including its polarity, is governed by the flexoelectric field at the interface. In this work, the externally applied field is used to simulate and amplify this effect, enabling controlled variation of both charge magnitude and polarity. This clarification has been added to the revised manuscript:

"The externally applied field should not be interpreted as an exact spatial replica of the native flexoelectric field generated during contact. Flexoelectric fields are highly localized at nanoscale asperities, whereas the applied DC field is macroscopic. Here, the external field is used as a controllable way to showcase that the direction of an interfacial electric field can bias the polarity of persistent charge generated during polymer contact."

- The discussion of compressive modulus in Figure S4 needs stronger controls. Comparing polymers with widely different Youngs moduli inherently adds multiple confounding factors. These polymers also differ in surface energy and cohesive energy. Yet the authors attribute the change in charge exclusively to increased flexoelectric polarization from larger deformation. To support this conclusion, they should experimentally vary modulus while keeping the other chemical properties effectively constant.

Answer: We agree that comparing chemically different polymers introduces possible confounding factors, including surface energy, cohesive energy, crystallinity, and chemical composition. Therefore, we have revised the discussion to avoid implying that the observed charge differences arise exclusively from compressive modulus. As an additional control, we compared two chemically similar silicone elastomers, Ecoflex 00-30 (PDMS 30) and Ecoflex 00-50 (PDMS 50), with reported Young's moduli of approximately 70 and 83 kPa, respectively. This comparison keeps the polymer chemistry broadly similar while introducing a modest difference in mechanical stiffness. As shown in the added data, PDMS 50 produces slightly lower charge densities than PDMS 30 under both +3 kV and −3 kV bias conditions (Figure S12). This is consistent with the proposed role of deformation-related polarization, since the slightly stiffer elastomer should undergo somewhat smaller deformation under the same contact force.

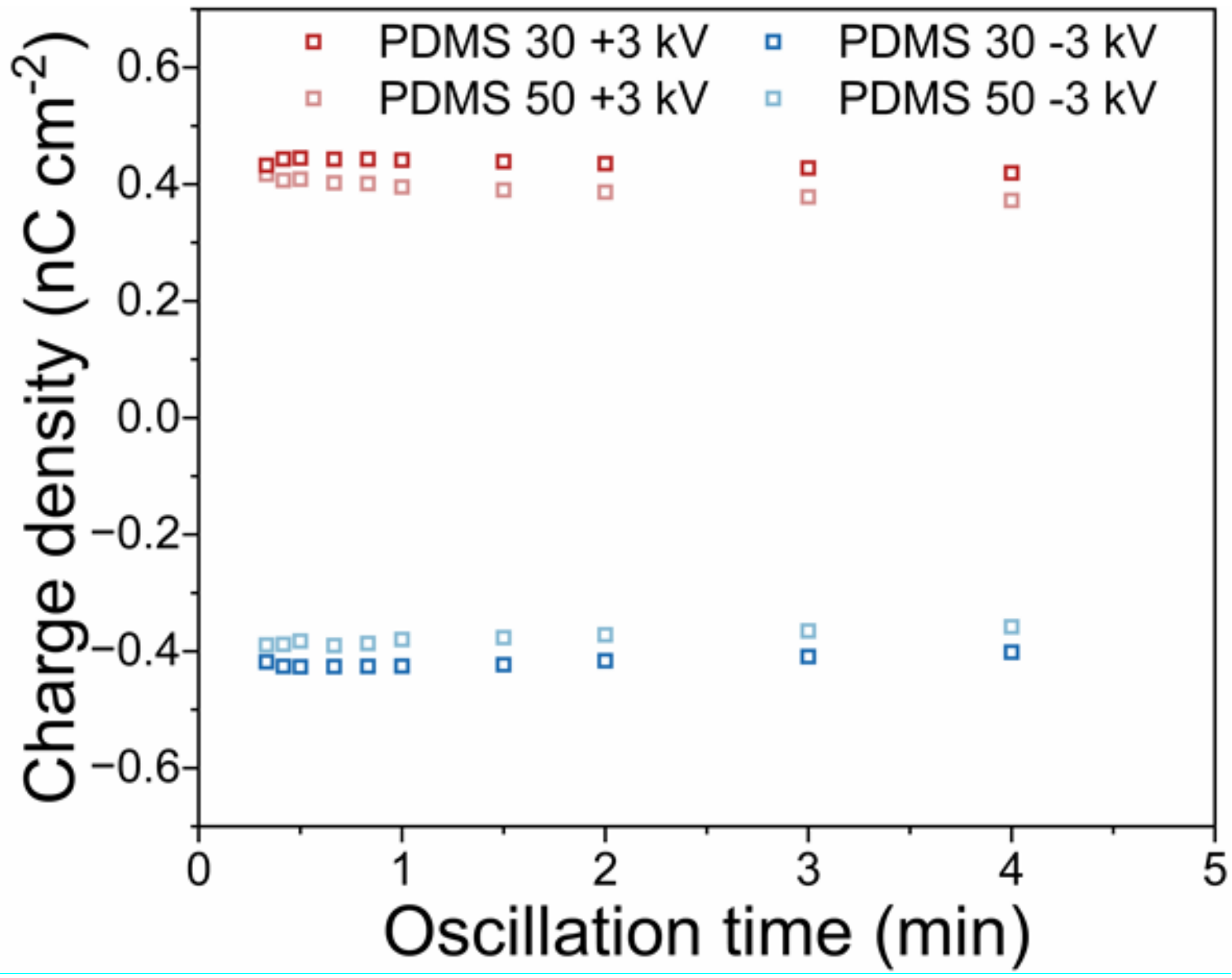

Figure S12. Charge density comparison as a function of oscillation time for PDMS 30 and PDMS 50 elastomers after biased contact separation using ±3 kV.

However, we agree that this experiment cannot fully isolate modulus as the only variable, because even within a single polymer family, changes in modulus can be accompanied by differences in crosslink density, viscoelasticity, adhesion, and surface energy.

- The manuscript contains several grammatical errors and typos that hinder clarity. The title uses incorrect subject verb agreement with the phrase Electric Fields Controls. The word performed is misspelled in the caption of Figure 1.

Answer: We thank the reviewer for noting these issues. The grammatical errors and typos have been corrected in the revised manuscript.

Reviewer #3 (Remarks to the Author):

The paper by Sutka et al about the influence of applied electric fields on the charge separation process is interesting but not very surprising. The topic is of general interest and the study add a useful contribution to this topic but I am not convinced if it should be published in Nature. I note that there is an earlier theory study [see 2020 EPL (Europhysics Letters) 129(1):10006] about the influence of surface roughness on the flexoelectric effect so that part of the paper is not very novel. But I liked the video presentations of the flexoelectric effect.

Answer: We thank the reviewer for the constructive comments and for highlighting the prior work. We agree that flexoelectric effects, including those related to surface roughness, have been discussed previously. However, in our work flexoelectricity is not

considered as the direct source of the measured surface charge, since the strain gradient relaxes upon separation. Instead, we propose that the flexoelectric field acts as a driving force for organoion transfer during contact, which leads to the persistent charge observed. This clarification has been made more explicit in the revised manuscript.

Reviewer #4 (Remarks to the Author):

The manuscript reports that externally applied electric fields can control the polarity and magnitude of surface charge generated during contact electrification between polymers, and proposes that flexoelectric fields generated during mechanical contact are the underlying driving force for this phenomenon. While the topic is interesting and potentially relevant to the fundamental understanding of triboelectrification, the current manuscript does not provide sufficiently rigorous experimental evidence to support the proposed mechanism. In particular, the relationship between externally applied electric fields and flexoelectric polarization is not convincingly demonstrated, and several alternative explanations remain plausible. Therefore, this reviewer does not recommend publication of the manuscript in its current form. The following points should be carefully addressed.

Answer: Thank you for the careful evaluation. We acknowledge the concerns regarding the experimental support and the interpretation of the mechanism, and we have revised the manuscript to clarify our claims, strengthen the discussion, and address alternative explanations.

Comment 1

The authors claim that an externally applied electric field can control the polarity of surface charge generated during contact electrification. However, the physical mechanism underlying this phenomenon is not clearly established. Although the authors introduce a protective polymer layer to prevent direct charge injection from the metal electrode, it is well known that strong electric fields can induce dipole alignment or partial poling in dielectric polymers. Such electric-field-induced polarization can produce transient surface charge distributions even without triboelectric processes. Therefore, it remains unclear whether the observed charge inversion truly originates from contact electrification or simply from electric-field-induced polarization effects.

Answer: We agree that strong electric fields can induce polarization or electret-like effects in polymers. However, in our measurements this contribution gives a surface charge polarity opposite to that expected from the externally biased contact electrification process. The initial polarization-related signal is transient and decays relatively quickly, whereas the longer-lasting charge follows the polarity expected from contact-induced material transfer. This distinction is already shown in the manuscript

and has now been clarified further in the revised version in Fig. 1 D. Furthermore, we have included a series of control tests and the revised manuscript now has a paragraph: "Several control experiments were performed to distinguish persistent contact-generated charge from purely field-induced polarization, electret-like charging, or other transient electrostatic effects. In a gap-control experiment, the same ±3 kV potential was applied while direct contact was suppressed by maintaining an air gap (~100 µm) between the surfaces. Under these conditions, the induced charge decayed toward zero without polarity inversion, showing that electric-field exposure alone produces only a transient polarization contribution (Figure S6). Extended non-contact oscillation measurements further showed that, after the initial decay and polarity inversion, the remaining charge persisted for at least 60 min, although it gradually decreased under ambient conditions (Figure S7). Finally, ethanol rinsing reduced the measured charge close to zero, supporting the surface-localized nature of the persistent charge and making a purely electret explanation less likely (Figure S8)."

In particular, several key questions remain unresolved:
- Electric field penetration through the protective polymer layer
The manuscript assumes that the protective polymer layer eliminates direct electrical influence from the metal electrode. However, depending on the dielectric constant and thickness of the protective layer, a substantial electric field may still penetrate into the upper polymer layer. Under sufficiently high voltages (±3 kV in this work), this field could induce polarization or charge trapping in the polymer. Quantitative analysis of the electric field distribution across the multilayer structure is therefore necessary.

Answer: We performed a quantitative electrostatic finite element analysis of the exact experimental multilayer stack. We modelled a 0.05 mm Kapton protective layer (eps_r=3.4) on top of a 0.1 mm Polystyrene film (eps_r=2.6), subjected to the 3 kV external bias used in our experiments.

As shown in the figure below, the protective layer does not eliminate the external field. Instead, the electric potential distributes strictly according to the dielectric constants of the layers. The 1D cut-line plot of the electric field norm demonstrates that a uniform macroscopic field of approximately 21 MV/m penetrates and saturates the bulk of the Polystyrene film. This confirms that an electrostatic driving force reaches the polymer surface during contact, validating our experimental premise. This is a high field, but still well below the typical DC breakdown strength of polystyrene films, which is usually > 100–200 kV/mm (and often ~300–500 kV/mm for thin films in good condition).

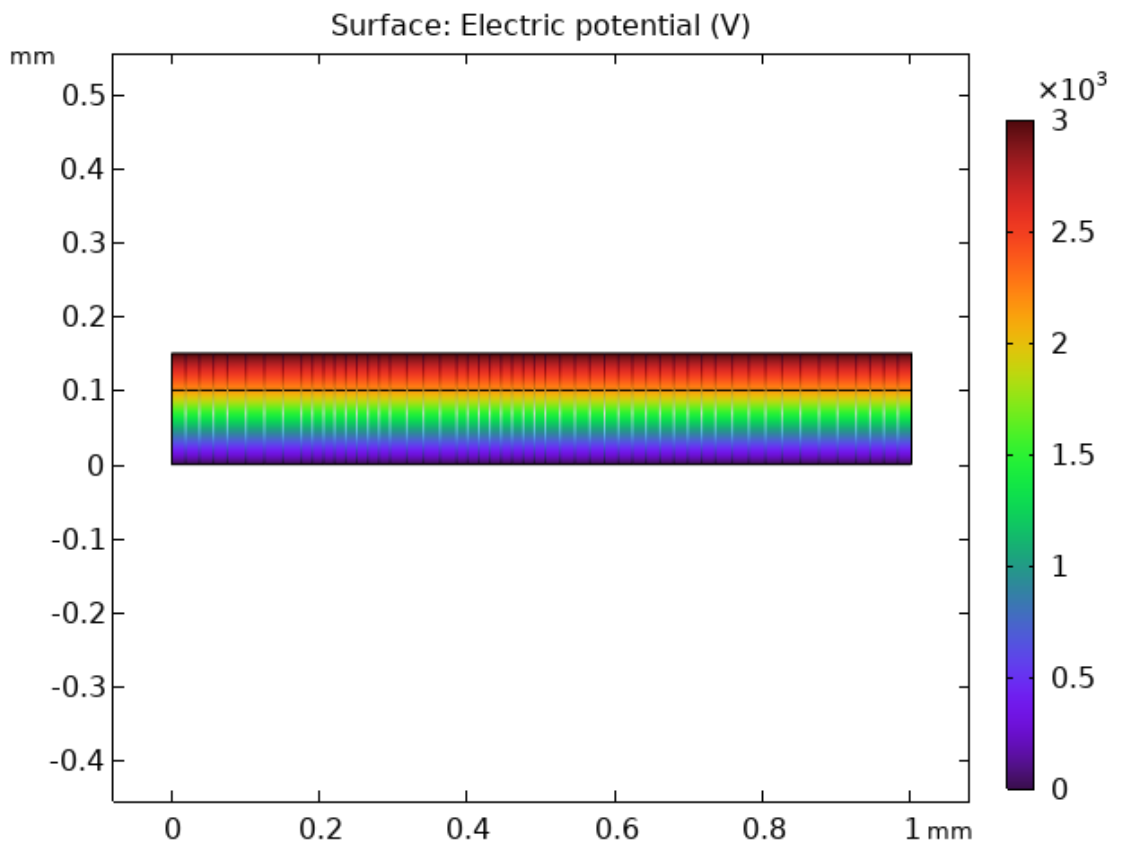

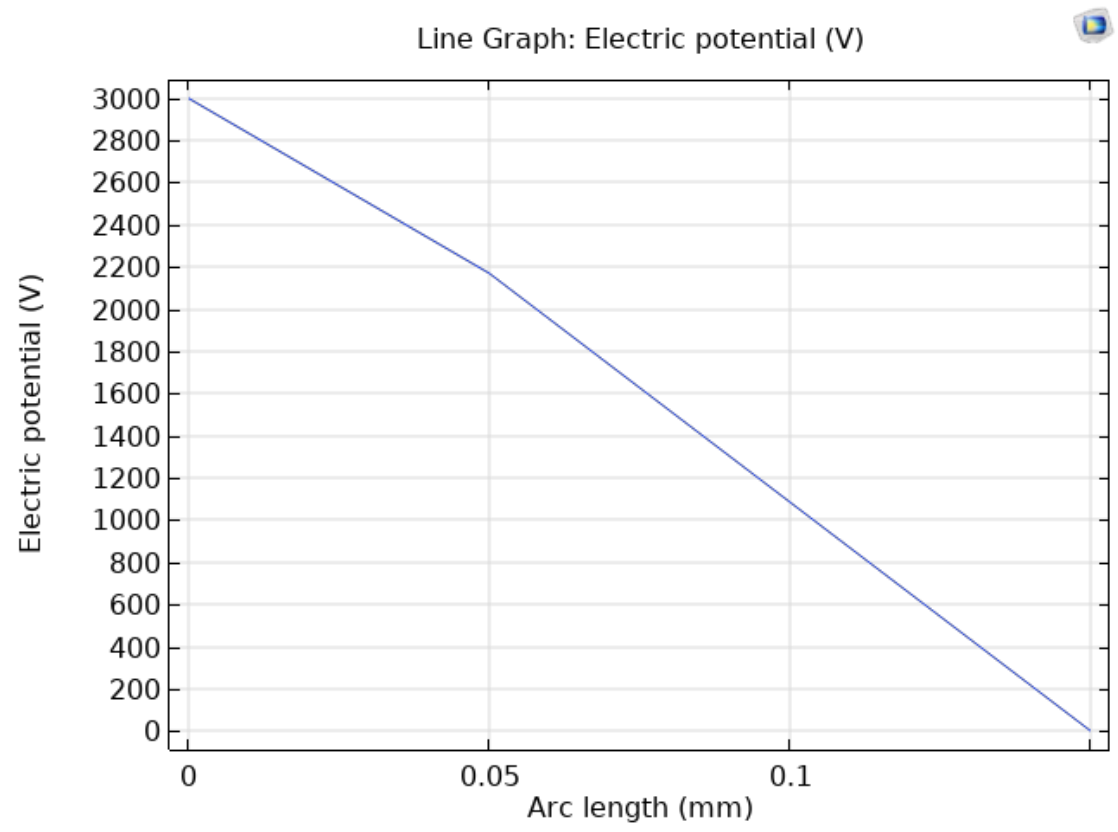

These results have been addressed in the revised manuscript:

"Importantly, this protective layer prevents direct metal-polymer contact and charge injection, but does not screen the applied electric field. Because the protective and measured layers are dielectric polymers, the applied potential is distributed across the polymer stack, allowing an electric field to reach the polymer-polymer interface during biased contact-separation. A representative Kapton/PS dielectric-barrier simulation confirmed this general behaviour, showing field penetration through the protective layer rather than field screening (Figure S1)."

- Stability of the measured surface charge

If the observed surface charge originates from electric-field-induced polarization or electret-like charge trapping, the temporal stability of the charge becomes an important indicator. The manuscript reports charge decay over several minutes, but it is not clear whether the remaining charge persists over longer timescales (e.g. hours or days), which is commonly reported for triboelectric charges.

Answer: Thank you for this valuable comment. To clarify the temporal stability of the measured surface charge, we performed oscillation measurements for up to 60 min after treatment with both +3 kV and -3 kV potential. As shown in the newly added data (Supporting information Figure S7), the charge density exhibits a more pronounced decrease/polarity switch during the initial minutes, followed by a significantly slower decay over the remaining measurement period. Importantly, in both polarity cases, a clearly measurable non-zero charge remains after 1 h of continuous oscillation, indicating that the induced surface state is not limited to only a short-lived minute-scale effect.

For the +3 kV treatment, the charge stabilizes at approximately +0.025 nC cm^{-2}, while for the −3 kV treatment around −0.036 nC cm^{-2}. After an hour it has dropped to +0.013 nC cm^{-2} and -0.019 nC cm^{-2}, respectively. Although some gradual decay is still observed, such behavior is expected under ambient conditions and repeated oscillatory measurement, where charge dissipation through air, adsorbed moisture, and surface

conduction can occur. Therefore, these results demonstrate that the remaining charge persists over at least hour-long timescales, which supports the presence of a relatively stable surface charge.

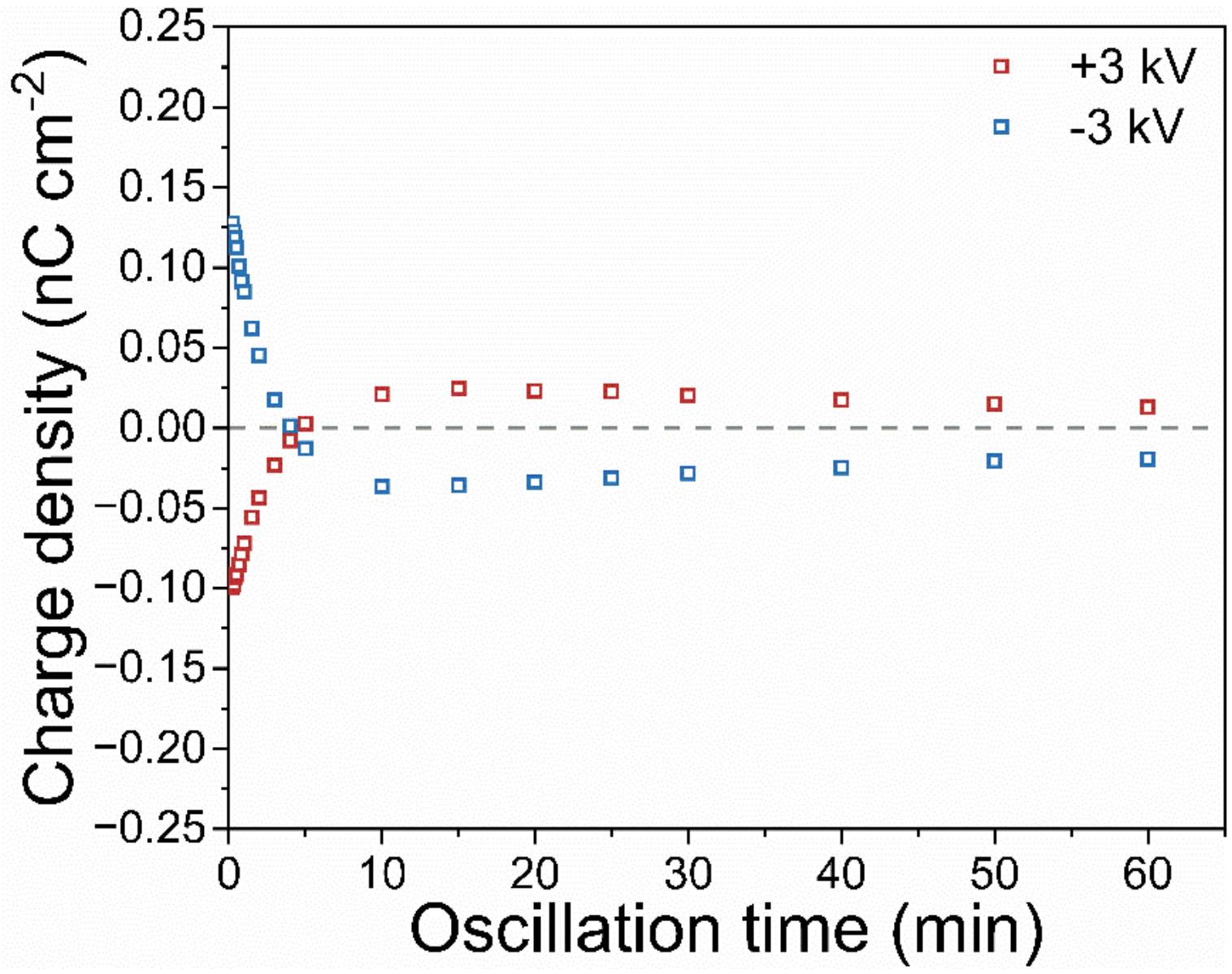

Figure S7. Charge density as a function of non-contact oscillation time in the long-term stability test.

Results have been included as part of broader control test paragraph:

"Extended non-contact oscillation measurements further showed that, after the initial decay and polarity inversion, the remaining charge persisted for at least 60 min, although it gradually decreased under ambient conditions (Figure S7)."

- Role of repeated friction cycles

The authors perform 500 contact–separation cycles prior to measuring the charge using non-contact oscillation. The rationale for this procedure is not sufficiently justified. If the effect is primarily driven by the applied electric field, it would be important to show control experiments in which the polymer is simply exposed to the electric field without repeated friction. Such experiments would help distinguish between triboelectric charging and electric-field-induced charge accumulation.

Answer: Thank you for this important comment. We agree that the role of the 500 contact–separation cycles should be better justified. The selected number of cycles was chosen for two reasons. First, in conventional contact separation triboelectric measurements, the initial cycles often show gradual signal growth before reaching a stable or saturated charge density. Therefore, 500 cycles were used to ensure that the system reached a reproducible steady-state interfacial condition rather than reflecting transient behavior from the first few contacts.

Second, in the present system, these repeated cycles allow the interface to evolve under simultaneous mechanical contact and electric field exposure, where two competing contributions develop with different characteristic timescales. The faster contribution is the field-induced polarization state, which initially gives a charge polarity opposite to the applied bias, i.e., a negative charge density after +3 kV treatment and a positive charge density after -3 kV treatment. To isolate this purely electric field driven effect, we performed a control experiment in which the same ±3 kV potential was applied while the programmed contact step was disabled, maintaining an air gap instead. This preserved nearly the same electric field exposure but eliminated repeated friction, direct interfacial contact, and material transfer. After the potential was switched off, the sample was subjected to the same non-contact oscillation measurement (Fig. S6). In both polarity cases, the induced charge density decayed exponentially toward zero, confirming that electric-field exposure alone produces only a transient polarization contribution.

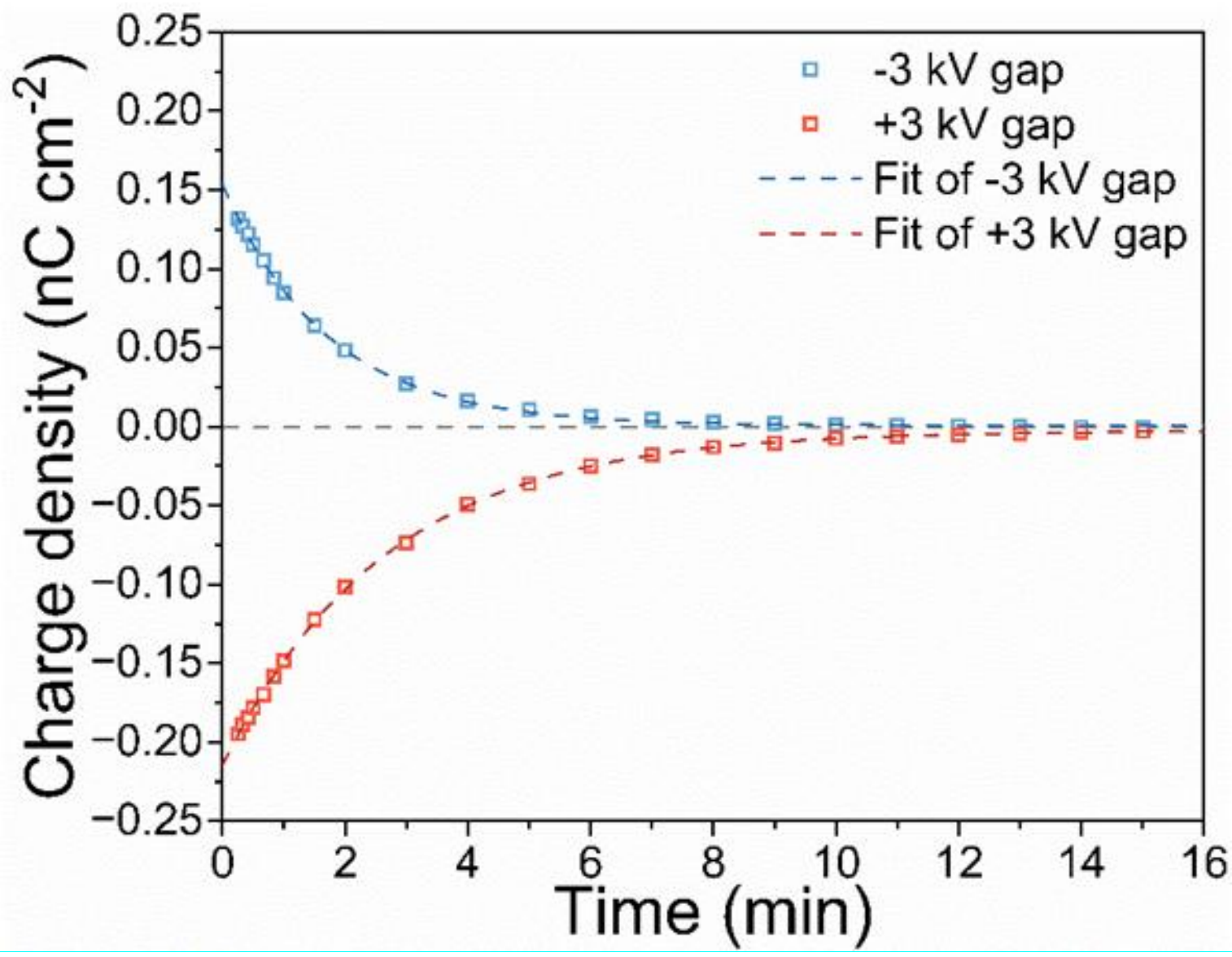

Figure S6. Charge density as a function of oscillation time after "gap" control test.

In contrast, under repeated contact separation cycles, a slower and more persistent contribution emerges from electric field assisted chain scission and subsequent material transfer at the polymer-polymer interface. This transferred material carries a stable polarity corresponding to the electric potential used in our experiments, i.e., positive after +3 kV treatment and negative after −3 kV treatment. As the transient polarization component relaxes, the transferred material contribution progressively dominates the signal. Therefore, the 500 cycles are important not only to reach saturation, as commonly required in triboelectric measurements, but also to allow sufficient interfacial mechanochemical evolution for the long-lived charge state to emerge.

Gap test results have been included in the main text as part of broader control test paragraph:

"In a gap-control experiment, the same ±3 kV potential was applied while direct contact was suppressed by maintaining an air gap (~100 μm) between the surfaces. Under these conditions, the induced charge decayed toward zero without polarity inversion, showing that electric-field exposure alone produces only a transient polarization contribution (Figure S6)."

Comment 2
The manuscript attempts to correlate the externally applied electric field effect with flexoelectricity induced by surface roughness. While this idea is intriguing, the experimental evidence presented here is insufficient to establish such a relationship. Flexoelectric polarization arises from strain gradients at the nanoscale, and the direction and magnitude of the polarization strongly depend on the local mechanical deformation field. In contrast, the externally applied electric field in this work is a macroscopic uniform field. Directly equating these two effects appears to be an oversimplification. The authors suggest that flexoelectric fields generated at rough surfaces may either enhance or cancel the polarization induced by the external DC electric field. However, this hypothesis is not supported by rigorous experimental verification. To substantiate this claim, more direct measurements of flexoelectric responses are required. For example:
- Atomic force microscopy-based measurements (e.g., piezoresponse or Kelvin probe force microscopy) could be used to detect local polarization or surface potential changes associated with flexoelectric deformation.

Answer: While AFM-based techniques such as PFM or KPFM are powerful tools for probing local polarization and surface potential, their direct application to flexoelectric effects in polymer systems remains highly challenging and, to date, not well established. Prior studies, including those by Marks and co-workers, have primarily demonstrated AFM-based flexoelectric measurements in ceramic materials, while noting that extension to polymers is possible however non-trivial.

This limitation arises from the inherently low flexoelectric coefficients of polymers, their viscoelastic nature, and the difficulty in generating and resolving sufficiently large and well-defined strain gradients at the nanoscale without introducing artefacts or signal ambiguity. As a result, such approaches have not been widely adopted or validated for polymer-based systems.

Due to these constraints, we have instead employed an alternative experimental strategy, where flexoelectric-like polarization is indirectly probed through controlled external electric field application during contact of identical polymer films. This

approach allows us to simulate the expected response under conditions more representative of the macroscopic contact electrification process studied here, while avoiding the methodological limitations associated with AFM-based techniques in polymers.

- Direct measurements of surface potential distributions could help verify whether strain-induced polarization fields are indeed present during contact.

Answer: To address the suggestion, we performed KPFM measurements of surface potential distributions on smooth and rough PS films right after 500 contact-separation cycles. In this test no electric potential was applied during contact-separation. The smooth PS surface showed an average potential of −2.11 V, whereas the rough PS surface showed an average potential of +0.491 V, indicating a clear polarity difference (Figure S9). This trend is consistent with our previous observations, where rougher surfaces charge positively and smoother surfaces negatively when identical polymers are contacted (10.1016/j.nanoen.2022.107914). This behavior is attributed to differences in the local mechanical state during contact, where surface roughness modifies the strain distribution and consequently the charge transfer process. While KPFM does not directly probe the polarization field during contact, the measured post-contact surface potentials support the relevance of strain-induced polarization/strain-controlled charging effects in this system.

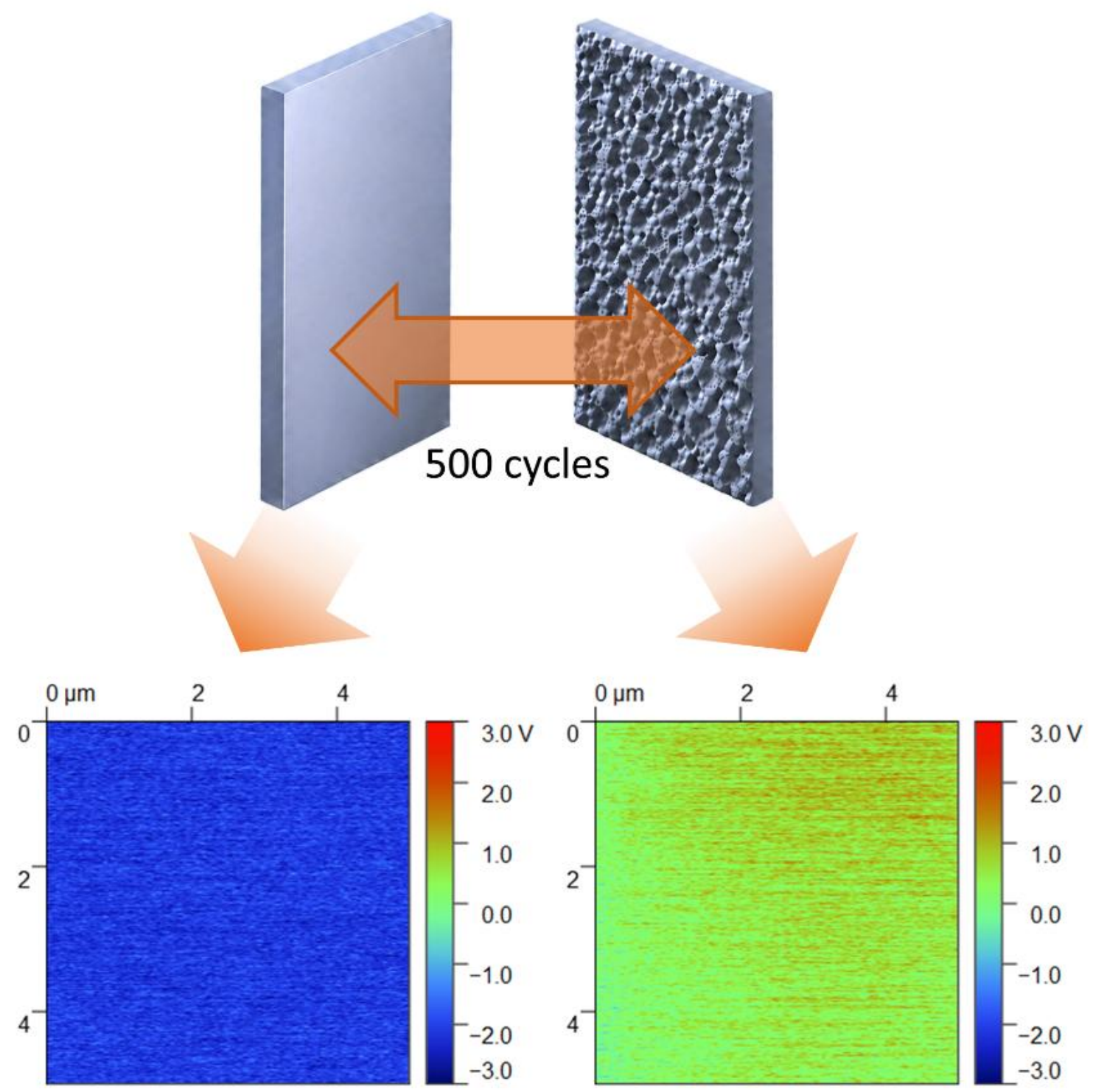

Figure S9. KPFM measurements of smooth (left) and rough (right) PS after 500 contact separation cycles without applied electric potential.

Results have been included in the revised manuscript main text:

"Similarly, contact between chemically smooth and rough PS films yield positive charge on the rougher and negative on the smoother surface. This trend was also confirmed for the present PS samples by KPFM measurements after 500 contact-separation cycles without applied external potential, where smooth PS showed an average surface potential of −2.11 V and rough PS showed an average surface potential of +0.491 V (Figure S9). Although these post-contact measurements do not directly probe the in-contact flexoelectric field, they confirm the expected roughness-dependent polarity of the resulting surface potential."

- Quantitative comparison between the estimated flexoelectric field and the externally applied electric field would also be necessary.

Answer: Flexoelectric fields are highly localized at nanoscale asperities, whereas our experimental setup applies a uniform macroscopic field. To bridge this gap and validate our approach, we have now performed a quantitative comparison using the experimental voltage and film thickness values together with the numerically derived surface-normal electric field from our FEM models.

For a representative 100 μm polymer film under a 3 kV bias, the external field is approximately 3×10^7 V/m. In the flexoelectric simulations at 15 nm displacement, the average surface-normal field is 4.3×10^7 V/m for the rougher contact geometry (r = 50 nm) and 1.9×10^7 V/m V/m for the smoother geometry (r = 250 nm), while the corresponding peak local values are 2.3×10^9 V/m and 3.7×10^8 V/m.

This shows that the externally applied field is comparable to the surface-averaged flexoelectric field and therefore can plausibly bias the interfacial charge-separation process, while the local flexoelectric field is strongly amplified at nanoscale asperities.

The quantitative comparison has now been included in the revised main text as a separate paragraph:

"To relate the macroscopic applied field to the simulated flexoelectric fields, their magnitudes were compared using the experimental voltage and film thickness. For a representative 100 μm polymer film under a 3 kV bias, the external field is approximately 3×10^7 V m^{-1}. In the flexoelectric simulations at 15 nm displacement, the average surface-normal electric field was 4.3×10^7 V m^{-1} for the rougher contact geometry (r = 50 nm) and 1.9×10^7 V m^{-1} for the smoother geometry (r = 250 nm), while the corresponding peak local fields reached 2.3×10^9 V m^{-1} and 3.7×10^8 V m^{-1}, respectively. Thus, the externally applied field is comparable to the surface-averaged simulated flexoelectric field, while local flexoelectric fields are strongly amplified at nanoscale asperities."

Moreover, although previous studies have suggested that flexoelectricity may contribute to triboelectric charge generation, demonstrating that the external electric field and flexoelectric fields can cancel or reinforce each other requires much stronger theoretical modeling and experimental validation than currently provided. Therefore, the proposed interpretation linking the experimental observations to flexoelectricity remains speculative.

Answer: We show contact electrification between identical polymer films where the electrons cannot penetrate through. The charge species are organoions in polymers. The driving force for organoions is external electric field as we show here. In real life flexo effects at the interface could be source for electric field for organoion transfer. It is shown before that identical polymer pairs with different roughness charges and despite the chemical composition of the polymer, the rough charges positively.

To demonstrate the cancellation and reinforcement of applied DC fields on the flexoelectricity, we developed a coupled finite element model mapping both the solid mechanics of the 15 nm asperity compression and the resulting electrostatic superposition.

To mirror our experimental macroscopic field of 30 MV/m, we applied a corresponding bias of ±90 V across our computational domain. The extracted dynamic data at the nanoscale contact interface is presented in the figures below:

Quantitative Field Superposition (Figure S10 A). We extracted the averaged normal component of the electric field (E_n) at the exact contact interface as a function of displacement.

- Baseline (0 V): The purely mechanical strain gradient generates a localized flexoelectric field that reaches -3.73×10^{8} V/m at 15 nm of compression.
- Reinforcement (-90 V): When the external macroscopic field aligns with the flexoelectric polarization, the localized field at the asperity is quantitatively boosted to -5.21×10^{8} V/m.
- Cancellation (+90 V): When the macroscopic field opposes the flexoelectric polarization, the localized field is actively suppressed to -2.26×10^{8} V/m.

Mechanistic Validation (Figure S10 B): To prove *how* these fields interact, we plotted the averaged normal polarization (P_n). The external field instantly induces a constant baseline dielectric polarization (visible as the immediate vertical shift at 0 nm displacement). However, as the mechanical compression occurs, all three curves exhibit the exact same nonlinear flexoelectric growth profile.

This theoretical modeling directly provides the precise V/m quantitative comparison and definitively proves that a macroscopic external field physically penetrates the polymer to cancel or reinforce the highly localized flexoelectric field at the contact interface.

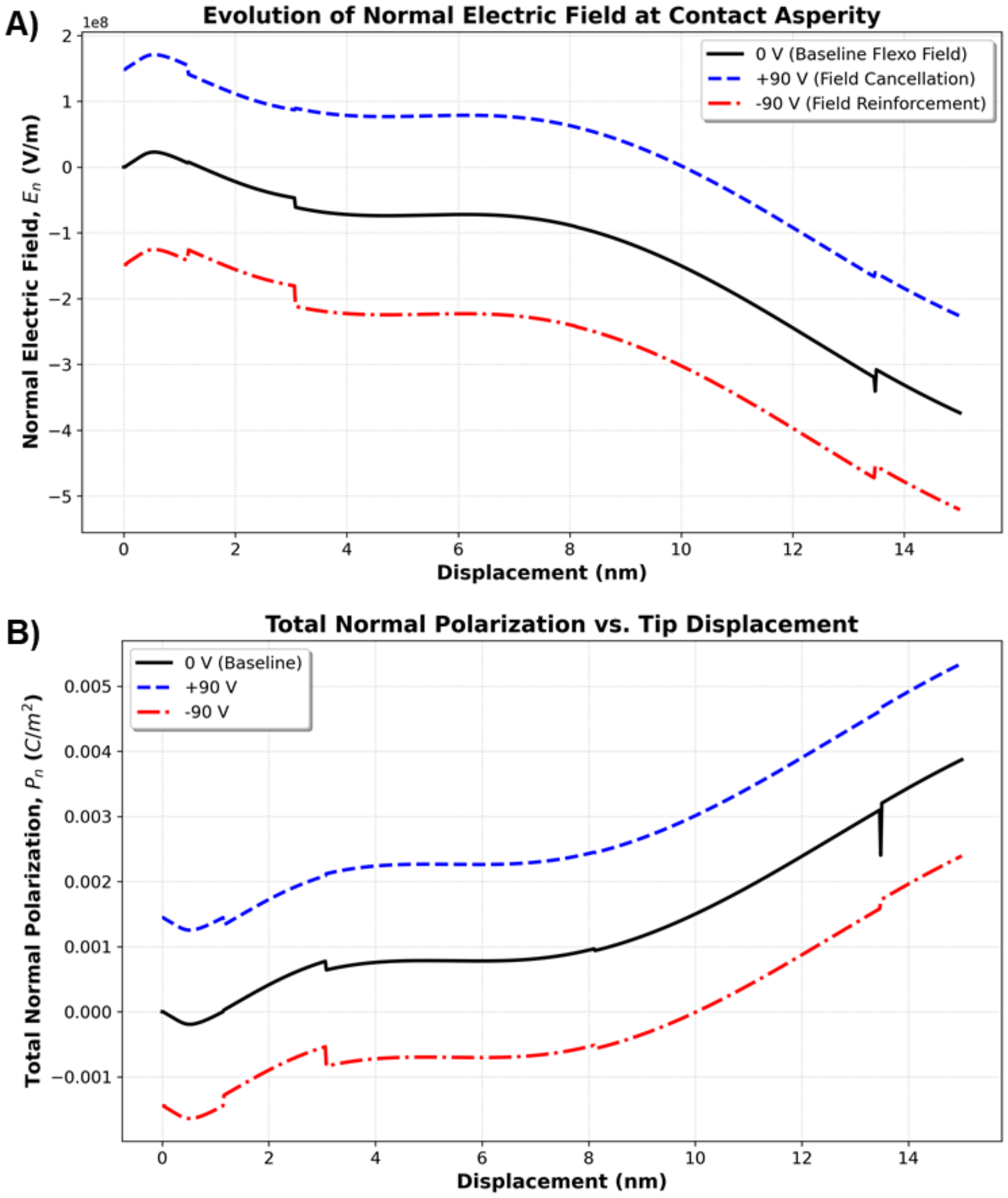

Figure S10. Biased flexoelectric-field simulations at the contact asperity. A) Averaged normal electric field, E_n, as a function of compressive displacement under 0 V, +90 V, and −90 V bias. B) Corresponding averaged normal polarisation, P_n, showing a bias-induced offset superimposed on the displacement-dependent flexoelectric response.

These results have been included in SI as a separate discussion "Biased flexoelectric-field simulations":

"Additional biased simulations were performed to examine whether an externally applied macroscopic field can reinforce or oppose the local flexoelectric field

generated at a compressed asperity. The same coupled solid mechanics/electrostatics model was used as in the main flexoelectric simulations, with a 15 nm maximum compressive displacement. To approximate the experimental macroscopic field strength of approximately 30 MV m^{-1}, external biases of ±90 V were applied across the computational domain. The normal electric field and normal polarisation were then extracted at the contact interface as a function of displacement.

In the unbiased case (0 V), mechanical compression alone generated a localized flexoelectric field at the asperity. The averaged normal electric field, En, increased with displacement and reached -3.73×10^8 V m^{-1} at 15 nm compression (Figure S10 A). When the applied field was aligned with the flexoelectric field (−90 V), the local interfacial field was reinforced, reaching -5.21×10^8 V m^{-1} at the same displacement. Conversely, when the applied field opposed the flexoelectric field (+90 V), the local field was partially suppressed, reaching -2.26×10^8 V m^{-1}. These results show that the externally applied field does not replace the mechanically generated flexoelectric field, but shifts its magnitude depending on field polarity.

The corresponding normal polarisation, P_n, provides the mechanistic explanation for this behaviour (Figure S10 B). The applied bias produces an immediate dielectric-polarisation offset even at zero displacement, visible as a vertical shift between the curves. Upon compression, however, all three cases show the same displacement-dependent increase associated with the strain-gradient-induced flexoelectric contribution. Thus, the total interfacial polarisation consists of two superimposed terms: a field-induced dielectric component and a mechanically induced flexoelectric component. This supports the interpretation that an external macroscopic field can penetrate the dielectric contact geometry and either reinforce or oppose the localized flexoelectric field at the asperity, depending on polarity."

Response to Reviewers

Reviewer #2 (Remarks to the Author):

The authors have provided detailed comment from last revision, but there are some concerns left.

- The abstract still mentions "for the first time" of field-induced inversion of CE charge. Field-driven sign inversion has been shown before in other configurations. The "first" should be scoped explicitly in the abstract to insulating polymer-polymer contacts, otherwise it can be read as overstated.

Author Response: Thank you for the comment. We agree that our original wording was too broad and could be interpreted that way. We have revised the abstract to make the scope clearer and to avoid suggesting that electric field induced inversion of contact-electrification polarity has never been reported. Instead, we now focus the novelty on polymer-polymer contacts, where the charge polarity can be controlled by the applied electric field together with the contact conditions and surface roughness.

Sentence has been revised as follows: "Herein, we demonstrate that electric fields, either applied externally or spontaneously generated at an interface can completely alter and even invert surface charge measured after contact electrification in polymer-polymer contact."

- I have concerns that FEM result might be biased. A linear coupled electrostatics-mechanics model will superimpose an applied bias on a strain-gradient field by construction. The simulation therefore confirms an assumption rather than testing the mechanism. The authors should remove any claim that this simulation proves flexoelectric drive, and state plainly that the flexoelectric link is a supported hypothesis.

Author Response:

We agree with the reviewer. The FEM model is not intended to prove flexoelectricity as the sole mechanism. Rather, it tests whether strain-gradient-induced interfacial fields of realistic magnitude can arise under the experimental contact geometry and whether these fields are consistent with the observed charge trends. We have therefore revised the wording to state that the simulations support a flexoelectric-link hypothesis, rather than prove a flexoelectric mechanism.

In the manuscript, we rephrased “with the support of well-designed experimental studies and theoretical simulations, we have shown that this driving force is a flexoelectric field” by “the simulation supports the plausibility of a flexoelectric

contribution by showing that strain-gradient-induced interfacial fields can reach experimentally relevant magnitudes.”

- The PS-versus-PTFE mismatch was justified rather than resolved. PS apparently has no reported flexoelectric coefficient, which is a real constraint, and preparing PTFE with controlled, reproducible roughness is genuinely difficult, so neither requested route was straightforward. Even so, Fig. 2B still uses PTFE simulations to rationalize a PS measurement in Fig. 2A. The smooth-only PTFE data in Fig. S5 confirm field-dependent charging but do not provide the rough/smooth comparison that was asked for. As a result, the simulation supports the sign and the qualitative trend, not a quantitative match to the PS system. The authors should relabel and rephrase Fig. 2B and its discussion

Author Response: Thank you for bringing our attention to this. We agree that Fig. 2B should not be interpreted as a quantitative simulation of the PS experiment. We have therefore relabelled Fig. 2B in caption and revised the related discussion to clarify that it is a qualitative PTFE model of roughness caused flexoelectric field polarity. Because reported flexoelectric coefficients for PS are not available, the simulation is used only to support the expected polarity and qualitative trend, not to claim quantitative agreement with the PS measurements.

Fig. 2B caption is now labelled: "Qualitative PTFE based FEM model of roughness induced flexoelectric fields under compression (PTFE was used because robust reported flexoelectric coefficients are available, whereas corresponding coefficients for PS are not). Model is intended to indicate field polarity and qualitative trends rather than to quantitatively simulate the PS experiment in A."

The discussion in the manuscript is changed to: " The result in Fig. 2 B is used as a qualitative model rather than a quantitative simulation of the PS experiment, since reported flexoelectric coefficients for PS are not available. The model shows that deformation caused by roughness can generate interfacial fields with the polarity required to influence charge transfer, supporting a possible flexoelectric contribution."

- The field-magnitude comparison is convenient and underspecified. The external field is quoted as about 3 x 10^7 V m-1, the un-divided value, whereas Fig. S1 shows only about 2.1 x 10^7 V m-1 reaching the PS after the potential divides across the stack. The flexoelectric "surface-averaged" fields of 1.9 to 4.3 x 10^7 V m-1 are averaged over an undefined region, while peak local fields are 50 to 100 times larger. Whether the fields are "comparable" depends entirely on the averaging domain, which must be specified, and the penetrated field should be used.

Author Response:

We thank the reviewer for this important observation. We agree that a more rigorous and explicitly defined comparison is required. First, we have revised the manuscript to

use the electric field penetrating into the PS layer obtained from the dielectric-barrier simulation (Figure S1), rather than the simple V/t estimate. For the representative experimental condition of a 3 kV applied bias, the electric field within the PS layer is approximately: $E_{(ext,ps)} \approx 2.1 \times 10^7$V/m. Second, we now explicitly define the surface-averaged flexoelectric field as the average magnitude of the surface-normal electric field: $\langle E_n \rangle = 1/L \int_\Gamma E \cdot n \, ds$ where Γ denotes the first 100 nm of the contact-adjacent polymer interface measured from the contact point and L =100 nm. This averaging window was chosen because the simulated flexoelectric response is highly localized near the contact region, extending less than approximately 20 nm for the rough geometry and less than approximately 60 nm for the smooth geometry. The 100 nm averaging region therefore captures the complete active flexoelectric zone for both geometries while excluding regions where the response is negligible.
Using this definition, the simulations at 15 nm compressive displacement give:

Geometry	Max(E_n) V/m	$\langle E_n \rangle$V/m
Rough asperity (r =50 nm)	2.3×10^9	4.3×10^7
Smooth asperity (r =250 nm)	3.7×10^8	1.9×10^7

Thus, the surface-averaged flexoelectric field lies within a factor of approximately 0.9-2 of the electric field penetrating into the PS layer, while the local peak flexoelectric fields are one to two orders of magnitude larger due to nanoscale field concentration at the asperity.

We have therefore revised the manuscript to state that the applied electric field is comparable to the surface-averaged interfacial flexoelectric field, rather than implying equivalence with the highly localized peak field. This clarification provides a more rigorous basis for comparing the experimentally applied field with the simulated flexoelectric driving field.

- The Ecoflex 00-30 versus 00-50 comparison keeps the chemistry roughly constant, but difference in stiffness seems to be small. The authors should report the effect size and its uncertainty rather than a qualitative "slightly lower"

Author Response: Thank you for the comment. We agree that the stiffness difference between Ecoflex 00-30 and Ecoflex 00-50 is relatively small. A larger stiffness contrast could in principle be obtained by changing the A:B component mixing ratio, for example to soften Ecoflex 00-30 or stiffen Ecoflex 00-50. However, we did not use this approach because changing the A:B ratio would also change the curing stoichiometry and chemistry of silicone network. Since components A and B contain complementary reactive species involved in platinum-catalysed crosslinking, off-stoichiometric mixing can alter crosslink density, leave residual unreacted groups and low molecular weight molecules. This would make it difficult to separate stiffness effects from changes in material chemistry.

In the previous revision, the Ecoflex 00-50 result was obtained from a single measurement. We have now replaced the results in Figure S12 with data from three measurements for both Ecoflex 00-30 and Ecoflex 00-50. Figure S12 shows the charge density as mean with a standard deviation. The results show that the charge densities of Ecoflex 00-30 and Ecoflex 00-50 are very similar within the experimental uncertainty for both positive and negative bias. We have therefore removed the phrase “slightly lower” and revised the discussion. The Ecoflex comparison is now presented as a chemically similar silicone-elastomer control, showing that the voltage assisted polarity inversion is observed in both grades, but the small stiffness difference between them does not give a clearly resolved stiffness influence.

Text in the discussion is now corrected as: "After adding measurements from three independently prepared samples, Ecoflex 00-50 showed lower mean charge densities than Ecoflex 00-30 when ±3 kV voltage was used; however, the values overlapped within experimental uncertainty (Figure S12). Thus, this comparison suggests the expected direction of the stiffness related trend but does not clearly resolve a statistically reliable stiffness influence, likely because the stiffness difference between the two grades is small. However, the polarity inversion observed in both materials supports the reproducibility of the effect in chemically similar silicone elastomers. More generally, under the same compressive force (10 N), softer polymers are expected to deform more strongly, increasing strain gradients and the associated flexoelectric polarization. "

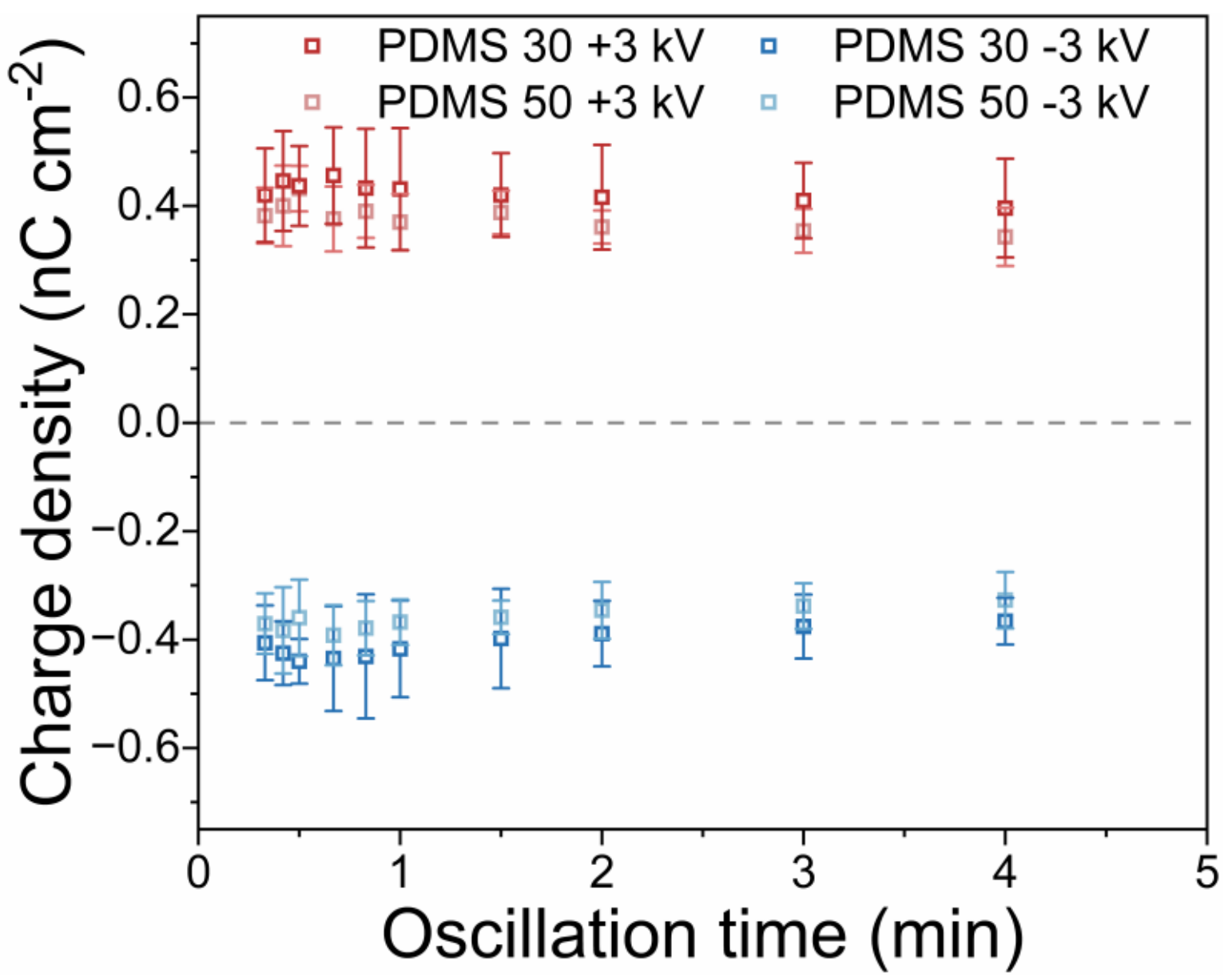

Figure S 12. Charge density comparison as a function of oscillation time for PDMS 30 and PDMS 50 elastomers after biased contact separation using ±3 kV.
